# Heavy Metal Stabilization of DNA Origami Nanostructures

**DOI:** 10.1021/acs.nanolett.3c03751

**Published:** 2024-02-16

**Authors:** Ulrich Kemper, Nicole Weizenmann, Charlotte Kielar, Artur Erbe, Ralf Seidel

**Affiliations:** † Molecular Biophysics Group, Peter Debye Institute for Soft Matter Physics, Universität Leipzig, 04103 Leipzig, Germany; ‡ Institute of Ion Beam Physics and Materials Research and Department of Nanoelectronics, Helmholtz-Zentrum Dresden-Rossendorf, 01328 Dresden, Germany; § Insitute of Resource Ecology, Helmholtz-Zentrum Dresden-Rossendorf, 01328 Dresden, Germany

**Keywords:** DNA nanostructures, DNA origami, DNA−metal interaction, seeded growth, DNA metallization

## Abstract

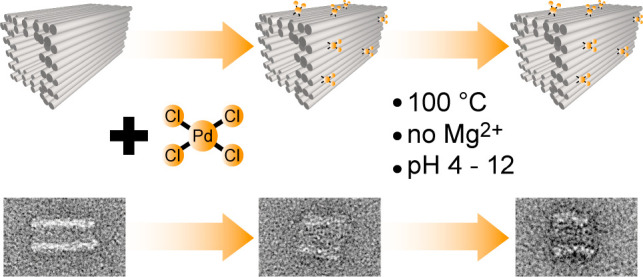

DNA origami is a powerful tool to fold 3-dimensional DNA structures with nanometer precision. Its usage, however, is limited as high ionic strength, temperatures below ∼60 °C, and pH values between 5 and 10 are required to ensure the structural integrity of DNA origami nanostructures. Here, we demonstrate a simple and effective method to stabilize DNA origami nanostructures against harsh buffer conditions using [PdCl_4_]^2–^. It provided the stabilization of different DNA origami nanostructures against mechanical compression, temperatures up to 100 °C, double-distilled water, and pH values between 4 and 12. Additionally, DNA origami superstructures and bound cargos are stabilized with yields of up to 98%. To demonstrate the general applicability of our approach, we employed our protocol with a Pd metallization procedure at elevated temperatures. In the future, we think that our method opens up new possibilities for applications of DNA origami nanostructures beyond their usual reaction conditions.

Recently, DNA nanotechnology has gained increasing interest for various applications in nanoelectronics, nanophotonics, and nanomedicine.^1−3^ One of the most employed techniques to fabricate DNA nanostructures is the DNA origami method, which allows for a bottom-up self-assembly of versatile 2D and 3D DNA nanoobjects. This is done by folding a long single-stranded DNA scaffold with the help of shorter single-stranded oligonucleotide staples.^4−6^ A major advantage of this technique is the high assembly yield and the easy functionalization of the DNA structures with chemical groups,^7,8^ biomolecules,^9−13^ and inorganic nanoparticles.^14−16^ In particular, such functionalized DNA origami nanostructures were used to build nanoelectronic components of noble metals like gold,^17−19^ silver,^20,21^ and palladium,^22,23^ less noble metals like copper,^20,24,25^ or semiconducting materials.^26−29^ Furthermore, the DNA origami technique was used to build nanoscale lithography masks to improve the spatial resolution in the fabrication of nanoelectronic and nanophotonic devices.^30,31^ Although these applications demonstrate the versatility of DNA origami, they required careful adjustment of reaction conditions to ensure the structural integrity of the DNA origami nanostructures. Pinpointing the correct conditions for optimal stability was the focus of previous studies,^32−34^ which revealed a rather narrow range of buffer conditions applicable for the folding of DNA origami. Typically, DNA origami nanostructures are assembled in a buffer solution close to neutral pH containing mM concentrations of Mg^2+^ ions. Once assembled, the presence of high Mg^2+^ concentrations plays a minor role, although the stability without Mg^2+^ strongly depends on the buffer composition.^33^ Nonetheless, pH values between 5 and 10^34^ and temperatures below ∼60 °C^35−37^ are stringently required to maintain the integrity of the structures, which sets undesired limits for applications. In the past, two different approaches have been used to widen the range of applicable conditions. In the first approach, DNA origami nanostructures were coated with inorganic calcium phosphate^38^ or silica^39,40^ or organic oligolysine^41,42^ to stabilize DNA origami structures at low Mg^2+^ concentrations, at temperatures up to 70 °C, and in physiological conditions. In the second approach, an increased stability was achieved by covalently cross-linking neighboring staple strands and/or the scaffold. Previously, we employed chemical ligation to connect the successive staple strands of an entire DNA origami nanostructure, which increased the thermal stability by ∼10 K.^36^ Gerling et al.^35,43^ employed the formation of covalent cyclobutane pyrimidine dimers between adjacent staple strands after an irradiation with UV light at 310 nm. The resulting DNA origami nanostructures were stable in low ionic strength solutions, physiological conditions, and temperatures up to 90 °C. Although the aforementioned approaches for stabilizing DNA origami nanostructures show excellent results and are highly promising, their application is quite laborious due to a time-consuming design and extensive lab work required to accurately cross-link neighboring staples. Recently, Sala et al.^44^ showed an easy and fast way of cross-linking 2D DNA origami triangles using Cisplatin. Although the AFM images showed a slight increase in stability at elevated temperatures, the heated structures were strongly deformed.

In this study, we demonstrate a simple, effective, and scalable method using [PdCl_4_]^2–^ complexes which maintained the stability of different DNA origami nanostructures even under extreme conditions. While aiming for maximum stability, we found that the base pair (bp) to Pd ion ratio plays a crucial role. Once stabilized, the DNA origami nanostructures withstood elevated temperatures up to 100 °C, double-distilled water, and solution pH values down to 4 and up to 12. Additionally, we show that our protocol is able to stabilize the sticky-end connection between multiple DNA origami nanostructure monomers as well as to bound cargos. Finally, we demonstrate that the stabilized nanostructures can be used to run a metallization reaction at strongly increased temperatures, which significantly reduced the required reaction time. In a recent study,^22^ we utilized DNA origami nanostructures as templates to cast Pd nanostructures. Thereby, we found that the precursor [PdCl_4_]^2–^ also binds to DNA, which was in agreement with previous studies.^45−47^ Inspired by this observation, we aimed to employ the precursor to cross-link neighboring strands and thus to stabilize the entire structure (Figure 1).

**Figure 1:**
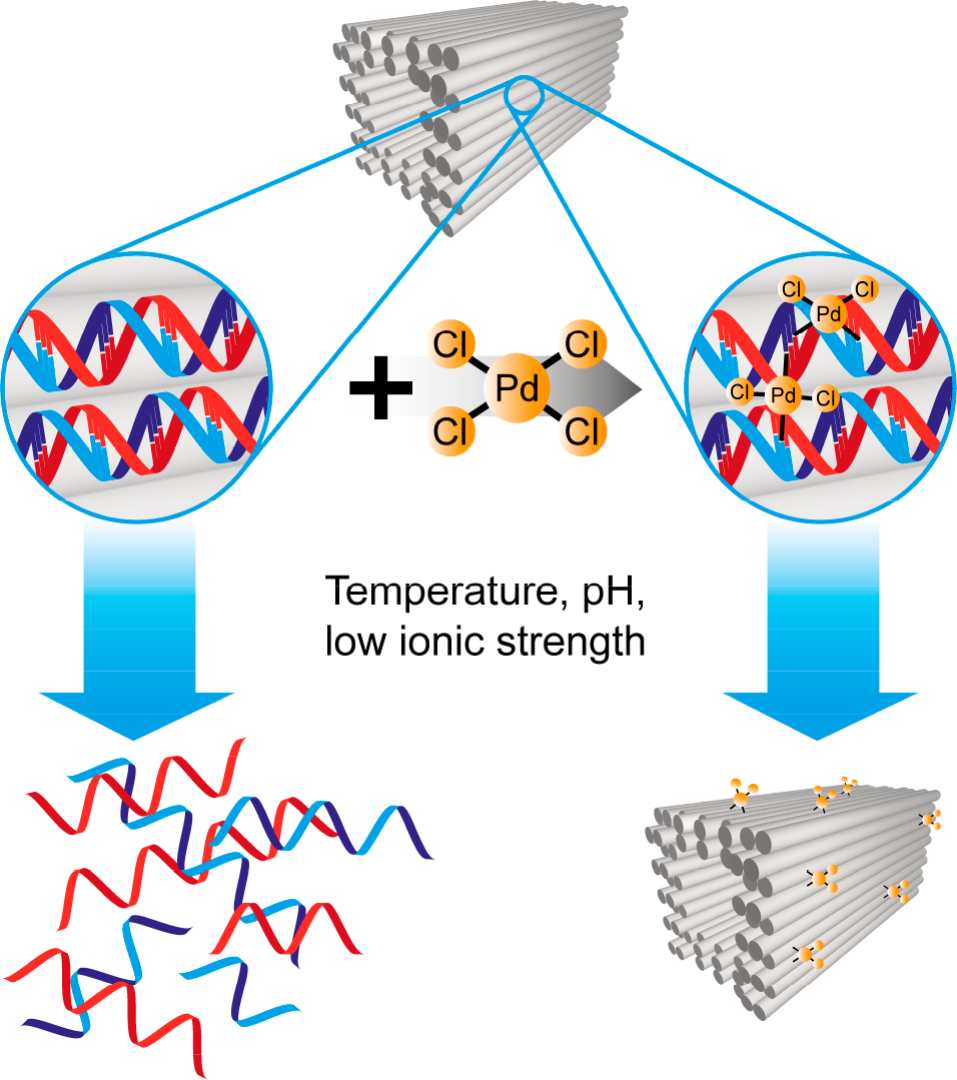
Scheme of DNA origami stabilization using the [PdCl_4_]^2–^ complexes. The Pd complex coordinates with the bases of the DNA helices and thus partially cross-links adjacent strands, which stabilizes the entire structure against harsh environmental conditions.

To test this idea, we first investigated the binding kinetics of [PdCl_4_]^2–^ to DNA. We incubated double-stranded λ-DNA with [PdCl_4_]^2–^ and measured UV–vis absorption spectra over a period of 24 h (Figure S1). We observed a pronounced shift of the DNA absorption peak from 256 to 260 nm and a decrease of its amplitude to 80% of the initial value, which we attributed to the binding of [PdCl_4_]^2–^ to the DNA.^48,49^ To quantitatively describe the observed time trajectory, we assumed the reaction to occur with second-order kinetics (Supplementary Note 1). A fit of the analytical expression for the time-dependent concentration of the palladium–DNA complex to the measured maximum absorbance over time showed excellent agreement (Figure S1, inset). The fit provided a binding rate constant of *k* = 2.77 ± 0.01 M^–1^ s^–1^, revealing that at the applied conditions (see the Methods section) 99% of the initial Pd complexes are coordinated to the DNA within 6–9 h. We therefore chose an overnight incubation time for the following experiments.

To study the ability of [PdCl_4_]^2–^ to stabilize DNA origami, we chose a previously introduced DNA origami nanotube^50−52^ as test structure (Figure 1). As a starting point, we mixed the nanotubes with a high excess of Pd ions at a ratio of 1:20 (bp:Pd ions) and tested the thermal stability of untreated and Pd incubated DNA origamis by gel electrophoresis (Figure S2a). The untreated samples disassembled at a temperature of ∼55 °C as seen by a clear upward shift of the bands corresponding to the nanotubes and the appearance of bands showing free staple strands. In contrast, the bands of the [PdCl_4_]^2–^ incubated samples did not shift up to 80 °C, and no free staples were observed. However, the intensity of the nanotube bands decreased with higher temperatures, and samples treated with 60 °C or higher temperatures showed strong aggregation. We assumed that the high [PdCl_4_]^2–^ concentration led to cross-links between the nanotube structures and consequently lowered the [PdCl_4_]^2–^ concentration to a bp:Pd ion ratio of 1:1 in a following experiment (Figure S2b). Analysis by gel electrophoresis revealed no strong decrease in aggregation. When decreasing the Pd concentration to a 2:1 ratio no aggregation could be observed even up to 100 °C (Figure 2a). Furthermore, compared to the untreated samples, the nanotube bands migrated slightly faster, and their intensity weakened with increasing temperature.

**Figure 2:**
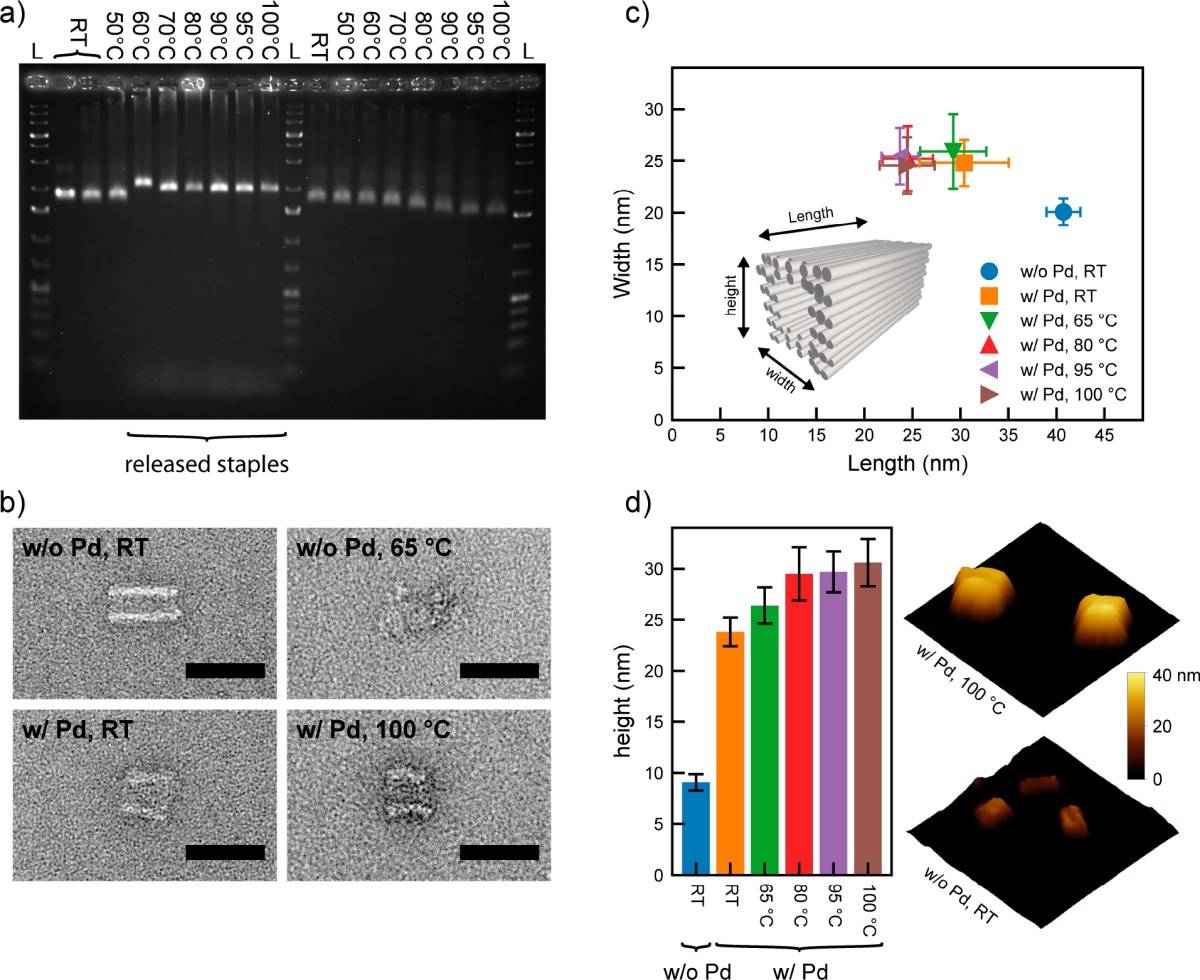
Influence of [PdCl_4_]^2–^ binding on the shape and stability of DNA origami nanotubes at a 2:1 ratio (bp:Pd ions). (a) Nanotubes incubated at different temperatures before (left) and after (right) Pd stabilization analyzed by gel electrophoresis. The temperatures are denoted above the gel, and L indicates a 1 kb ladder. (b) TEM images of nanotubes without and with Pd stabilization after incubation at different temperatures. Scale bars are 50 nm. (c) Influence of Pd binding and temperature on the length and width of nanotubes evaluated by TEM imaging. (d) Influence of Pd binding and temperature on the height of nanotubes as measured by AFM imaging in liquid. The 3-dimensional AFM images show nanotubes without (bottom) and with Pd stabilization (top). The stabilized sample was additional heated to 100 °C. Image sizes are 200 × 200 nm^2^.

TEM imaging verified the structural integrity of the nanotubes over the entire temperature range, albeit the Pd binding caused a decrease of the length and an increase of the width of the structures (Figure 2b,c, Table S1, and overview images in Figures S3–S6). Interestingly, the length decreased further with an increase in temperature, while the width stayed rather constant at ∼25 nm. We attribute both the length reduction and the broadening to structural changes due to the Pd binding. Chemically, Pd behaves similarly to Pt for which similar ligand complexes are known. The well-studied Cisplatin coordinates with the N7 of purines by replacing its chloride ligands by the nitrogen of the bases.^53^ Previous X-ray^54^ and NMR^55^ studies of Cisplatin bound to duplex DNA revealed that Cisplatin adducts introduce kinks in the double helix. Similar, [PdCl_4_]^2–^ coordinates with the N7 of purines or the N3 of pyrimidines.^56,57^ Because both Pt and Pd build planar complexes, [PdCl_4_]^2–^ adducts will also be prone to kink the DNA helix. These kinks cause shrinkage along the helical pitch and broadening in the lateral direction, consistent with the deformation of the nanotubes upon Pd binding. To understand the temperature dependence of the structural changes, one has to consider that [PdCl_4_]^2–^ binds as a monoadduct to single bases or can cross-link adjacent bases as a higher order adduct. We think that heating the structures leads to local denaturation along the DNA helices and allows monoadducts to form higher order adducts. This causes a shortening of the nanotubes as well as a faster migration and a weakening of the intensity of the gel bands. This was further supported by AFM measurements in liquid (Figure 2d and overview images in Figures S8–S13). The AFM images confirmed the general trend of the deformations of the nanotubes upon Pd binding and subsequent heating in length and width (Table S2). In contrast to TEM imaging, AFM also provided the height of the adsorbed structures. For the Pd stabilized structures the height values agreed well with the widths measured by TEM, while the height for the nonstabilized structure was much lower than the width. This could be either due to the high flexibility of the nonstabilized nanotubes, such that they became squeezed by the AFM tip, or due to a collapse of the structures along their diagonal upon adsorption, resulting in a sheet of four DNA helices (see Figure S8). Altogether, the Pd stabilized nanotubes appear to better maintain their 3-dimensional structure and thus exhibit a higher resistance against mechanical compression.

In a next series, we determined a bp:Pd ion ratio of 4:1 to be the minimal amount of Pd necessary to stabilize DNA origami nanostructures (Supplementary Note 2 and Figures S14–S17). However, to ensure maximum stability, we employed the 2:1 ratio in the following experiments.

To test the general applicability of our stabilization technique, we next aimed to stabilize differently shaped DNA origami nanostructures. To this end, we employed flag structures^58^ consisting of either a 6- (F6HB) or a 10-helix-bundle pole (F10HB) as well as a rectangular blade formed by three DNA layers (Figure 3a,b). We incubated these structures with [PdCl_4_]^2–^ and subjected them to elevated temperatures. Gel electrophoresis revealed that for the [PdCl_4_]^2–^ treated samples no staple strands were released over the entire temperature range (Figure S18), while for the untreated samples the band was clearly shifted upward at 65 °C, indicating disassembly, for the stabilized samples the band shifted slightly downward with increasing temperatures. TEM imaging confirmed the structural integrity of [PdCl_4_]^2–^ stabilized flag structures even up to 95 °C (Figures S19–S26). As before, we observed a shrinkage along the helical pitch and a broadening in the perpendicular direction (Figure 3c and Table S3).

**Figure 3:**
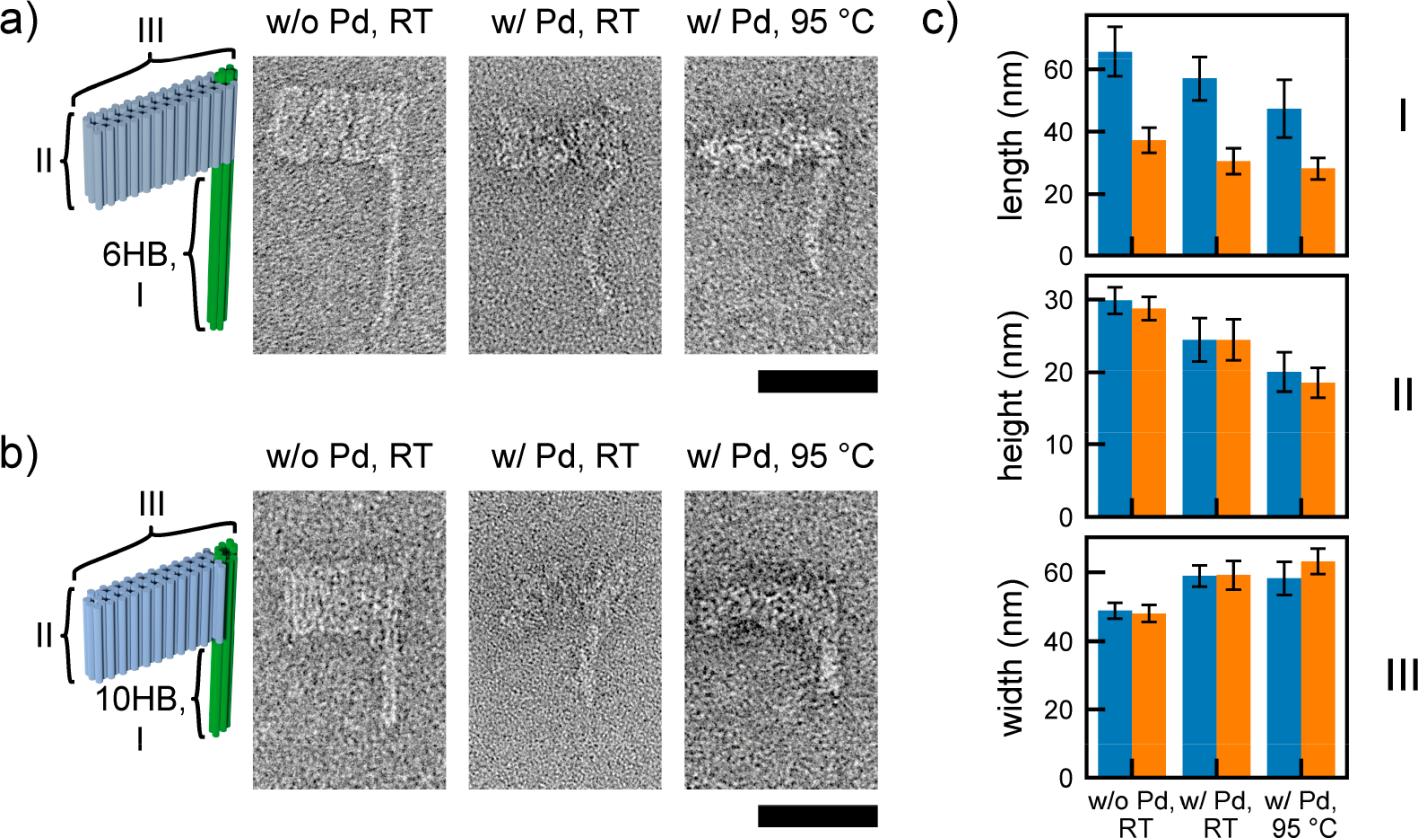
Influence of Pd binding on the shape and stability of DNA origami flag structures. (a) TEM images of F6HB. (b) TEM images of F10HB. (c) Change in dimension of F6HB (blue) and F10HB (orange) upon Pd binding and a subsequent temperature treatment: (I) pole length, (II) blade height, and (III) blade width. Scale bars = 50 nm.

Additionally, Pd binding caused occasional strong bending of the F6HB pole (Figures S21 and S22), whereas the F10HB pole retained a rather straight appearance (Figures S25 and S26). We again assigned the shrinkage along the helical pitch and the broadening in the lateral direction to kinking of the DNA helices by bound [PdCl_4_]^2–^. The similarities between the deformations of the tube structures and the flag structures strengthen this interpretation.

Having established a protocol to stabilize DNA origami nanostructures against thermal denaturation, we were next interested whether [PdCl_4_]^2–^ can also stabilize DNA origami nanostructures against harsh buffer conditions, e.g., low ionic strength and more extreme pH values. We first assembled DNA nanotubes and afterward performed a dialysis against either double-distilled water, an acetate buffer at pH 4 or 5, or a boric buffer at pH 11 or 12. For nonstabilized nanotubes in double-distilled water gel electrophoresis and TEM imaging displayed rather intact nanotubes (Figures 4 and S28). Although Mg^2+^ in the mM range was considered for a long time as a main factor to ensure stable DNA origami nanostructures, Kielar et al.^33^ demonstrated that once assembled DNA origami nanostructures can be transferred to low ionic strength buffers without losing their structural integrity due to residual Mg^2+^ bound to the DNA backbone. However, subsequent heating led to disassembly of the nanotubes. In contrast, [PdCl_4_]^2–^ treated nanotubes remained their integrity in double-distilled water over the entire temperature range (Figures 4 and S29–S30).

**Figure 4:**
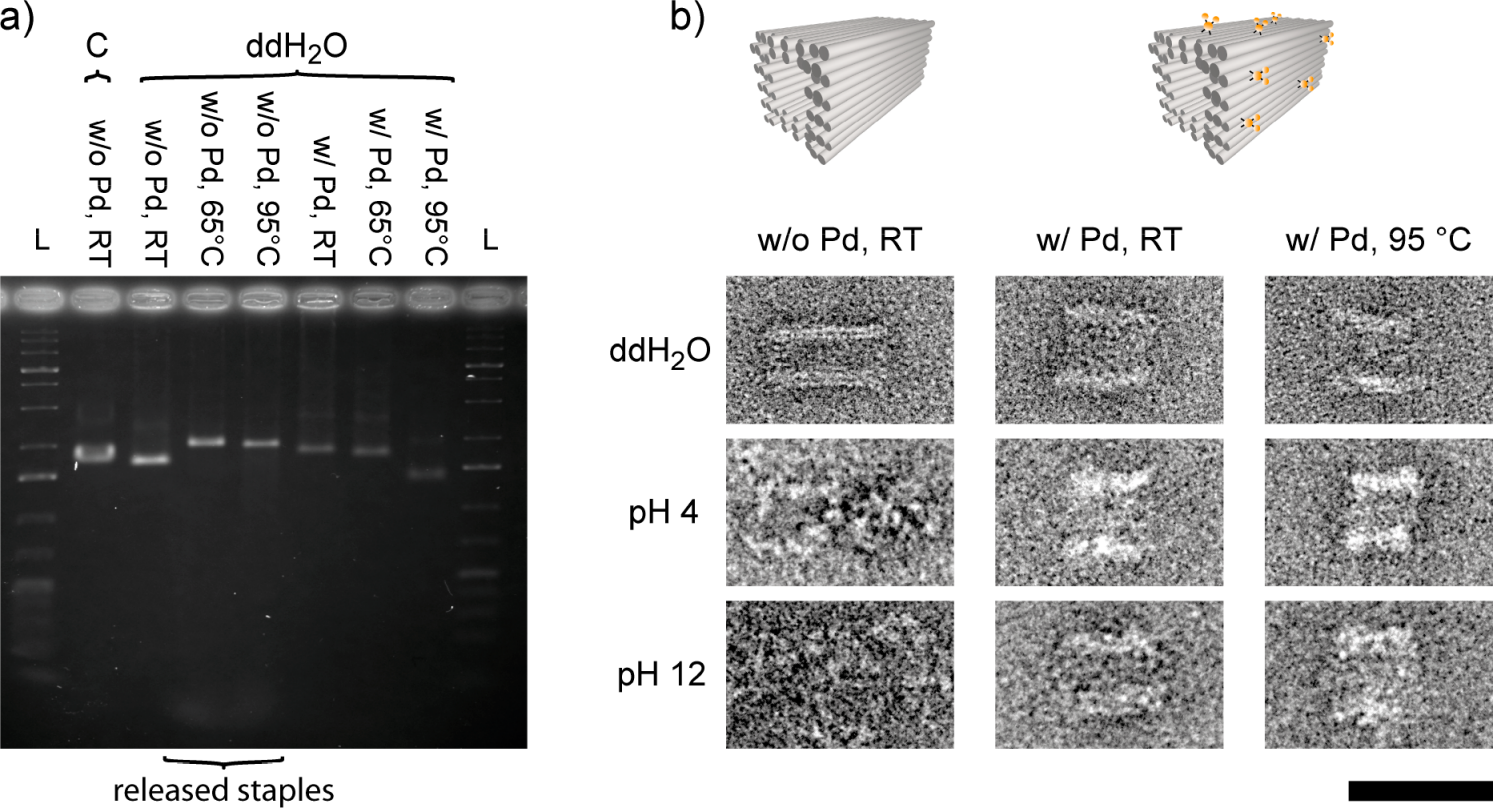
Pd stabilization of DNA origami nanostructures in double-distilled water and extreme pH at different temperatures. (a) Nanotubes incubated in double-distilled water at different temperatures before (left) and after (right) Pd stabilization analyzed by gel electrophoresis. C is the nanotube control, and L indicates a 1 kb ladder. (b) TEM images of nanotubes incubated in double-distilled water and extreme pH at different temperatures. Scale bar = 50 nm.

The studies of Wang et al.^34^ revealed that tubular DNA origami nanostructures are stable at pH values between 5 and 10. If the environment becomes more acidic or alkaline, the hydrogen bonds between the DNA base pairing break due to protonation or deprotonation of the bases. Consequently, we investigated whether our Pd stabilization protocol can conserve DNA origami nanostructures at more extreme pH values. Electrophoresis gels of nanotubes that were incubated at different pH values displayed a constant band height at RT for untreated nanotubes at pH 5 and 11 (Figure S27). At pH 4 and 12 the bands were shifted downward, and TEM images confirmed a disassembly of the nanotubes at these pH values (Figures S31 and S34). Again, DNA origami nanostructures stabilized with [PdCl_4_]^2–^ remained intact at these denaturing conditions at RT and even at 95 °C (Figures 4, S32–S33, and S35–S36). Because the low- and high-pH buffers both lacked Mg^2+^, we demonstrated with these experiments that [PdCl_4_]^2–^ stabilized DNA origami structures remain intact independent of the ionic strength and temperature at pH values ranging from 4 to 12.

Having demonstrated the capability of [PdCl_4_]^2–^ to stabilize DNA origami nanostructures, we were next interested whether the [PdCl_4_]^2–^ treatment can also preserve the functionalization of DNA origami nanostructures. The nanotubes, so far employed, can be functionalized either with sticky ends to form specific orthogonal interfaces for the assembly of linear superstructures with defined lengths^22,51,52^ or with protruding single strands in their cavities to attach cargos like inorganic nanoparticles^22,50,51^ or biomolecules like BSA^59^ or streptavidin.^9^

Using two different interfaces, we assembled nanotube trimers with a yield of 89 ± 2% (*N* = 239). After treatment of the trimers with [PdCl_4_]^2–^ and a subsequent heating to 95 °C, the yield of correctly formed trimers changed to 82 ± 4% (*N* = 259) and 78 ± 3% (*N* = 232), respectively (Figure 5a and overview images in Figures S37–S39). Thus, 88% of the correctly formed trimers were still intact after the thermal treatment. Of note, these yields were achieved using sticky ends employing 3 nt overhangs. We are confident that the yield can be improved using stronger interfaces, e.g., by using 5 nt overhangs.^52^ Next, we tested the stabilization of cargo attachment using gold nanoparticles (AuNPs) as reference cargos. Therefore, we employed AuNPs being covered with a dense brush of ssDNA strands, enabling their binding to four single strands in the center of the nanotube cavity. Because of the high density of DNA around the AuNPs, the binding of [PdCl_4_]^2–^ should lead to a strong cross-linking of the ssDNA brush around the AuNPs, the protruding strands, and the inner nanotube walls. In agreement with this hypothesis, TEM images displayed that the yields of AuNP attachment were maintained (Figure 5b, Table S4, and overview images in Figures S40–S42). As a control, we tried to attach AuNPs to Pd prestabilized nanotube monomers. A prior [PdCl_4_]^2–^ binding causes a collapse of protruding ssDNA^20,23^ and, hence, should suppress a hybridization of complementary strands. As expected, we observed almost no AuNP binding inside the nanotube cavities (Figure S43).

**Figure 5:**
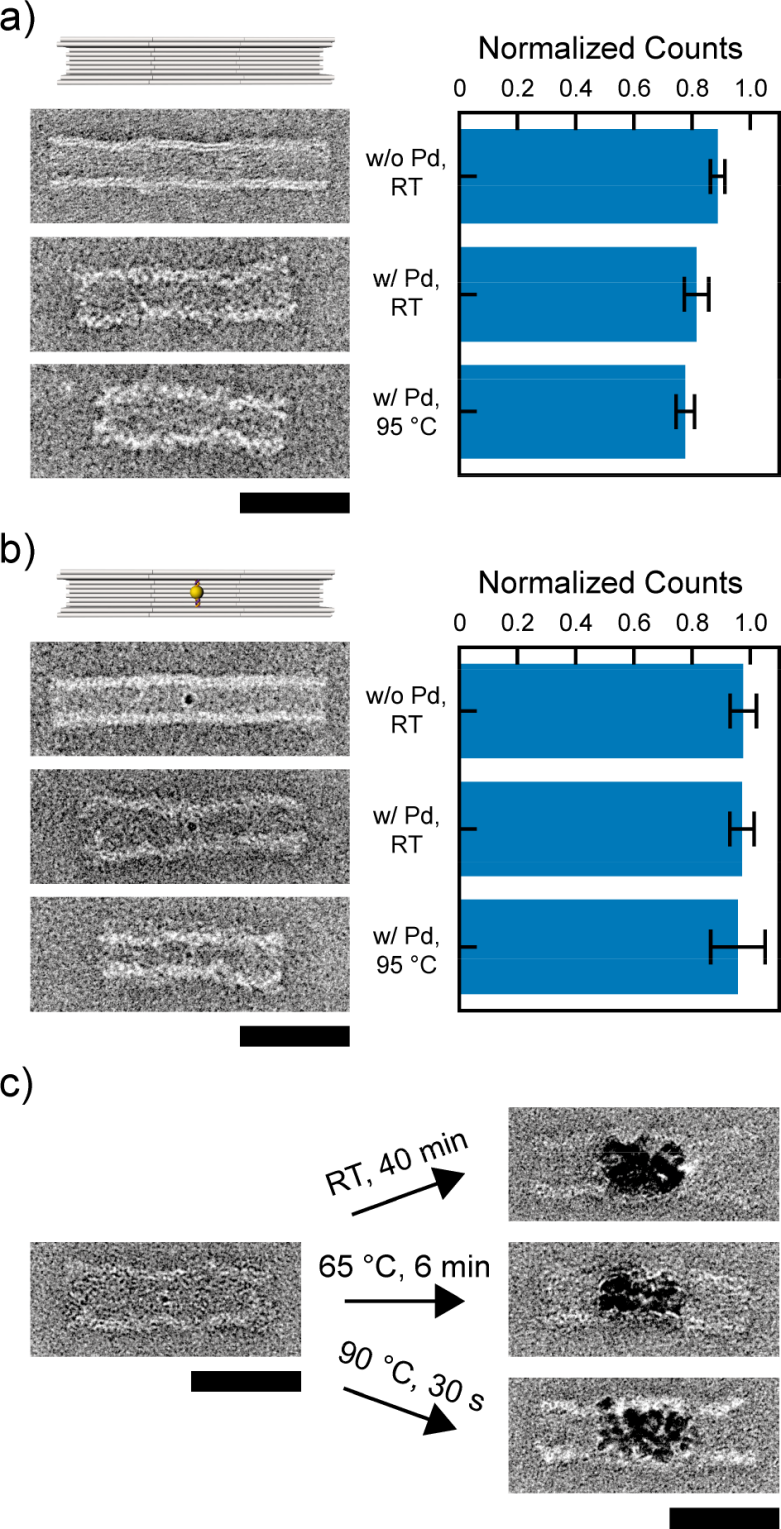
Influence of [PdCl_4_]^2–^ on the stability of DNA origami multimers, the attachment of cargos, and Pd metallization. (a) TEM images and histogram of correctly formed trimers. (b) TEM images and histogram of AuNPs bound to the trimer center. (c) TEM images of Pd metallized trimers. Utilizing the stability enhancement of the DNA origami the metallization kinetics could be vastly accelerated, yielding similar results after 40 min at RT and after 30 s at 90 °C. Scale bars = 50 nm.

To demonstrate the benefit of our Pd stabilization protocol for DNA origami nanostructure applications, we tested whether we can make use of the heat stability of the modified DNA nanotubes. To these end, we employed our stabilized tube trimer structures in a DNA mold-based palladium nanoparticle (PdNP) growth procedure that we established previously.^22^ Single PdNP seeds were attached in the centers of the trimer structures, which were subsequently stabilized with [PdCl_4_]^2–^. Then we initiated seeded growth of Pd from the central PdNP seed in which the nanotube walls determined the shape of the resulting structure. First, we applied the growth procedure at RT as described previously^22^ and fabricated slightly rod-like Pd structures inside the nanotube channels with a reaction time of 40 min (Figure 5c). This procedure led to a highly homogeneous growth around the central PdNP, without nonspecific nucleation at [PdCl_4_]^2–^ bound along the nanotube walls (Figure S45). After demonstrating that we can use our previously established Pd growth protocol on Pd stabilized nanotubes, we repeated the procedure at elevated temperatures. At 65 °C we got similar results as at RT but within a reaction time of only 6 min as estimated from a pronounced color change of the growth solution from yellow to dark brown (Figures S44 and S46). At 90 °C the reaction was completed within 30 s. At this temperature, in addition to the rod-like Pd structures that grew inside the nanotube channels, some metal grains started to grow through the nanotube walls (Figure S47) in contrast to the lower temperatures. This is in agreement with our previous findings on the growth of Au^18^ and Pd,^22^ which suggested that the soft DNA walls best guide the growth of metal nanostructures at reduced reaction rates.

In conclusion, we established a simple and effective method to stabilize DNA origami nanostructures under harsh application conditions. Our approach utilizes the strong affinity of Pd to nitrogen atoms, such that the precursor [PdCl_4_]^2–^ could cross-link adjacent DNA helices and thus stabilize the entire structure against strong denaturing conditions. DNA origami structures incubated with [PdCl_4_]^2–^ in a 2:1 ratio (bp:Pd ions) exhibited a higher resistance against mechanical compressions and withstood temperatures up to 100 °C, low ionic strength buffers, and pH values down to 4 and up to 12. Although once stabilized, the structures were incapable of performing further strand hybridizations; complex DNA structures that were preassembled prior Pd binding were highly stabilized. This was exemplified by nanotube trimers and attached AuNPs. Finally, we demonstrated the applicability of the stabilization by performing a Pd growth procedure at elevated temperatures, resulting in an 80-fold faster reaction at 90 °C. In principle, other transition metals like Cu, Ag, and Pt that bind to the DNA bases may show similar stabilization behavior and could be the focus of further studies. We are confident that the heavy-metal-based stabilization of DNA origami structures has great potential in the DNA origami-based bottom-up fabrication of electric circuits. So far, only noble metals like gold,^17−19^ silver,^20,21^ palladium,^22,23^ and the semi-noble-metal copper^24,25^ have been grown on DNA origami templates. However, to recreate all aspects of electric circuits, the deposition of less noble materials, e.g., nickel, would be required. Unfortunately, incorporating such materials would require harsh conditions under which DNA origami structures disassemble.^60,61^ Utilizing our Pd stabilization procedure, an incorporation of these materials can potentially be realized.

## Supporting Information Available

Materials and methods, a detailed determination of the Pd–DNA binding kinetics and additional TEM, AFM and agarose gel images (PDF)
