# Supplementary material for: Heavy Metal Stabilization of DNA Origami Nanostructures
Source: Nano Lett. 2024 Feb 16;24(8):2429–36. doi: 10.1021/acs.nanolett.3c03751 (PMC10905993; doi:10.1021/acs.nanolett.3c03751)
Supplement: Supplementary file 1 — nl3c03751_si_001.pdf [file nl3c03751_si_001.pdf]

Supporting Information

Heavy metal stabilization of DNA origami nanostructures

*Ulrich Kemper[a], Nicole Weizenmann[a], Charlotte Kielar[b,c], Artur Erbe[b] and Ralf Seidel[a]**

[a]Molecular Biophysics group, Peter Debye Institute for Soft Matter Physics,

Universität Leipzig, 04103 Leipzig, Germany

*E-mail: ralf.seidel@physik.uni-leipzig.de

[b]Institute of Ion Beam Physics and Materials Research and Department of Nanoelectronics, Helmholtz-Zentrum Dresden-Rossendorf, 01328 Dresden, Germany.

[c]Insitute of Resource Ecology,

Helmholtz-Zentrum Dresden-Rossendorf, 01328 Dresden, Germany

Materials and Methods

Binding Kinetic Studies

0.825 nM λ-DNA was incubated with 40 μM $[PdCl_4]^{2-}$ in a boric buffer (BB) containing 0.5 M boric acid and 11 mM $MgCl_2$ (pH 8) to yield a 1:1 (bp:Pd ions) ratio. Directly after mixing a series of absorption spectra (every 5 min for 24 h) was recorded using a Cary 60 (Agilent) absorption spectrometer. To quantitatively describe the observed time trajectory, the reaction was fitted according to a second order reaction kinetic $A + B \rightarrow P$, where A is $[PdCl_4]^{2-}$, B a DNA base and P the reaction products (see Supplementary Information Note 1 for a detailed description).

DNA Origami Assembly

Nanotube and flag designs were used as reported in previous studies.[1,2] DNA origami structures were assembled in a one-pot reaction by mixing 10 nM p8064 ssDNA scaffold (Eurofins), 100 nM staple oligonucleotides (Eurofins), and 10 nM nanoparticle capture strands in a folding buffer (FB) containing 5 mM Tris-HCl, 1 mM EDTA, 11 mM $MgCl_2$, and 5 mM NaCl (pH 8, Eurofins). Subsequently, the solution was heated to 80 °C for 5 min and cooled down to 25 °C using a nonlinear temperature ramp over 15 h, with the slowest temperature decrease occurring between 55 °C and 45 °C.[3] The resulting nanotubes and flags were purified from excess staples using polyethylene glycol (PEG) precipitation and were resuspended in BB. The concentration of the DNA origami structures was determined from the DNA absorbance at 260 nm. Absorption spectra were recorded using a NanoPhotometer P-Class P 330 (Implen).

Pd Stabilization of DNA Origami Structures

An aqueous stock solution of 10 mM $[PdCl_4]^{2-}$ was prepared by dissolving 44.5 mg $PdCl_2$ (Sigma-Aldrich) in 25 ml HCl (20 mM) in a water bath at 50 °C overnight and stored in the dark at 4 °C. An aliquot of the stock solution was diluted to 1 mM directly before usage. DNA origami structures in BB were incubated overnight with $[PdCl_4]^{2-}$ to yield a final nominal concentration of DNA origami structures of 6 nM. The final $[PdCl_4]^{2-}$ concentration differed on the experiment (see main text). Finally, to remove excess $[PdCl_4]^{2-}$ the stabilized structures were dialyzed against 2 l of BB using 2 kDa cut-off dialysis units (Slide-A-Lyzer® MINI, ThermoFischer Scientific).

Stability Tests

For the thermal stability tests, 20 µl of Pd stabilized DNA origami structures were incubated for 10 min at elevated temperatures (see main text) in a thermomixer (Thermomixer comfort, Eppendorf). Samples were then cooled for 5 min on ice to suppress any subsequent reactions. For low ionic strength and pH stability tests, 100 µl of Pd stabilized DNA origami structures were dialyzed against 2 l of the respective buffer. The pH 4 buffer contained 22.7 mM of sodium acetate and 77.6 mM lactic acid. The pH 5 buffer contained 67.3 mM of sodium acetate and 32.7 mM lactic acid. For alkaline buffers the pH of 0.5 M boric acid was calibrated to pH 11 or 12 using NaOH.

DNA Functionalization of Au and PdNPs

5 nm citrate stabilized AuNPs (Sigma-Aldrich) were subjected to a ligand exchange to BSPP to increase the colloidal stability at high concentrations as described previously.[4] Briefly, 5 mg BSPP was added to 15 ml of the AuNPs suspension and the mixture was stirred in the dark overnight. Then, solid NaCl was added while stirring until the color of the suspension changed from red to purple. Subsequently, the mixture was centrifuged at 3200 rcf for 30 min and the supernatant was removed. AuNPs were then resuspended in 1 ml of 2.5 mM aqueous BSPP solution. 2 ml of methanol were added and the suspension was centrifuged at 3200 rcf for 30 min. After removing the supernatant and resuspending the AuNPs in 1 ml of 2.5 mM BSPP, the concentration of the AuNPs was estimated from the absorbance at 520 nm.

PdNPs were used as synthesized. AuNPs and PdNPs were conjugated with 15 nt or 10 nt 5´-thiol-modified polythymidine ssDNA strands (Sigma-Aldrich), respectively. First, the disulfide bonds of the oligonucleotides were reduced in 20 mM of Tris(2-carboxyethyl)phosphine hydrochloride (TCEP, Sigma-Aldrich) over night. Subsequently, they were purified using G-25 size exclusion columns (GE Healthcare) and added to the PdNPs in 0.5x TBE buffer (65 mM Tris base, 22.5 mM boric acid, 1.25 mM EDTA, pH 8) containing 11 mM $MgCl_2$ at a 500-fold or 200-fold stoichiometric excess over AuNPs or PdNPs, respectively. To densely coat the NPs with DNA, salt aging was performed. For AuNPs, NaCl was added in two steps to a final concentration of 350 mM, with a 4 h break in between. For PdNPs, the NaCl concentration of the solution was increased in 100 mM steps until the final concentration of 1 M was reached. After each step, the sample was stirred and incubated for 30 min followed by overnight incubation after the last step. Subsequently, the functionalized NPs were purified by five ultrafiltration (MWCO, Amicon Ultra,

50 kDa cut-off, Sigma-Aldrich) and washing steps using 0.5x TBE buffer containing 11 mM $MgCl_2$.

NP Loading of nanotubes and Trimer Formation

For NP loading, assembled nanotubes were resuspended in FB after PEG precipitation. DNA functionalized NPs were mixed with nanotubes in FB supplemented with 350 mM NaCl at a final nanotube concentration of 6 nM and a final NP concentration of 18 nM or 60 nM for AuNPs or PdNPs, respectively. The mixture was heated to 40 °C and cooled to 23 °C with a rate of 1 K per 17 min to allow hybridization of the NPs with the complementary capture strands within the nanotube cavity. The NP loaded nanotubes were purified from unbound NPs using PEG precipitation. For nanotube trimer formation, three DNA origami nanotubes were mixed at equal stoichiometry in FB supplemented with 350 mM NaCl and incubated overnight. Subsequently, a PEG purification was performed in order to remove excess NPs and the trimers were resuspended in BB.

Seeded Pd Growth Procedure

The concentration of seed loaded nanotube trimers was determined from the DNA absorbance at 260 nm. It was adjusted to yield a final nominal concentration of PdNP seeds of 1 nM in the growth solution. For the seeded growth, 1.1 mM $[PdCl_4]^{2-}$ and 11 mM ascorbic acid (AA) were added to the nanotube trimers in boric buffer (0.5 M boric acid, 11 mM $MgCl_2$, pH 8) while stirring the solution at different temperatures as depicted in the main text. During the growth, the mixture was vigorously stirred. A color change from light yellow to dark brown indicated the formation of Pd nanostructures (Figure S42).

Gel Electrophoresis

DNA origami structures were mixed with 6x orange G loading Dye (15 % (w/v) Ficoll® 400 (Carl Roth), Orange G (Sigma-Aldrich)). For the nanotube structures in double-distilled water, 66 mM $MgCl_2$ was added to the loading solution. The samples were then analyzed on 1.5 % agarose gels containing 0.5x TBE buffer and 11 mM $MgCl_2$ for 120 min at 80 V (7.0 Vcm^{-1}) at RT.

TEM Imaging and Image Analysis

TEM imaging was performed using a JEM2100Plus (Jeol) transmission electron microscope at an acceleration voltage of 200 kV. The microscope is equipped with a 4K CMOS camera system (TVIPS, Germany) and the measurements were done with the EMMenu 5.0 software package (TVIPS, Germany). The TEM sample preparation was performed by placing 5 µl of the sample solution onto glow-discharged carbon-coated TEM grids and incubated for 5 min followed by a washing step with 5 µl HPLC water or a staining step. For negative staining, the sample was quickly washed with a 2 % aqueous uranyl formate staining solution containing 5 mM NaOH followed by a 10 sec incubation with the staining solution. The determination of the DNA origami dimensions were performed using the software ImageJ and only sidewall-lying structures were analysed.

AFM Imaging and Image Analysis

AFM imaging was performed in liquid using the Cypher ES (Asylum Research, Goleta, CA, USA). The liquid samples were measured in tapping mode with a Biolever Mini BL-AC40TS-C2 cantilever (Olympus, Tokyo, Japan) at RT. The AFM sample preparation was performed by dropping 50 µl of the thermally treated Pd stabilized samples (RT, 65 °C, 95 °C) with a

concentration of 6 nM onto a freshly cleaved mica surface. The untreated reference sample was diluted to a nanotube concentration of 0.5 nM with 1xTAE (Carl Roth) containing 20 mM $MgCl_2$ to get better adsorption on the mica surface. The images were recorded with a scan size of 3 x 3 μm^2, 2 x 2 μm^2 and 1 x 1 μm^2, a line rate of 0.7 Hz and a resolution of 1024 x 1024 px^2 or 512 x 512 px^2. Image processing and determination of the origami height profiles was performed with the software Gwyddion 2.55. In accordance to TEM analysis, only nanotubes that were adsorbed with a sidewall to the substrate were analyzed (see Figure S7 b and c). For such nanotubes the sidewalls could be seen as two elevated parallel strips with the cavity seen as an elongated dip between them. Standing nanotubes displayed in contrast a central dip. We considered only nanotubes that could be clearly assigned and disregarded ambiguous cases. The nanotubes without Pd at RT heavily stacked on each other (Figure S8). Consequently only unstacked nanotubes were measured (Figure S8, inset). The length, width and height were determined manually for each nanotube. Width and height were determined using the Gwyddion function *Step Height* (positive) (Figure S7 a and b). The length was determined using the function *Step Height* (positive) or using the function *Measure Distances.*

Supplementary Note 1: Pd – DNA Reaction Kinetic Constant Determination

The binding of $[PdCl_4]^{2-}$ to the DNA bases was assumed to follow second order kinetics according to the reaction scheme:

$$Pd + B \rightarrow PdB + S \qquad (1)$$

where Pd represents the $[PdCl_4]^{2-}$ complex, B a DNA base, PdB the palladium-base complex and S any side products. Generally, the time dependent concentrations ($[Pd]$ and $[B]$) for this reaction scheme, employing different starting concentrations $[Pd]_0$ and $[B]_0$, can be described by the formula:[5]

$$\frac{1}{[B]_0 - [Pd]_0} \ln \frac{[Pd]_0[B]}{[B]_0[Pd]} = kt, \qquad (2)$$

where k is a second order rate constant and t the time. Utilizing the mass action law, i.e. $[B] = [B]_0 - ([Pd]_0 - [Pd])$, Equation (2) can be rewritten in terms of the starting concentrations as:

$$[Pd] = \frac{[Pd]_0([B]_0 - [Pd]_0)}{[B]_0 e^{kt([B]_0 - [Pd]_0)} - [Pd]_0}. \qquad (3)$$

When fitting the kinetics to the experimental data, we additional accounted for a linear term $(-ct)$, which becomes dominant after 10 h (see Figure S1). We attributed this linear term to unspecific binding of DNA to the cuvette walls, which has also been seen for reference measurements of λ-DNA without the addition of $[PdCl_4]^{2-}$.

The experimental data was then fitted to the extended equation (3) describing the time dependent absorbance A:

$$A = (1-d)\frac{([B]_0 - [Pd]_0)}{[B]_0 e^{kt([B]_0 - [Pd]_0)} - [Pd]_0} - ct + d, \qquad (4)$$

with d being the offset.

Curve fitting was performed using a self-written python script (Python version 3.8) using the "curve_fit" function for nonlinear least-squares optimization, which is included in the SciPy package.[6]

Supplementary Note 2: Determining the minimal amount of $[PdCl_4]^{2-}$ to stabilize DNA origami nanostructures

To find the minimal amount of Pd necessary to efficiently stabilize DNA origami nanostructures, we lowered the concentration of $[PdCl_4]^{2-}$ to a bp:Pd ion ratio of 4:1, 8:1 and 16:1 (Figure S11). Analysis by gel electrophoreses provided that all $[PdCl_4]^{2-}$ treated samples did not release staple strands upon heating and did also not show signs of disassembly. For the 4:1 and 8:1 samples again a slight downwards shift of nanotube band with increasing temperatures was observed. In the TEM images, only the nanotubes treated at a 4:1 ratio were intact after heating to 95 °C, while for ratios of 8:1 and 16:1 the nanotubes became randomly unfolded upon heating (Figures S12-14). Consequently, we infer a 4:1 ratio as the minimum amount of $[PdCl_4]^{2-}$ required for stabilizing DNA origami nanostructures against thermal disassembly.

References

1. Ye, J.; Helmi, S.; Teske, J.; Seidel, R. Fabrication of Metal Nanostructures with Programmable Length and Patterns Using a Modular DNA Platform. *Nano Letters* **2019,** *19* (4), 2707–2714.
2. Kauert, D. J.; Madariaga-Marcos, J.; Rutkauskas, M.; Wulfken, A.; Songailiene, I.; Sinkunas, T.; Siksnys, V.; Seidel, R. The energy landscape for R-loop formation by the CRISPR-Cas Cascade complex. *Nature structural & molecular biology* **2023,** *30* (7), 1040–1047.
3. Czogalla, A.; Kauert, D. J.; Franquelim, H. G.; Uzunova, V.; Zhang, Y.; Seidel, R.; Schwille, P. Amphipathic DNA origami nanoparticles to scaffold and deform lipid membrane vesicles. *Angewandte Chemie International Edition* **2015,** *54* (22), 6501–6505.
4. Helmi, S.; Ziegler, C.; Kauert, D. J.; Seidel, R. Shape-controlled synthesis of gold nanostructures using DNA origami molds. *Nano Letters* **2014,** *14* (11), 6693–6698.
5. House, J. E. *Principles of Chemical Kinetics*, 2nd ed.; Elsevier Science & Technology: San Diego, 2007.
6. Virtanen, P.; Gommers, R.; Oliphant, T. E.; Haberland, M.; Reddy, T.; Cournapeau, D.; Burovski, E.; Peterson, P.; Weckesser, W.; Bright, J.; van der Walt, S. J.; Brett, M.; Wilson, J.; Millman, K. J.; Mayorov, N.; Nelson, A. R. J.; Jones, E.; Kern, R.; Larson, E.; Carey, C. J.; Polat, İ.; Feng, Y.; Moore, E. W.; VanderPlas, J.; Laxalde, D.; Perktold, J.; Cimrman, R.; Henriksen, I.; Quintero, E. A.; Harris, C. R.; Archibald, A. M.; Ribeiro, A. H.; Pedregosa, F.; van Mulbregt, P. SciPy 1.0: fundamental algorithms for scientific computing in Python. *Nat Methods [Online]* **2020,** *17* (3), 261–272.
7. Salerno, D.; Brogioli, D.; Cassina, V.; Turchi, D.; Beretta, G. L.; Seruggia, D.; Ziano, R.; Zunino, F.; Mantegazza, F. Magnetic tweezers measurements of the nanomechanical properties of DNA in the presence of drugs. *Nucleic acids research* **2010,** *38* (20), 7089–7099.
8. Dikic, J.; Seidel, R. Anticooperative Binding Governs the Mechanics of Ethidium-Complexed DNA. *Biophysical journal* **2019,** *116* (8), 1394–1405.
9. Kolbeck, P. J.; Tišma, M.; Analikwu, B. T.; Vanderlinden, W.; Dekker, C.; Lipfert, J. Supercoiling-dependent DNA binding: quantitative modeling and applications to bulk and single-molecule experiments. *Nucleic acids research* **2024,** *52* (1), 59–72.

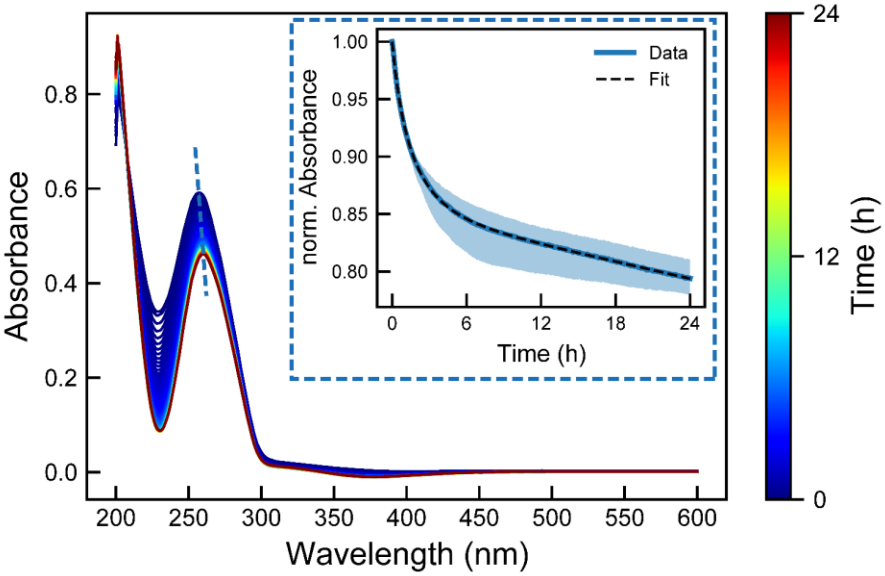

Figure S 1. Series of Absorption Spectra of λ-DNA incubated with $[PdCl_4]^{2-}$. $[PdCl_4]^{2-}$ spectra were subtracted to obtain the DNA spectra. The inset displays the absorbance value for the maximum of the DNA peak over time, normalized by the first data point. The solid line represents the mean of three repeated measurements and the colored area indicates the minimum and maximum observed value. The dashed line shows a fit to the mean using formula (4) from Supplementary Note 1.

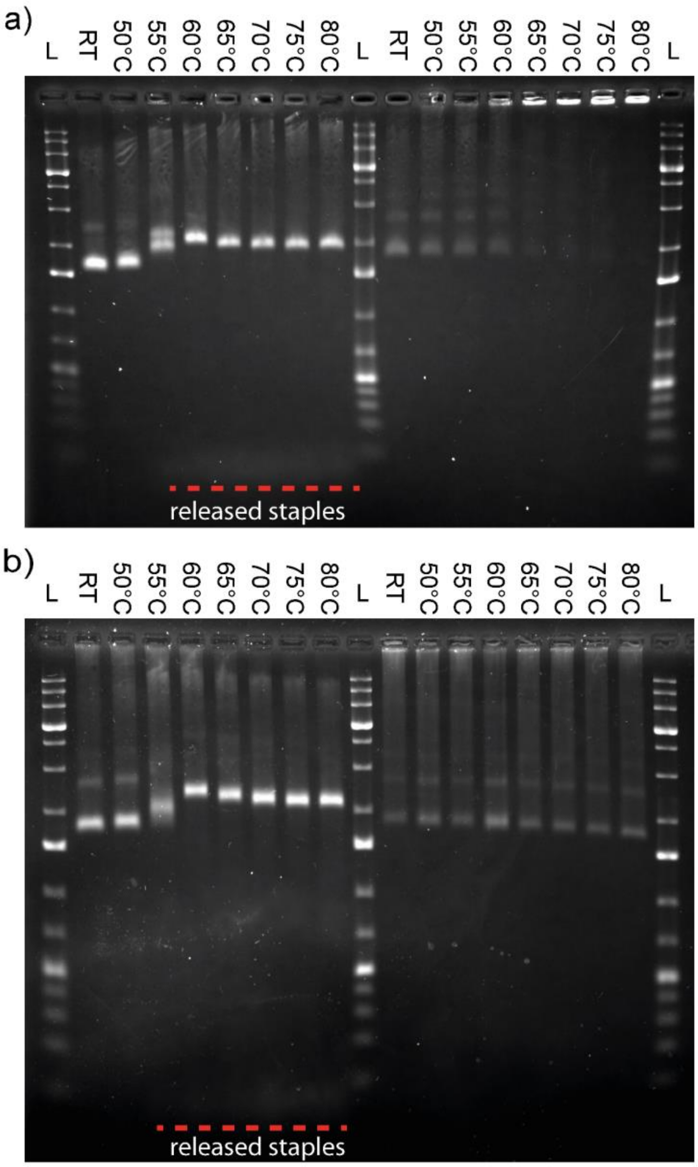

Figure S 2. Agarose gels of nanotubes at different temperatures before (left) and after (right) Pd stabilization at the bp:Pd ion ratios a) 1:20 and b) 1:1. The temperatures are denoted above the gels and L indicates a GeneRuler 1 Kb Plus DNA Ladder (Life Technologies GmbH). Untreated nanotubes disassemble at 55 °C indicated by an upwards shift of the nanotube bands and released staples, whereas $[PdCl_4]^{2-}$ treated nanotubes are stabilized over the entire temperature range.

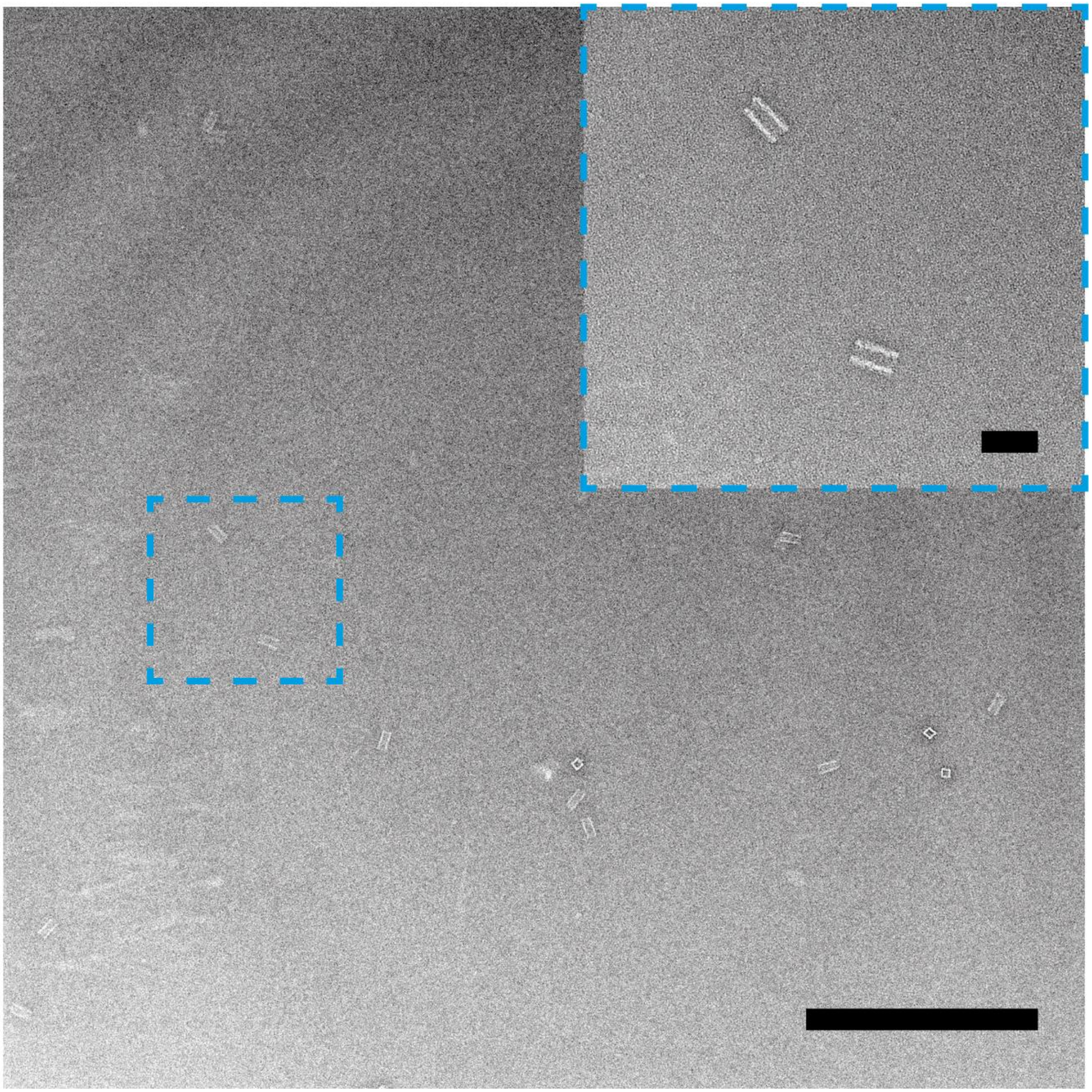

Figure S 3. Overview TEM image of nanotubes at RT. Scale bar of the overview image equal 500 nm and of the inset 50 nm.

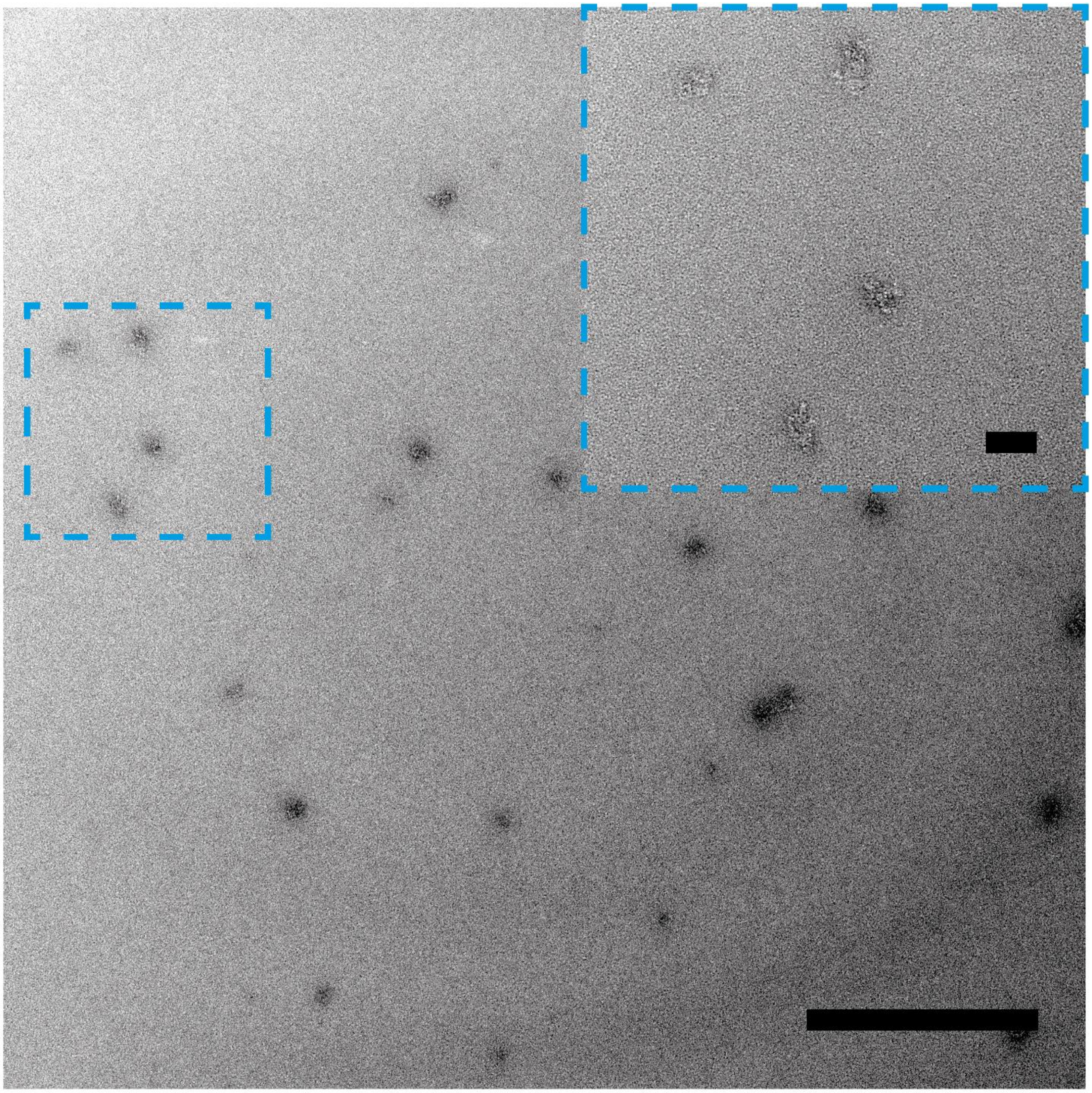

Figure S 4. Overview TEM image of nanotubes at 65 °C. Scale bar of the overview image equal 500 nm and of the inset 50 nm.

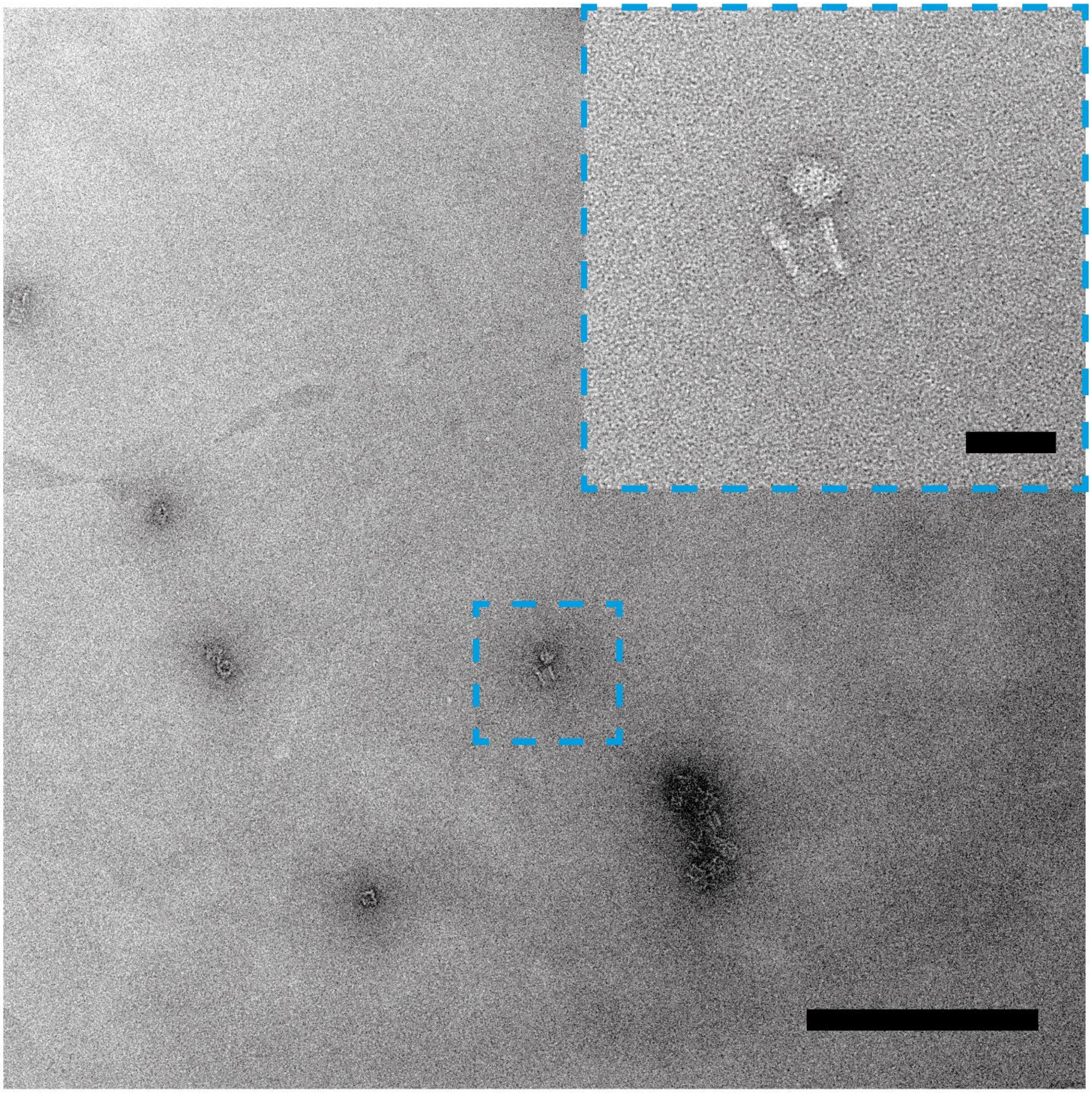

Figure S 5. Overview TEM image of nanotubes stabilized with $[PdCl_4]^{2-}$ at a 2:1 ratio (bp:Pd ions) at RT. Scale bar of the overview image equal 500 nm and of the inset 50 nm.

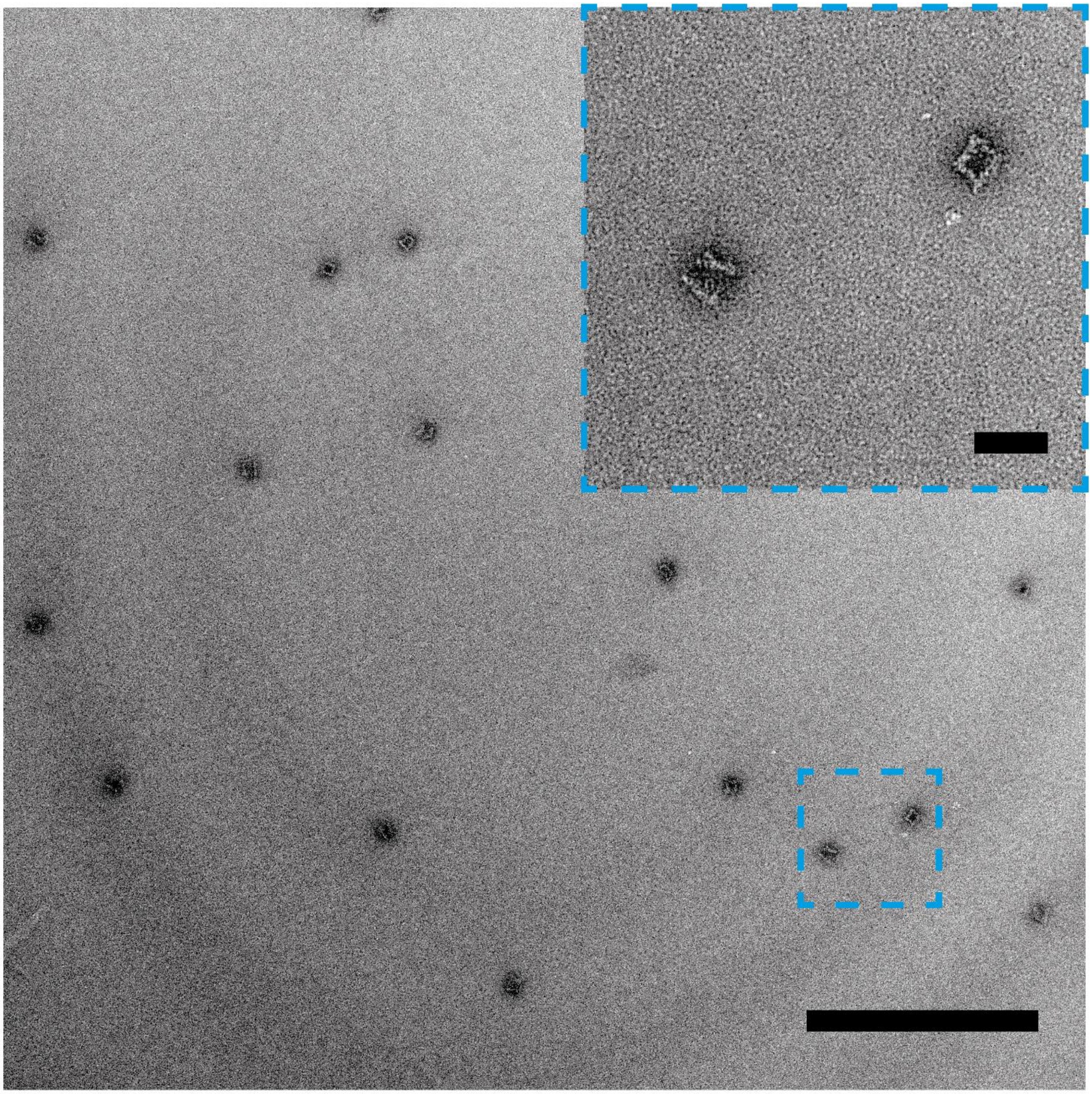

Figure S 6. Overview TEM image of nanotubes stabilized with $[PdCl_4]^{2-}$ at a 2:1 ratio (bp:Pd ions) at 100 °C. Scale bar of the overview image equal 500 nm and of the inset 50 nm.

Table S 1. Nanotube dimensions based on TEM measurements.

	Length (nm)	Width (nm)	N
w/o Pd, RT	41 ± 2	20 ± 1	26
w/ Pd, RT	30 ± 5	25 ± 2	26
w/ Pd, 65 °C	29 ± 3	26 ± 3	28
w/ Pd, 80 °C	25 ± 3	25 ± 3	28
w/ Pd, 95 °C	24 ± 2	25 ± 3	32
w/ Pd, 100 °C	24 ± 3	25 ± 3	54

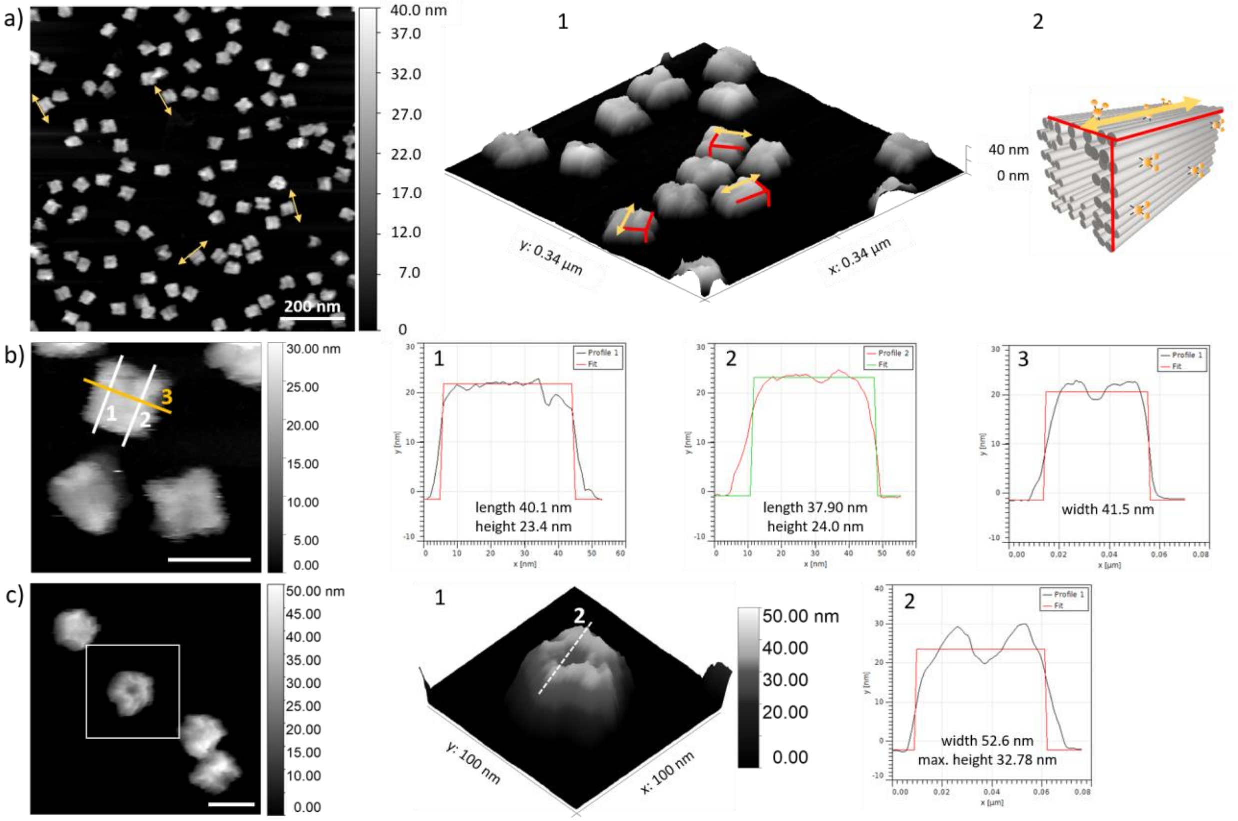

Figure S 7. AFM analysis of DNA origami nanotubes. a) AFM image of $[PdCl4]^{2-}$ stabilized DNA nanotubes using a ratio of 2:1 (bp:Pd ions) at RT. Arrows (yellow) mark the orientation of the sidewalls. Panel 1 shows a 3D view of a part of the AFM image with orientation markers (yellow) and a selection of the side walls of the nanotubes (red). Panel 2 is a schematic representation for the orientation, selection, and assignment of the local structural dimensions. b) AFM image of DNA origami nanotubes adsorbed with a sidewall to the substrate. Panel 1 and 2 show the height profiles along the nanotube walls and panel 2 the height profile in perpendicular direction across the nanotube cavity seen as a dip in the profile. c) AFM image of a standing DNA origami nanotube. Panel 1 shows a 3D view and panel 2 the corresponding height profile.

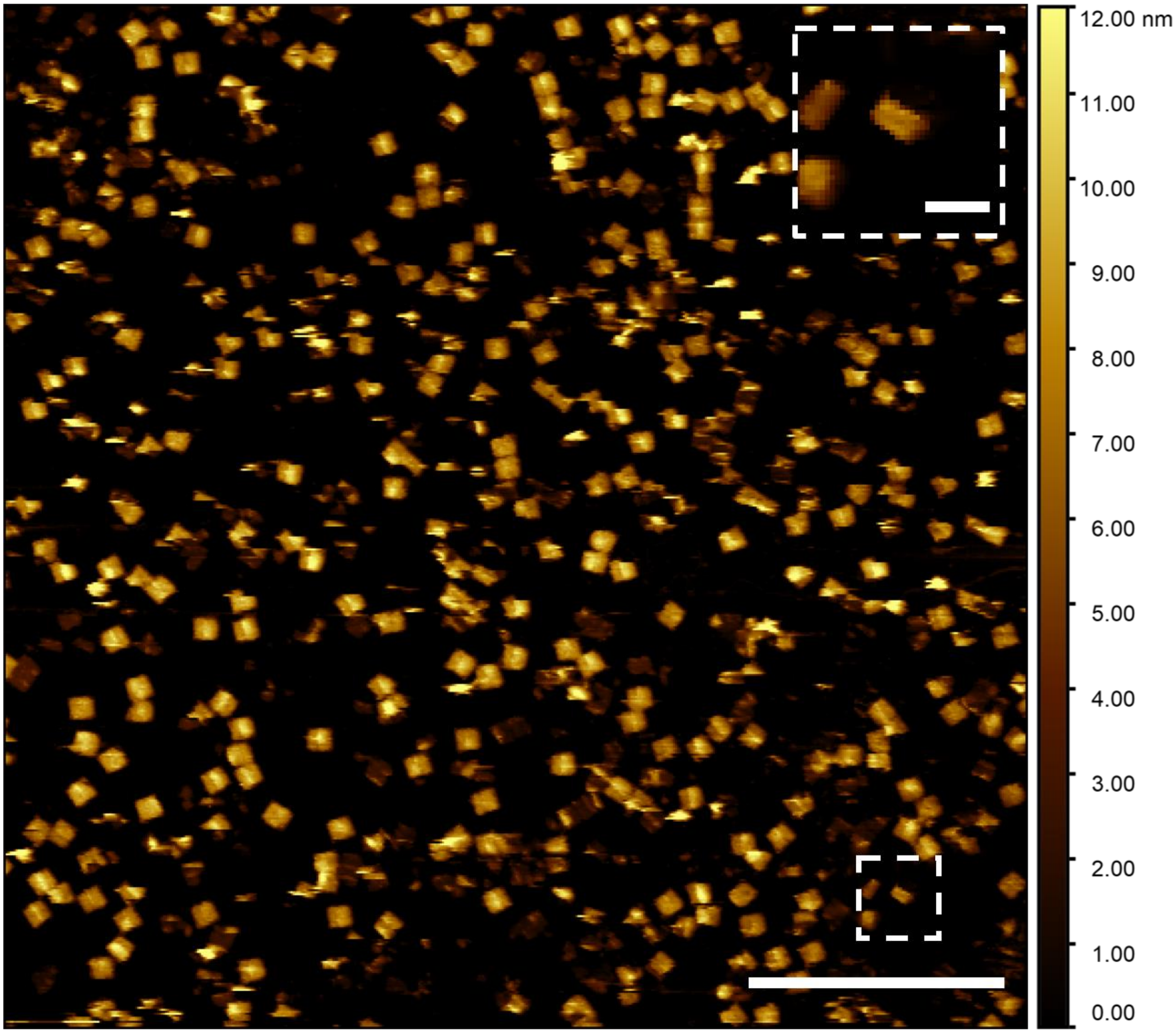

Figure S 8. Overview AFM image in liquid of nanotubes at RT. Scale bar of the overview image equal 500 nm and of the inset 50 nm.

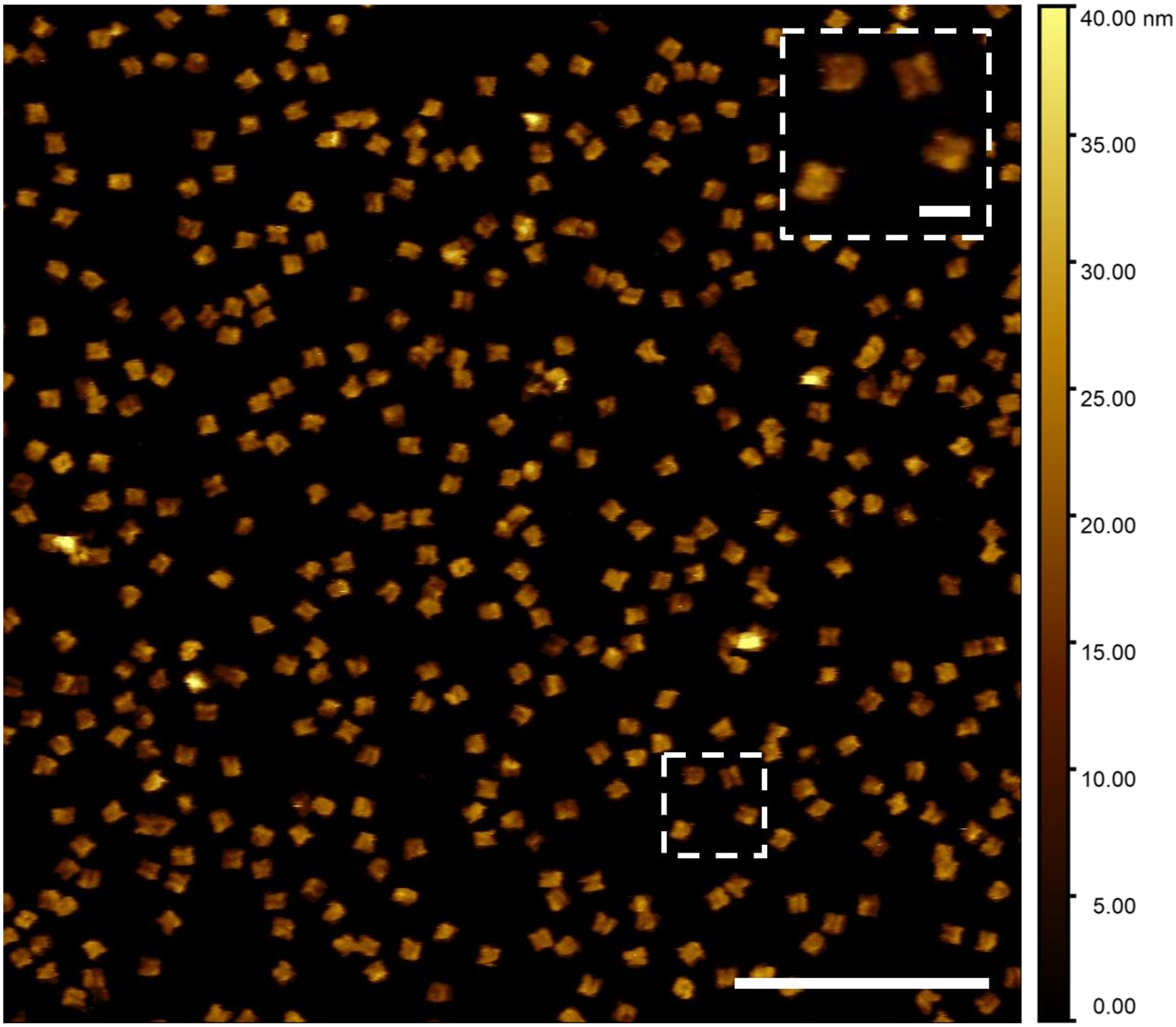

Figure S 9. Overview AFM image in liquid of nanotubes stabilized with $[PdCl_4]^{2-}$ at a 2:1 ratio (bp:Pd ions) at RT. Scale bar of the overview image equals to 500 nm and of the inset 50 nm.

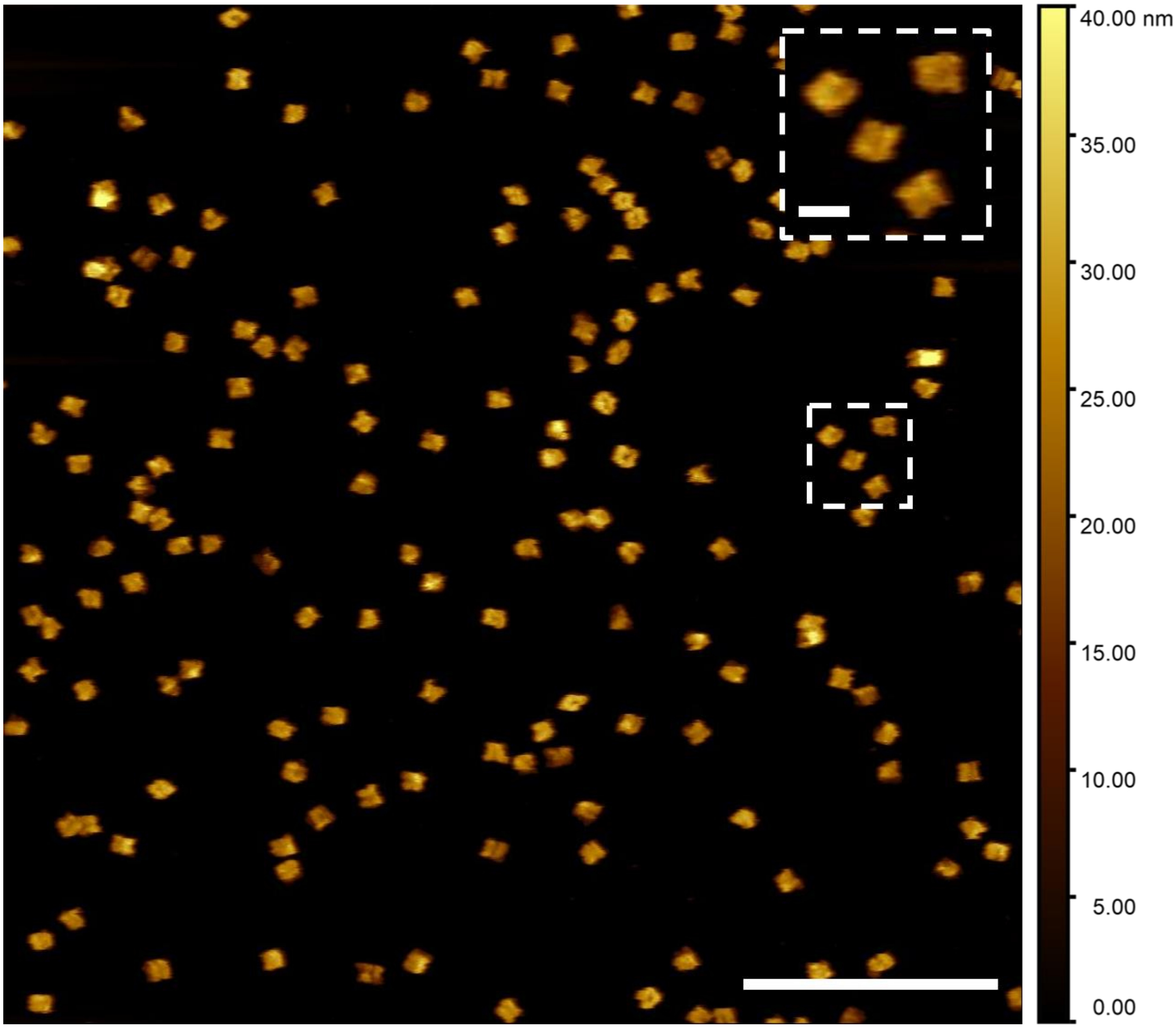

Figure S 10. Overview AFM image in liquid of nanotubes stabilized with $[PdCl_4]^{2-}$ at a 2:1 ratio (bp:Pd ions) at 65 °C. Scale bar of the overview image equal 500 nm and of the inset 50 nm.

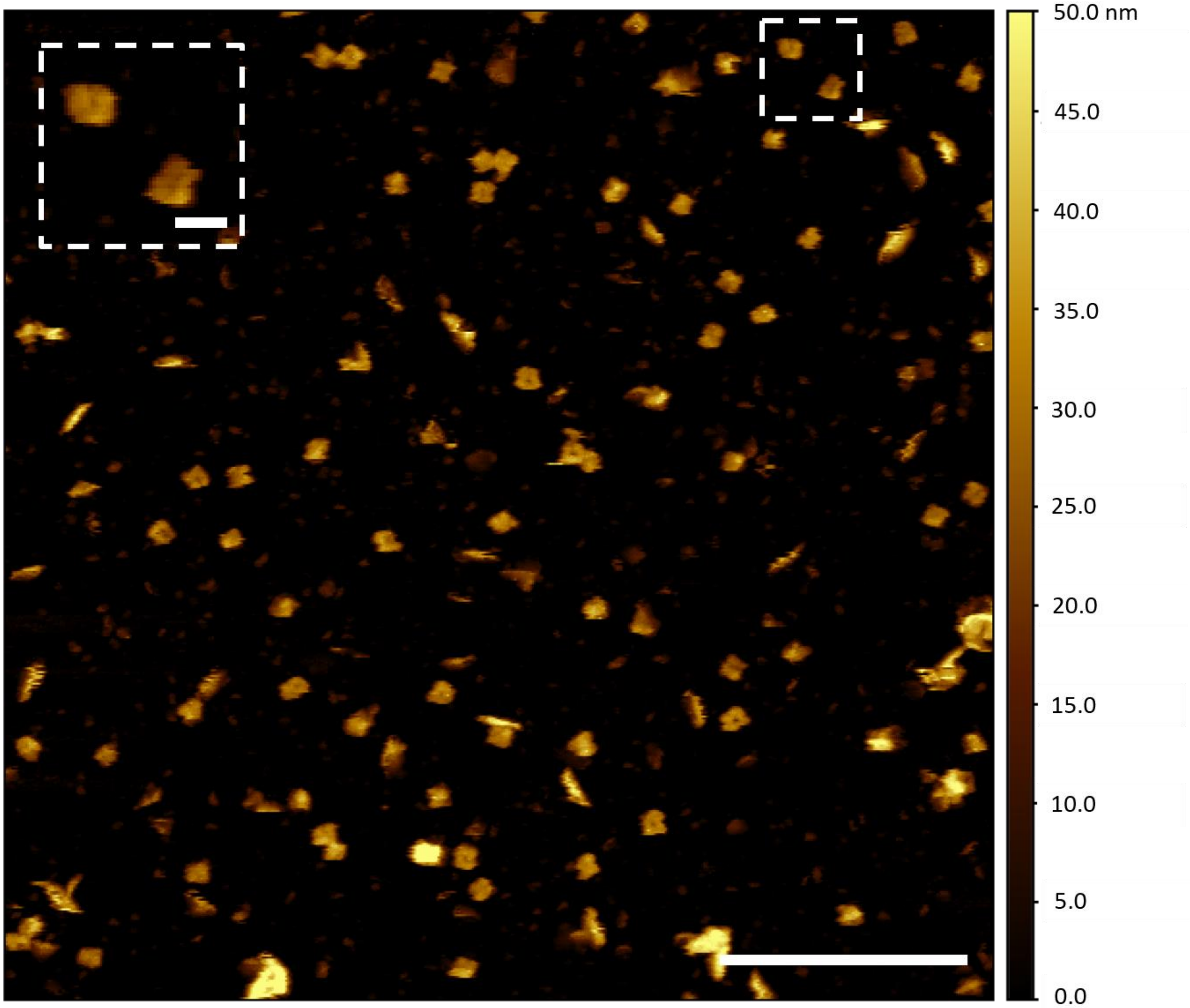

Figure S 11. Overview AFM image in liquid of nanotubes stabilized with $[PdCl_4]^{2-}$ at a 2:1 ratio (bp:Pd ions) at 80 °C. Scale bar of the overview image equal 500 nm and of the inset 50 nm. The height scale equals 40 nm.

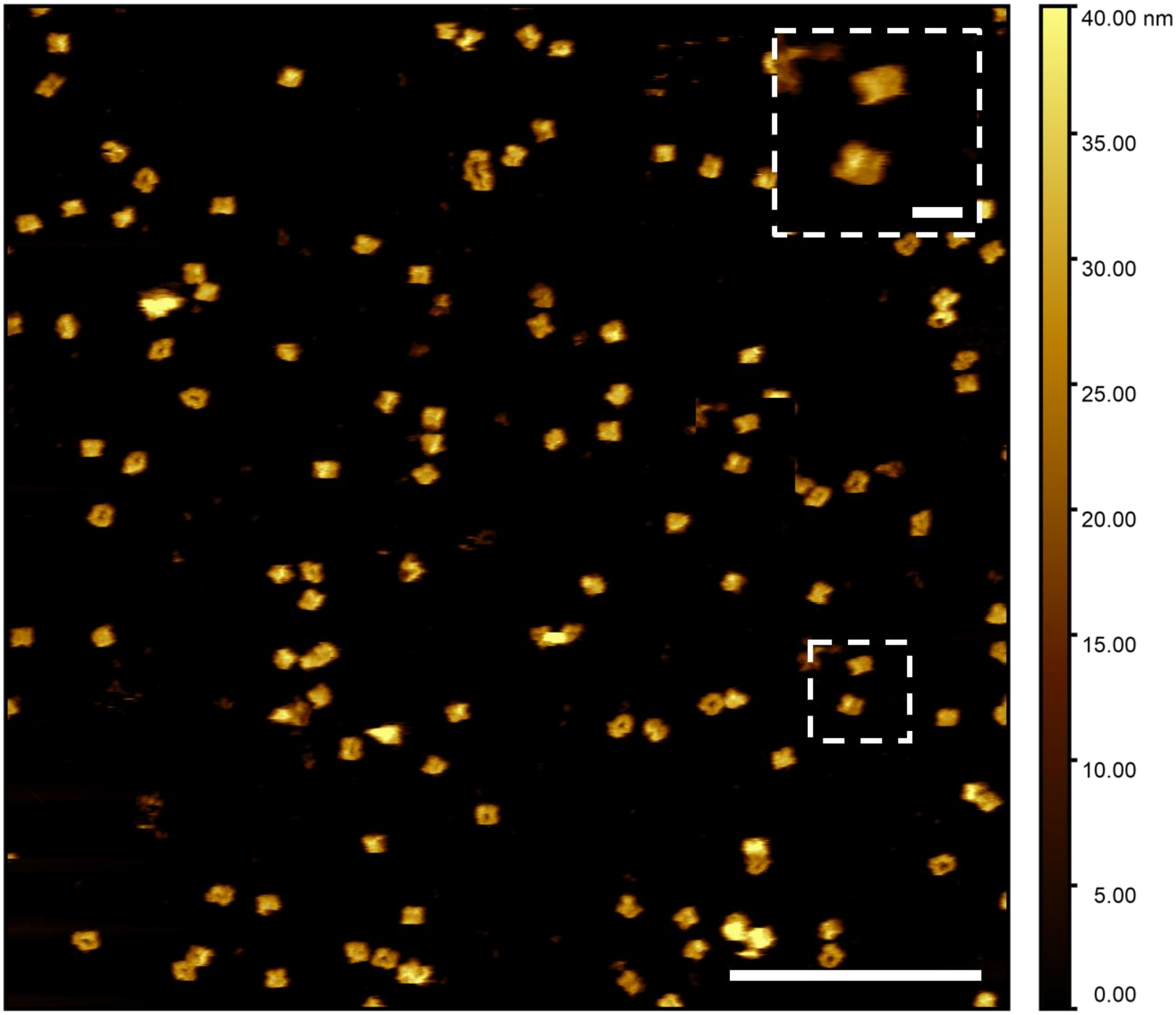

Figure S 12. Overview AFM image in liquid of nanotubes stabilized with $[PdCl_4]^{2-}$ at a 2:1 ratio (bp:Pd ions) at 95 °C. Scale bar of the overview image equal 500 nm and of the inset 50 nm.

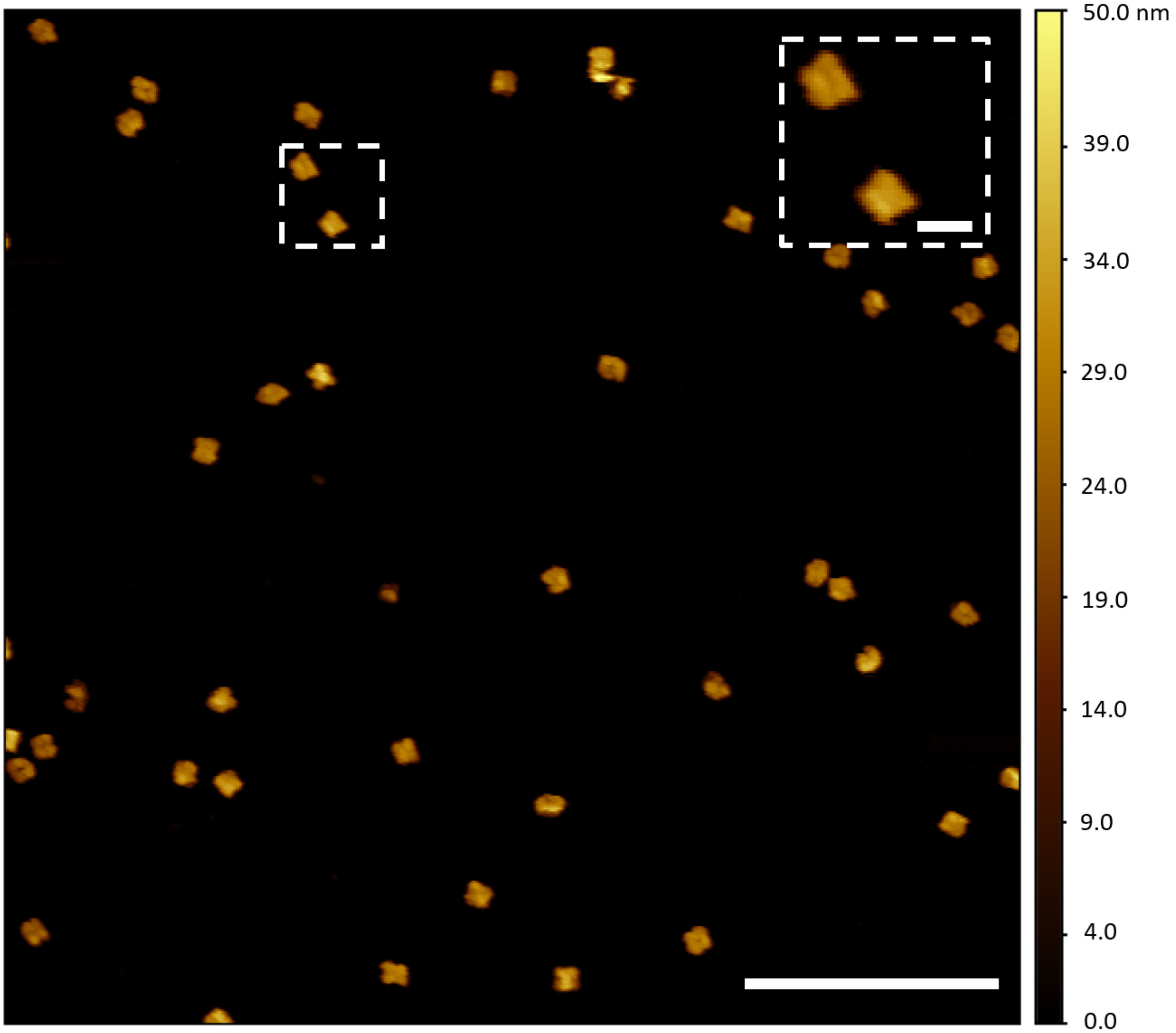

Figure S 13. Overview AFM image in liquid of nanotubes stabilized with $[PdCl_4]^{2-}$ at a 2:1 ratio (bp:Pd ions) at 100 °C. Scale bar of the overview image equal 500 nm and of the inset 50 nm.

Table S 2. Nanotube dimensions based on AFM measurements in liquid. We attribute the increase in length and width in comparison to the TEM samples to the tip convolution.

	Length (nm)	Width (nm)	Height (nm)	N
w/o Pd, RT	42 ± 3	23 ± 3	9 ± 1	30
w/ Pd, RT	42 ± 4	42 ± 3	24 ± 1	30
w/ Pd, 65 °C	43 ± 4	44 ± 5	26 ± 2	30
w/ Pd, 80 °C	45 ± 6	45 ± 5	30 ± 3	30
w/ Pd, 95 °C	45 ± 3	44 ± 3	30 ± 2	30
w/ Pd, 100 °C	47 ± 6	45 ± 4	31 ± 2	30

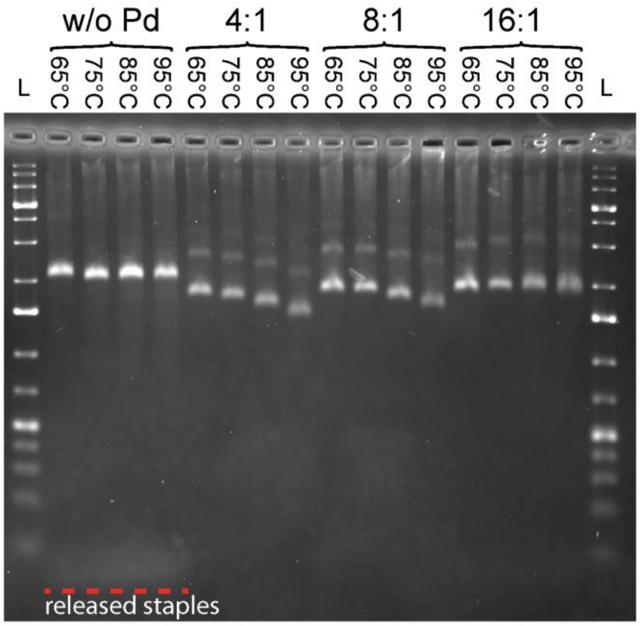

Figure S 14. Agarose gel showing the influence of $[PdCl_4]^{2-}$ on the thermal stability of nanotubes at different bp:Pd ion ratios. The temperatures and ratios are denoted above the gel and L indicates a GeneRuler 1 Kb Plus DNA Ladder (Life Technologies GmbH). All $[PdCl_4]^{2-}$ treated nanotubes display no released staple strands, and the 4:1 and 8:1 samples show a downshift in band height for increasing temperature, that was observed for stabilized structures at a 2:1 ratio.

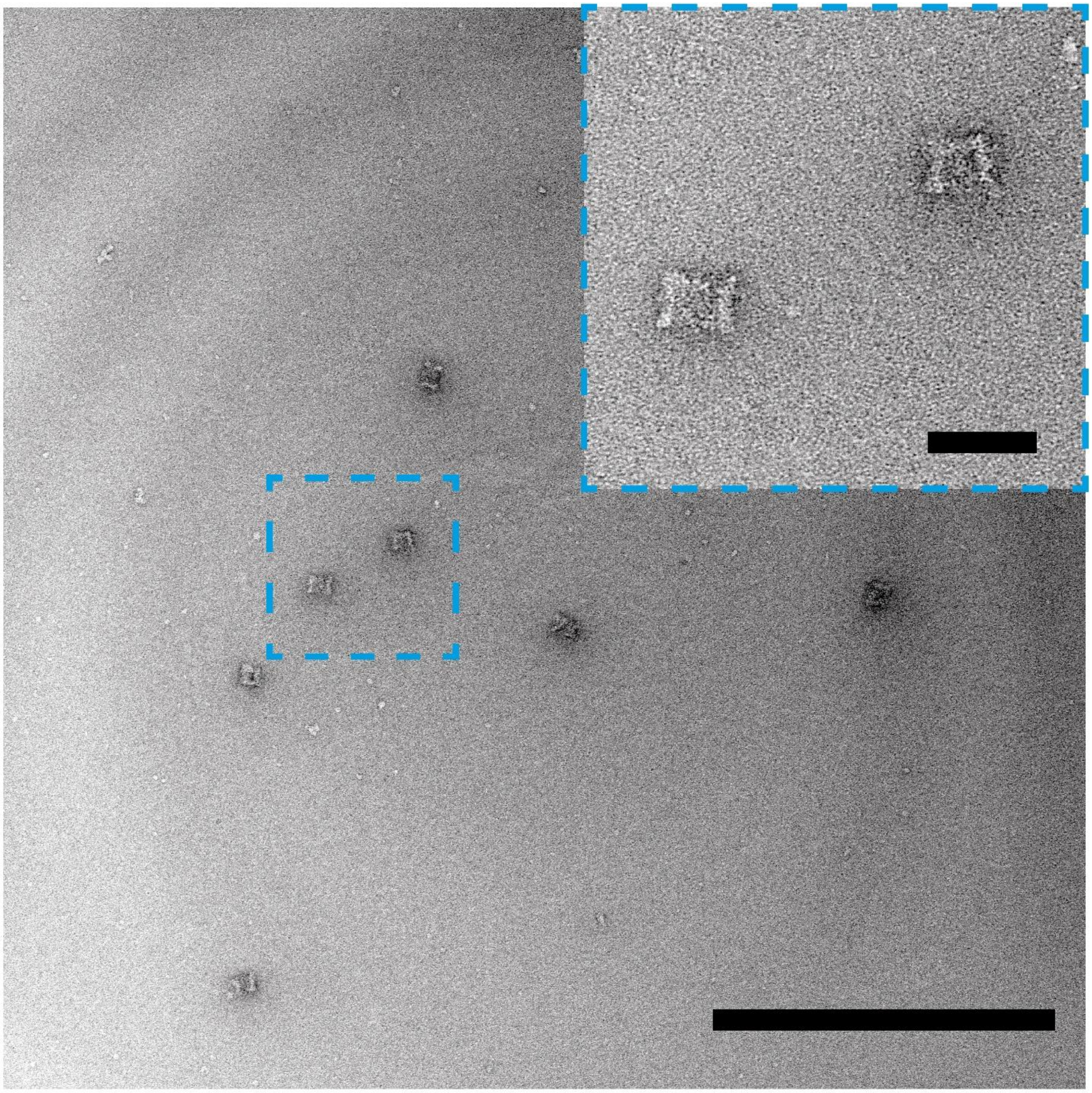

Figure S 15. Overview TEM image of nanotubes stabilized with $[PdCl_4]^{2-}$ at a 4:1 ratio (bp:Pd ions) at 95 °C. Scale bar of the overview image equal 500 nm and of the inset 50 nm.

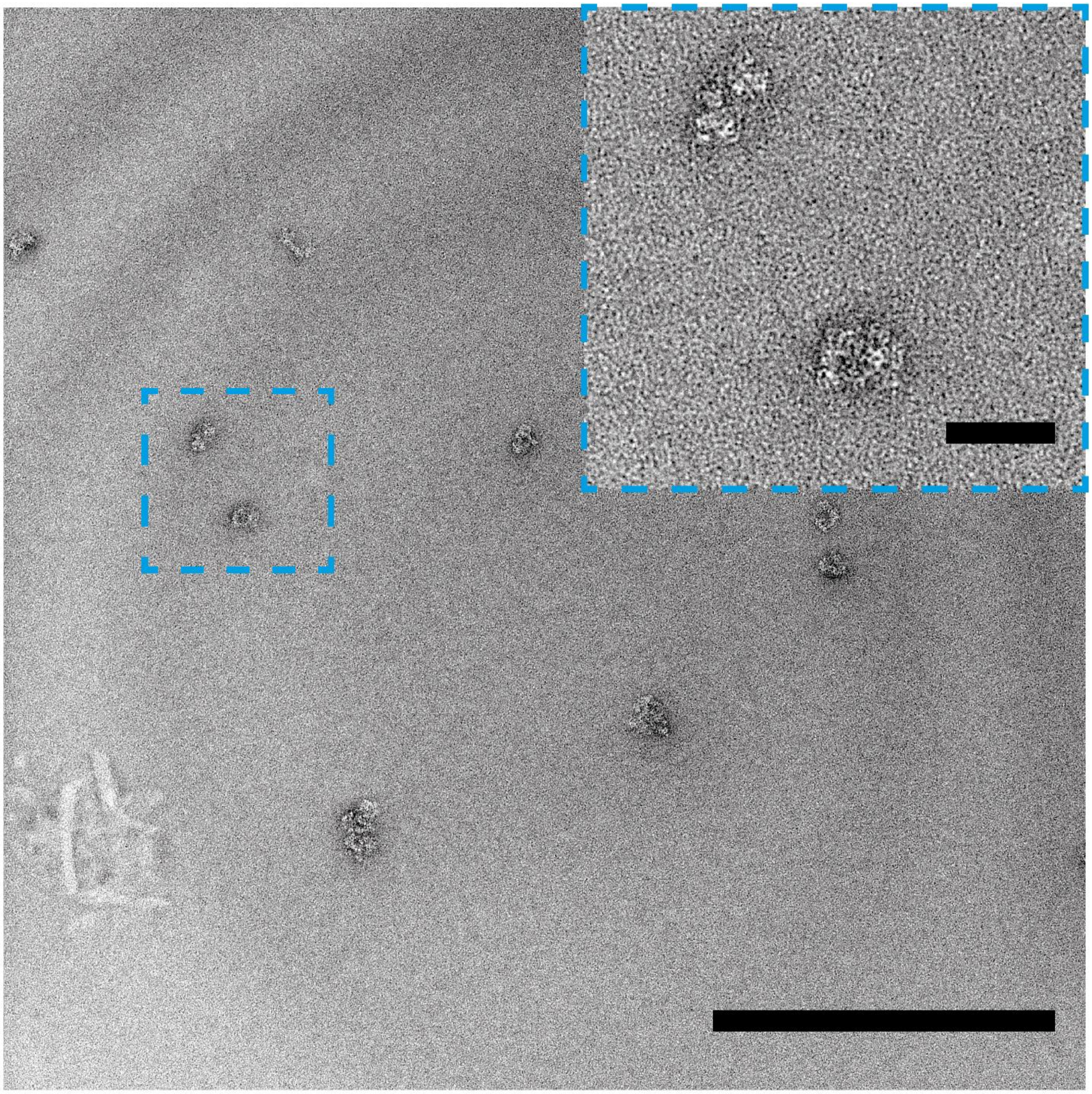

Figure S 16. Overview TEM image of nanotubes stabilized with $[PdCl_4]^{2-}$ at a 8:1 ratio (bp:Pd ions) at 95 °C. Scale bar of the overview image equal 500 nm and of the inset 50 nm.

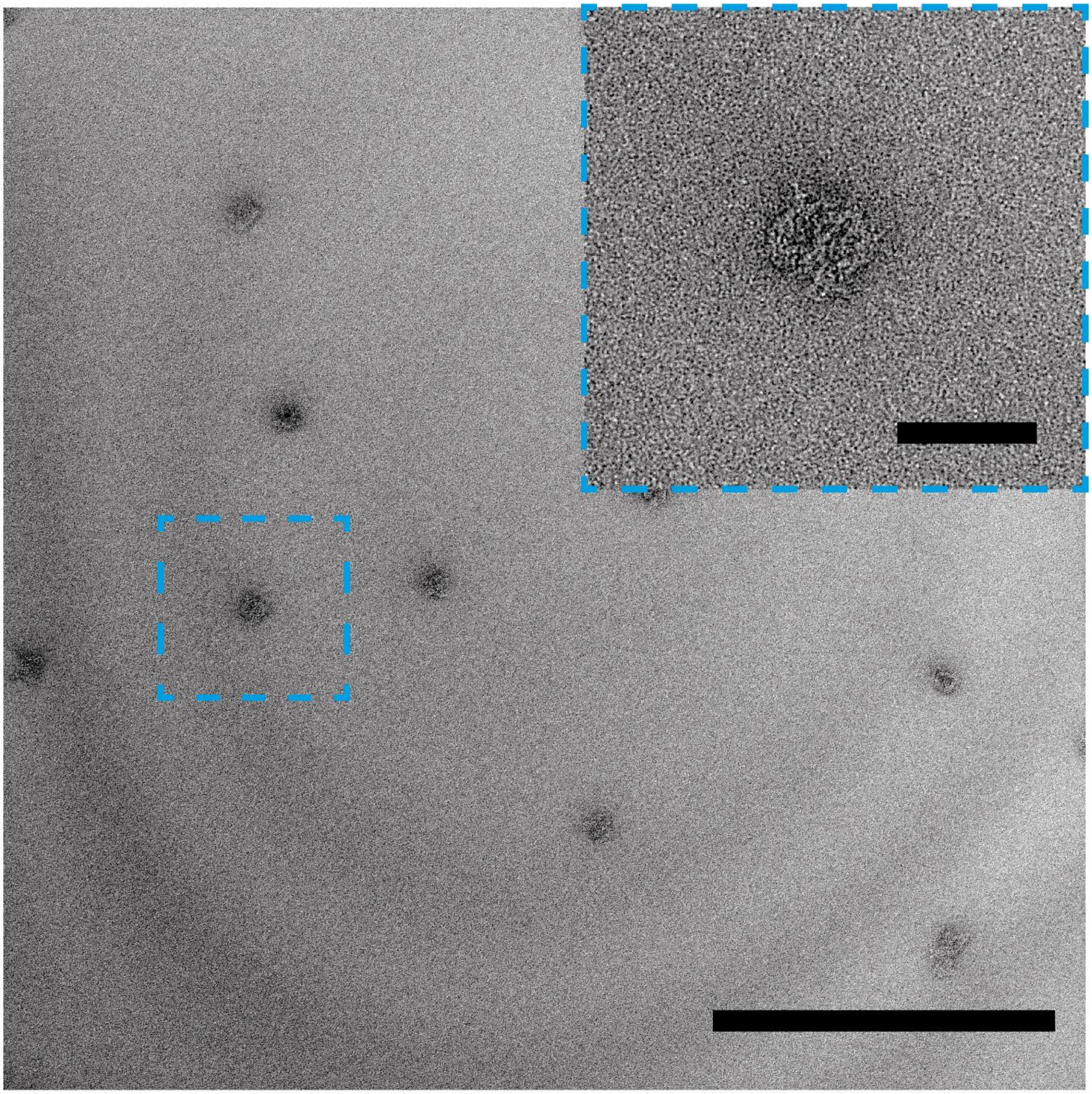

Figure S 17. Overview TEM image of nanotubes stabilized with $[PdCl_4]^{2-}$ at a 16:1 ratio (bp:Pd ions) at 95 °C. Scale bar of the overview image equal 500 nm and of the inset 50 nm.

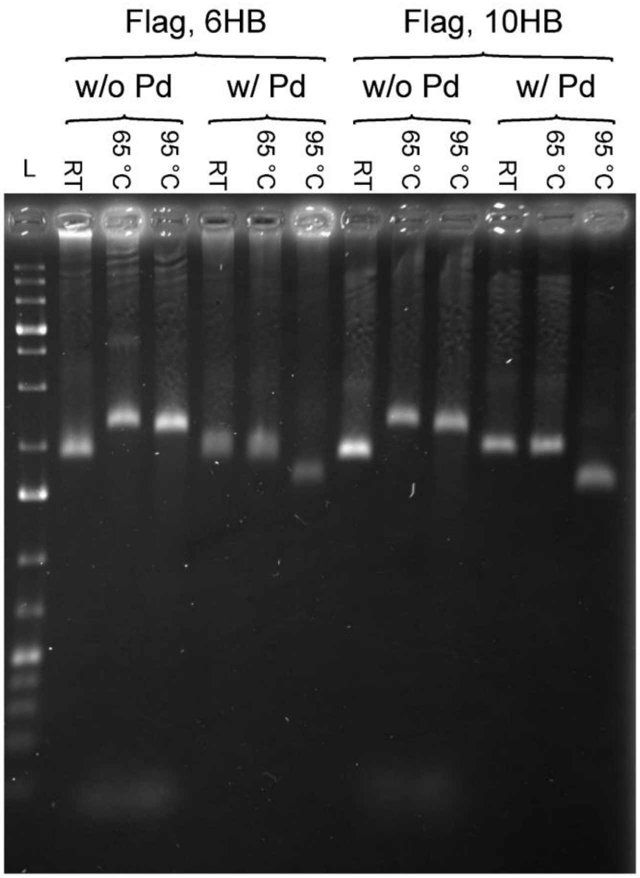

Figure S 18. Agarose gels showing the influence of $[PdCl_4]^{2-}$ on the thermal stability of DNA origami flags at a 2:1 ratio (bp:Pd ions) at different temperatures. The temperatures and different pole types are denoted above the gel and L indicates a GeneRuler 1 Kb Plus DNA Ladder (Life Technologies GmbH). Untreated samples show an upwards shift in band height as well as a release of staple strands at elevated temperatures. In contrast Pd stabilized samples display the same downwards shift as observed for nanotubes.

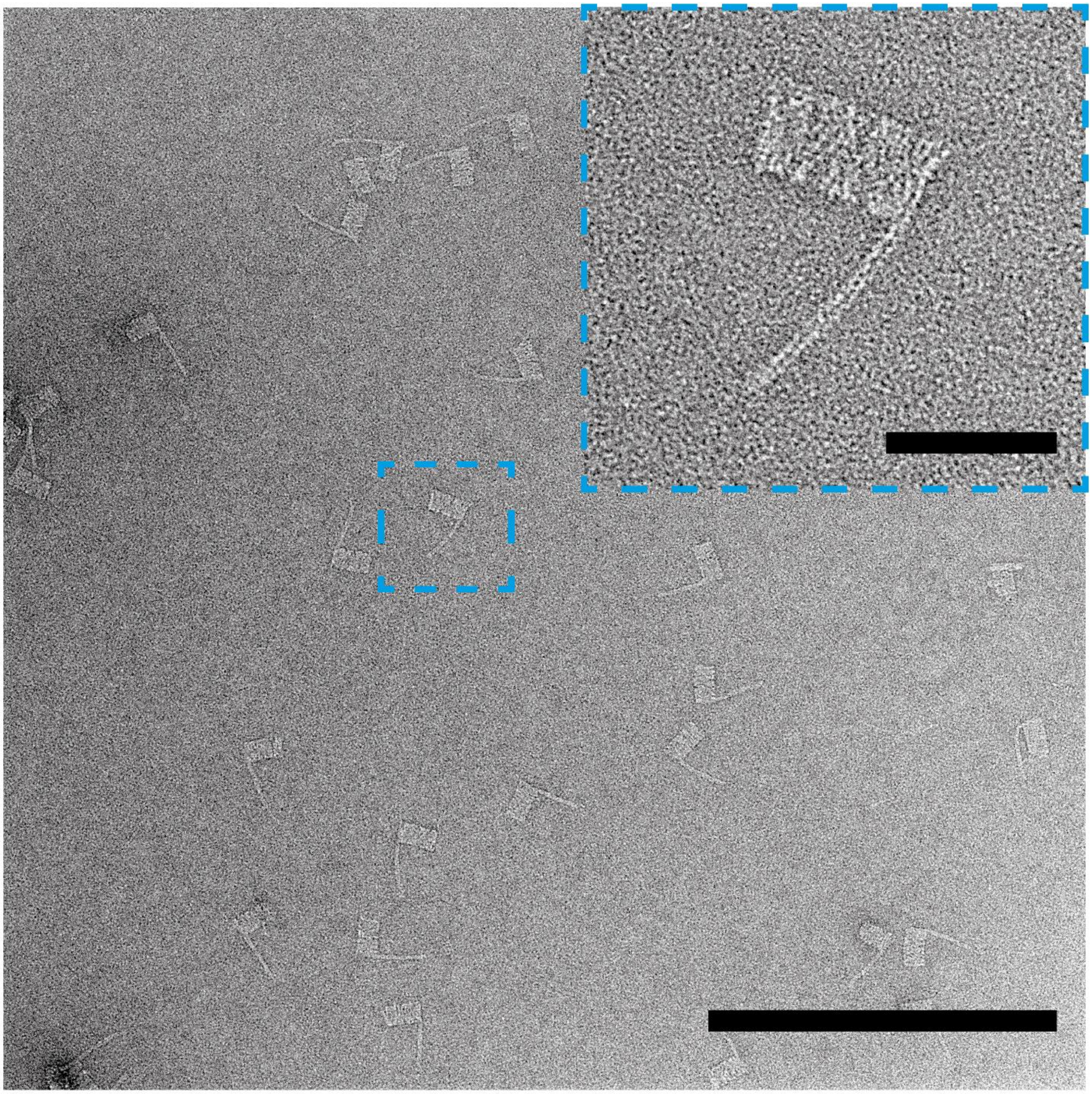

Figure S 19. Overview TEM image of DNA origami flags with a 6HB pole at RT. Scale bar of the overview image equal 500 nm and of the inset 50 nm.

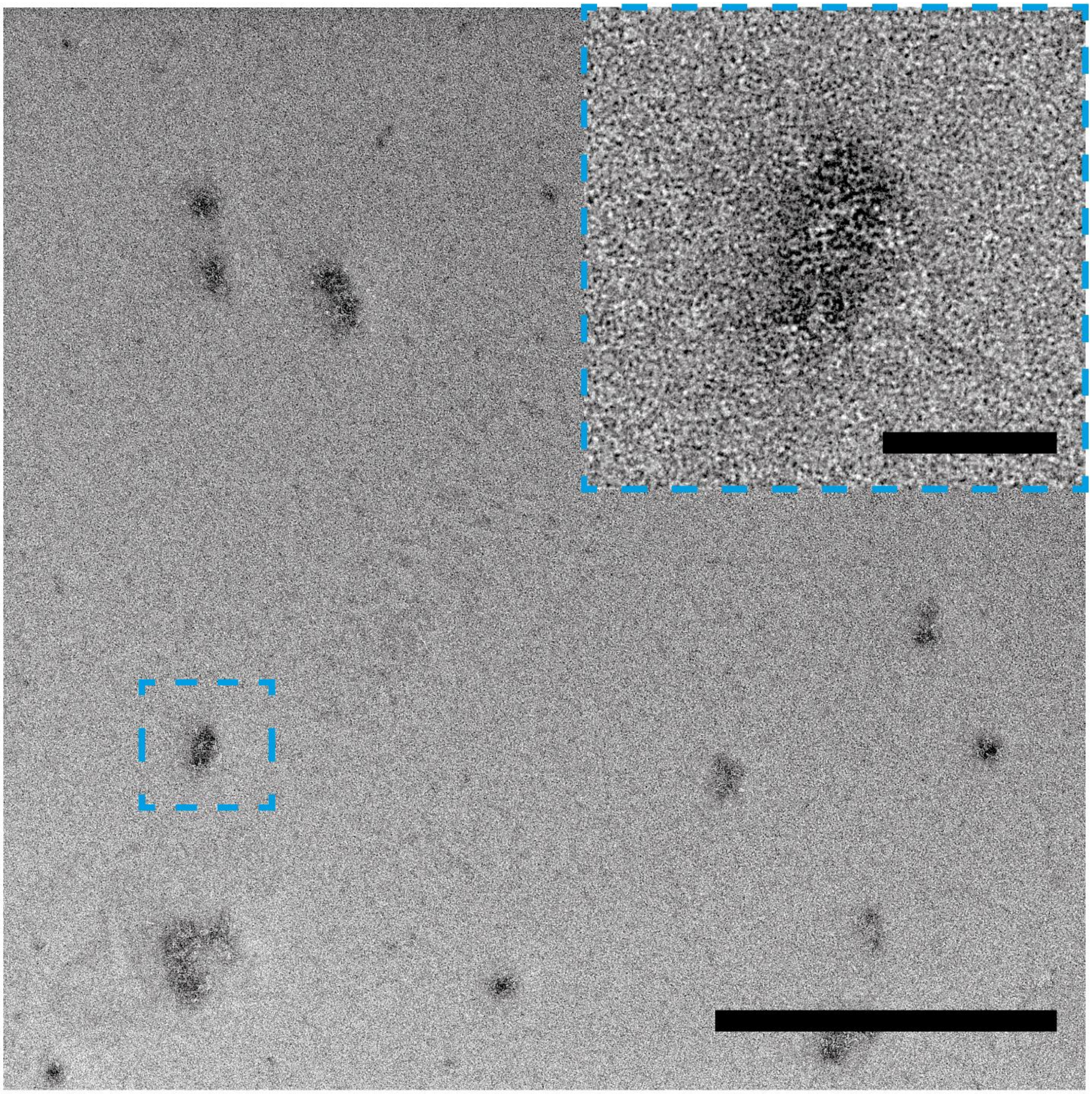

Figure S 20. Overview TEM image of DNA origami flags with a 6HB pole at 65 °C. Scale bar of the overview image equal 500 nm and of the inset 50 nm.

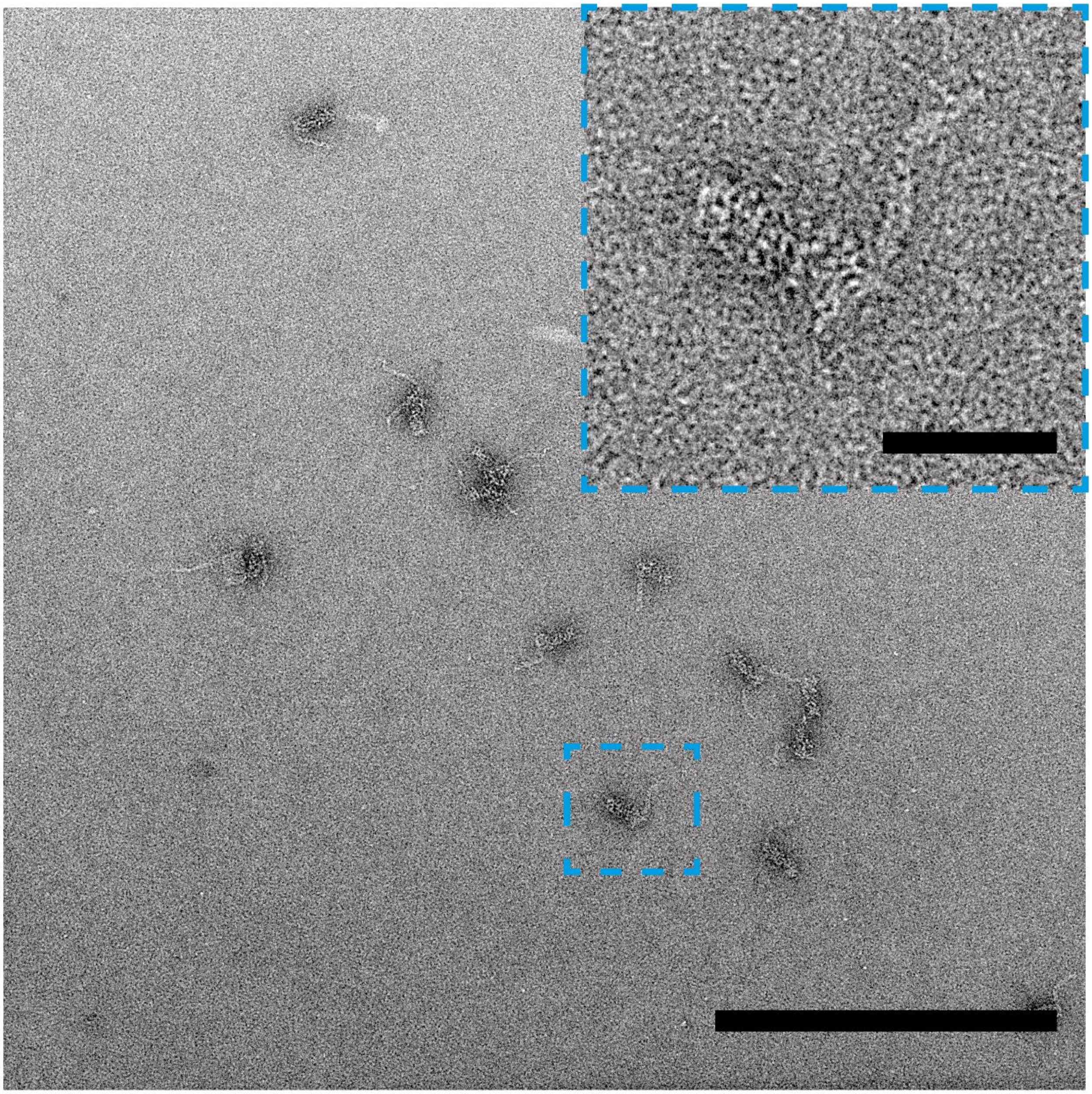

Figure S 21. Overview TEM image of DNA origami flags with a 6HB pole stabilized with $[PdCl_4]^{2-}$ at a 2:1 ratio (bp:Pd ions) at RT. Scale bar of the overview image equal 500 nm and of the inset 50 nm.

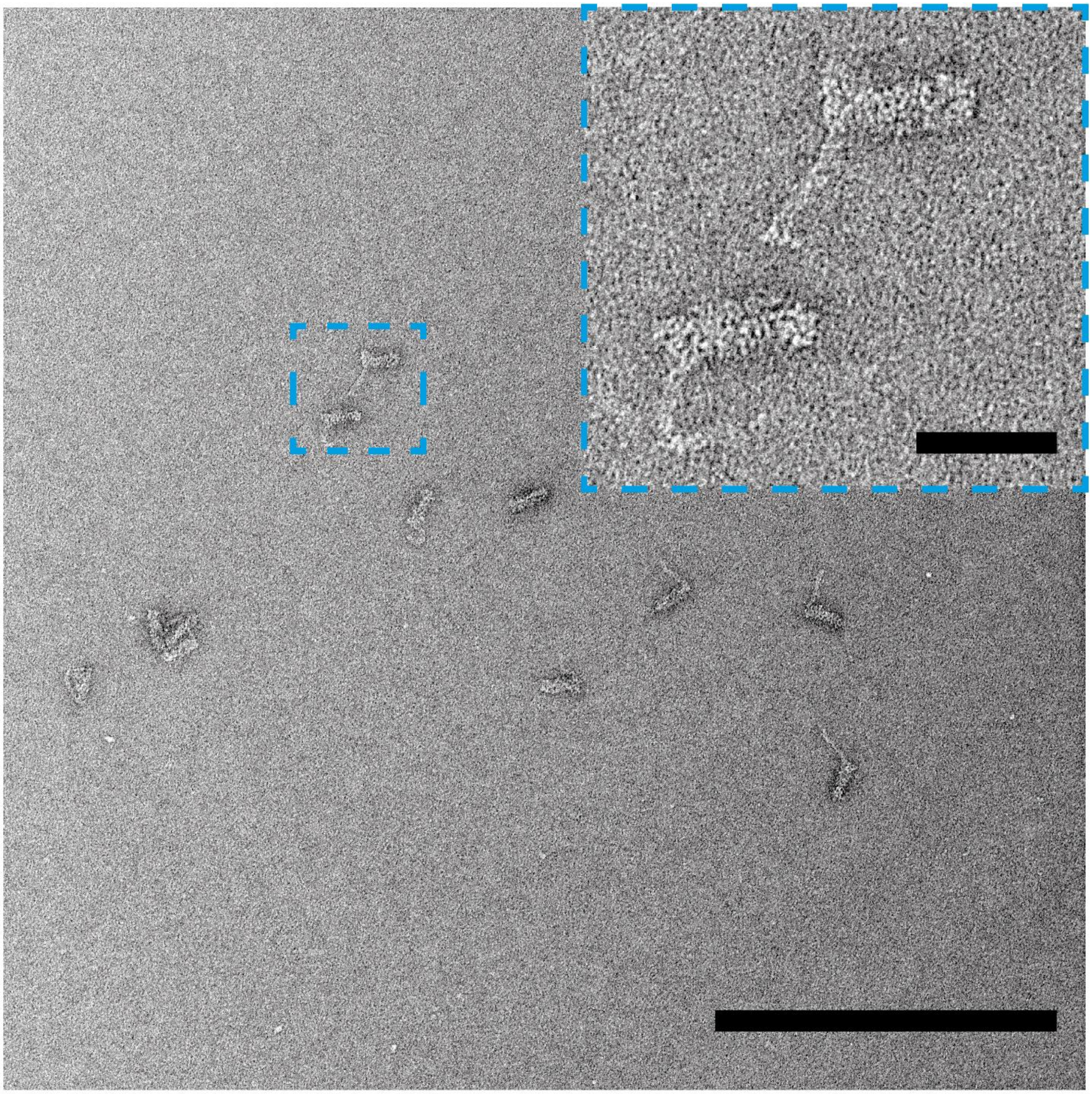

Figure S 22. Overview TEM image of DNA origami flags with a 6HB pole stabilized with $[PdCl_4]^{2-}$ at a 2:1 ratio (bp:Pd ions) at 95 °C. Scale bar of the overview image equal 500 nm and of the inset 50 nm.

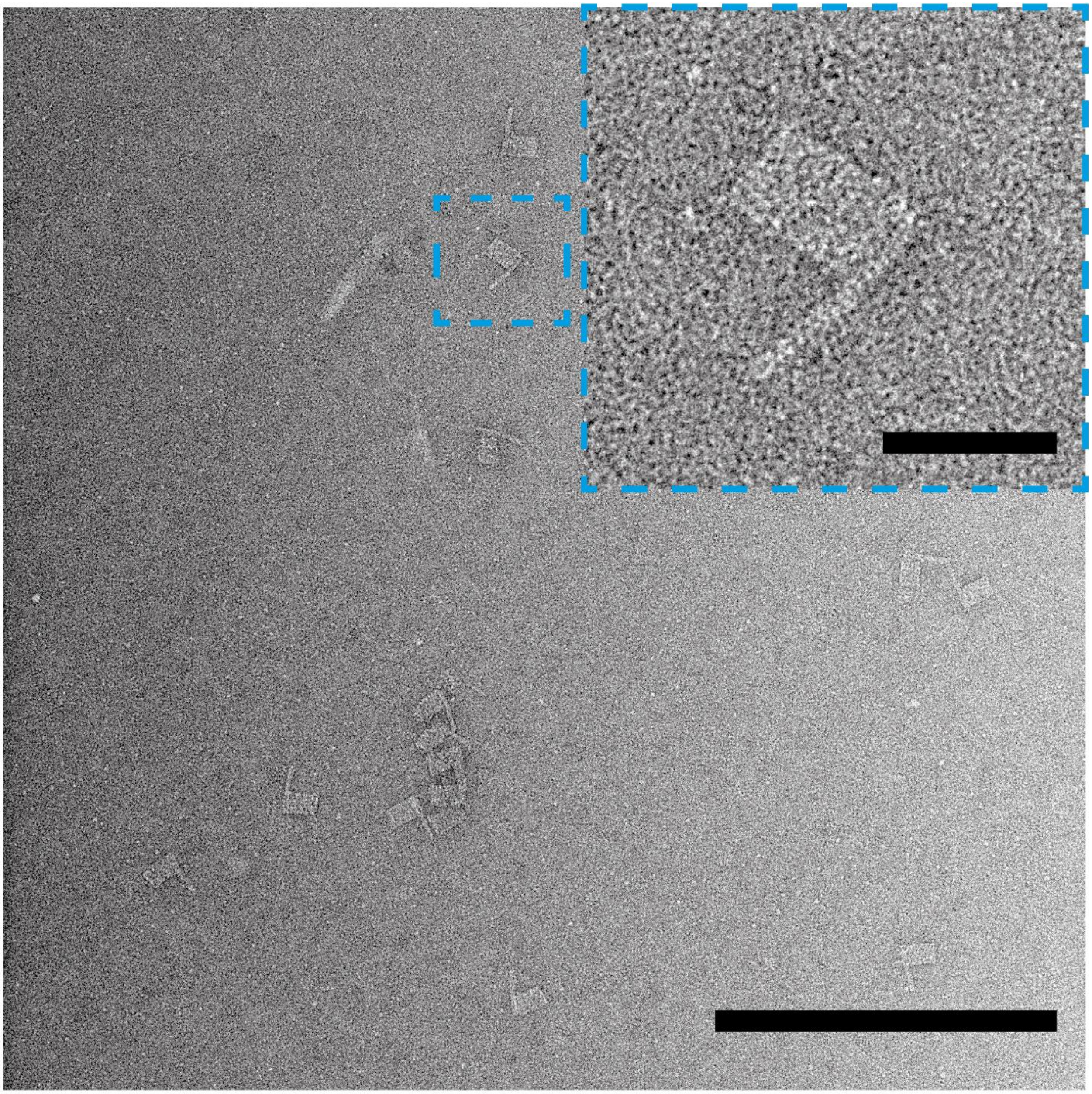

Figure S 23. Overview TEM image of DNA origami flags with a 10HB pole at RT. Scale bar of the overview image equal 500 nm and of the inset 50 nm.

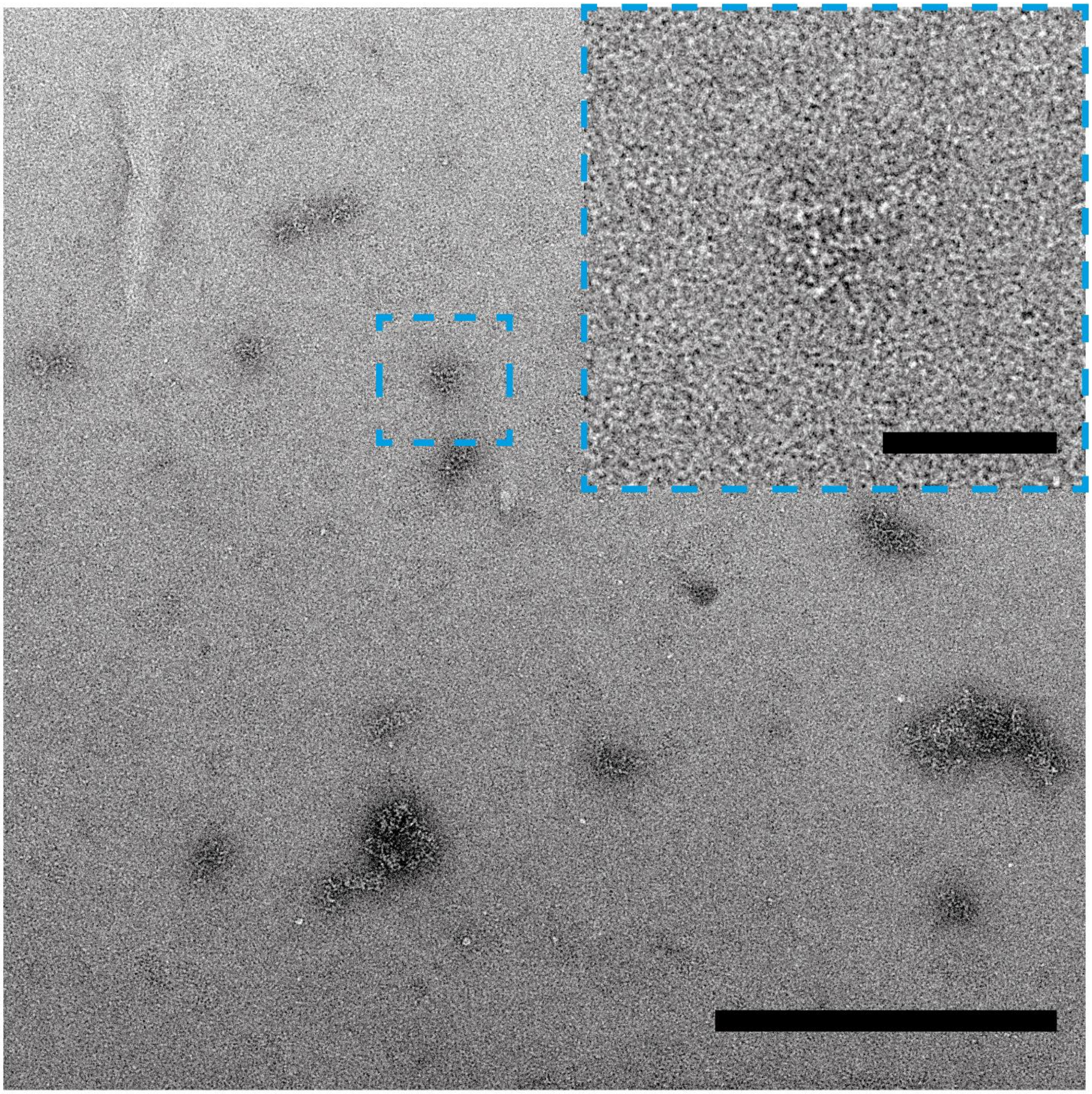

Figure S 24. Overview TEM image of DNA origami flags with a 10HB pole at 65 °C. Scale bar of the overview image equal 500 nm and of the inset 50 nm.

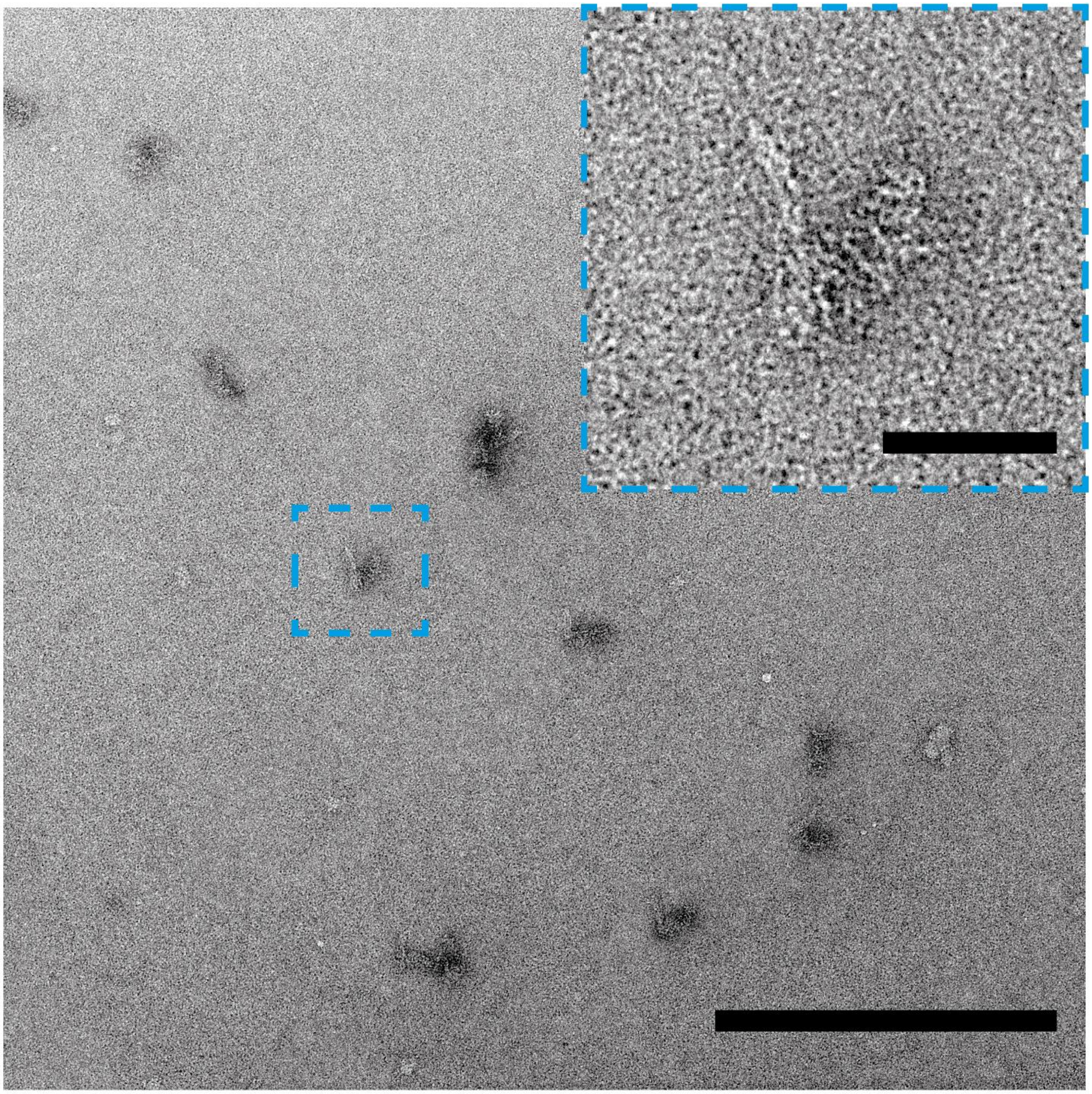

Figure S 25. Overview TEM image of DNA origami flags with a 10HB pole stabilized with $[PdCl_4]^{2-}$ at a 2:1 ratio (bp:Pd ions) at RT. Scale bar of the overview image equal 500 nm and of the inset 50 nm.

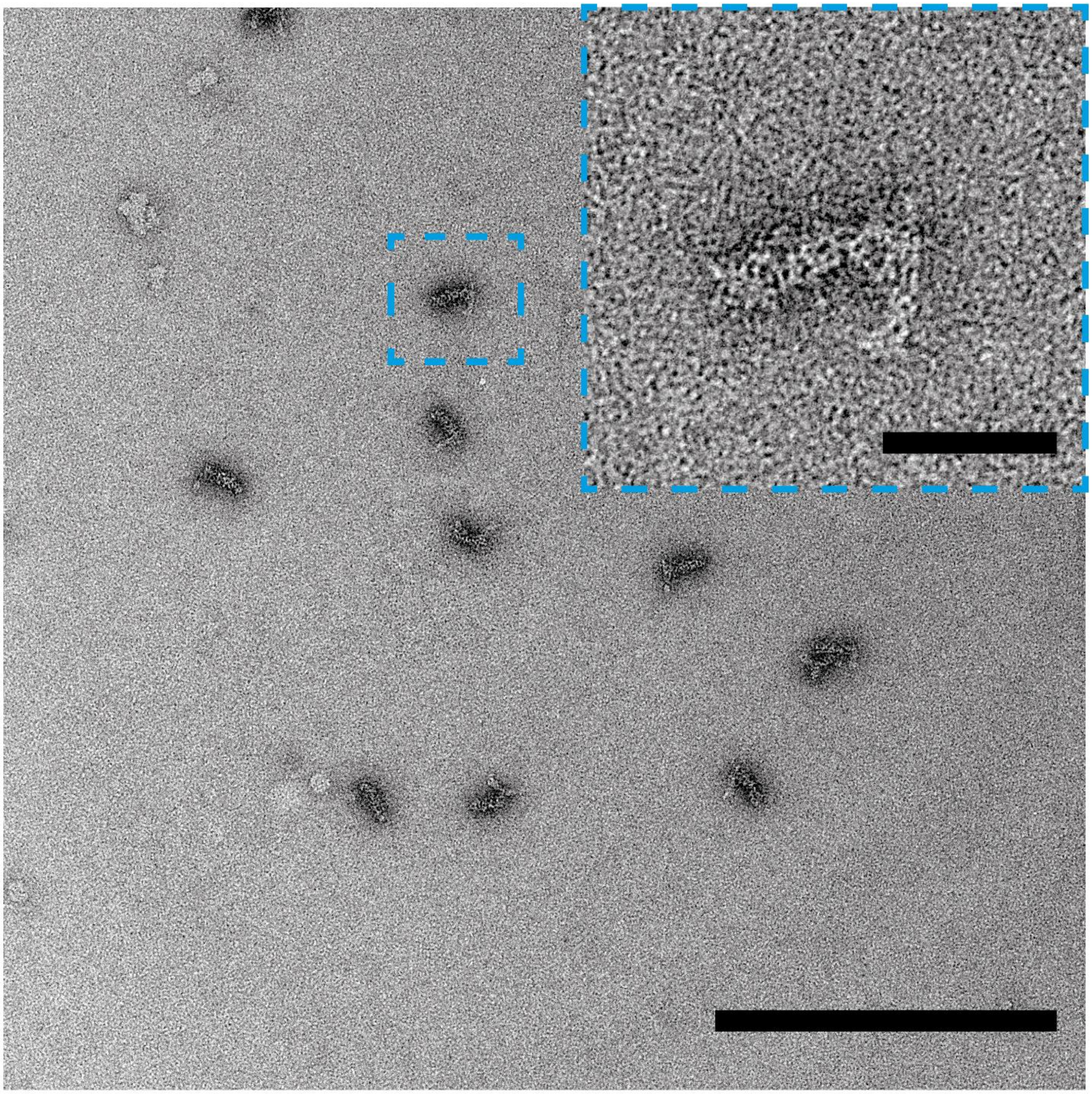

Figure S 26. Overview TEM image of DNA origami flags with a 10HB pole stabilized with $[PdCl_4]^{2-}$ at a 2:1 ratio (bp:Pd ions) at 95 °C. Scale bar of the overview image equal 500 nm and of the inset 50 nm.

Table S 3. DNA origami flag dimensions based on TEM measurements.

F6HB, Pole	Length (nm)	N
w/o Pd, RT	66 ± 8	39
w/ Pd, RT	57 ± 7	29
w/ Pd, 95 °C	47 ± 9	36

F6HB, Blade	Height (nm)	Width (nm)	N
w/o Pd, RT	30 ± 2	49 ± 2	39
w/ Pd, RT	24 ± 3	59 ± 3	29
w/ Pd, 95 °C	20 ± 3	58 ± 5	36

F10HB, Pole	Length (nm)	N
w/o Pd, RT	37 ± 4	41
w/ Pd, RT	31 ± 4	33
w/ Pd, 95 °C	28 ± 3	64

F10HB, Blade	Height (nm)	Width (nm)	N
w/o Pd, RT	29 ± 2	48 ± 2	41
w/ Pd, RT	24 ± 3	59 ± 4	33
w/ Pd, 95 °C	18 ± 2	63 ± 4	64

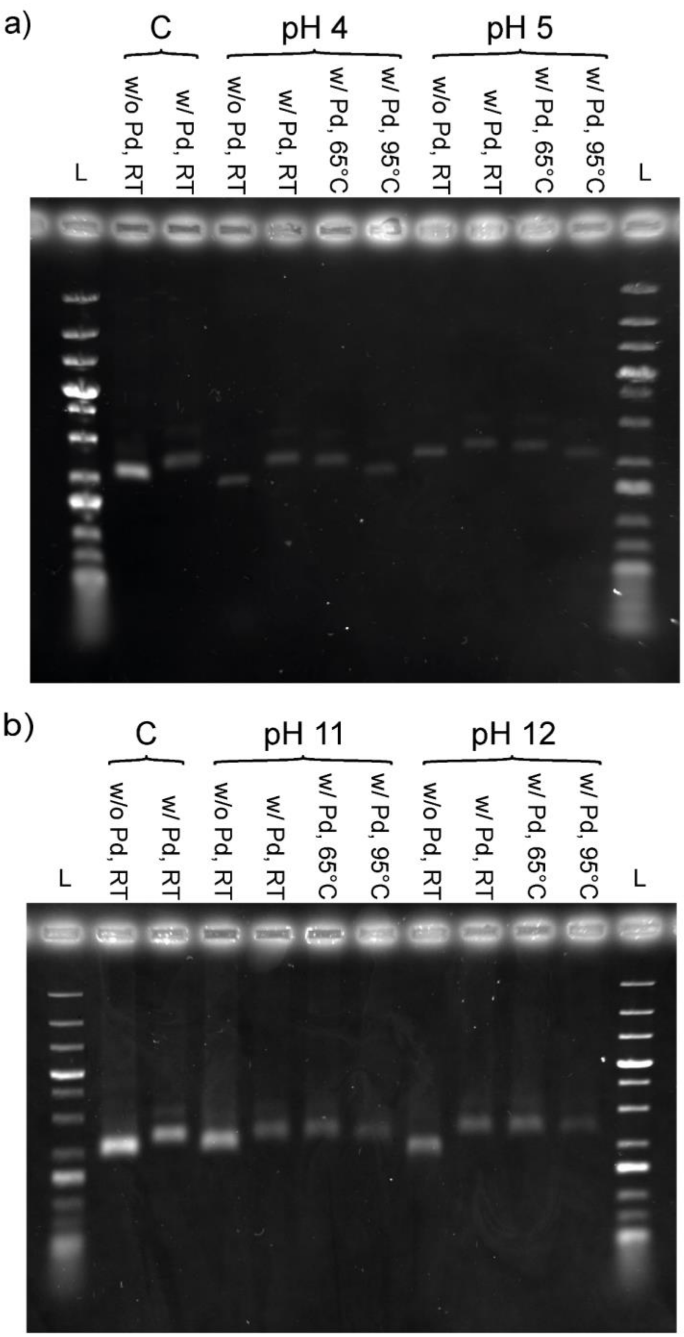

Figure S 27. Agarose gel showing the influence of $[PdCl_4]^{2-}$ on the stability of nanotubes at a 2:1 ratio (bp:Pd ions) at different temperatures at a) pH 4 and 5, and b) pH 11 and 12. Pd treatment, temperatures and pH are denoted above the gel, C is the nanotube control and L indicates a GeneRuler 1 Kb Plus DNA Ladder (Life Technologies GmbH).

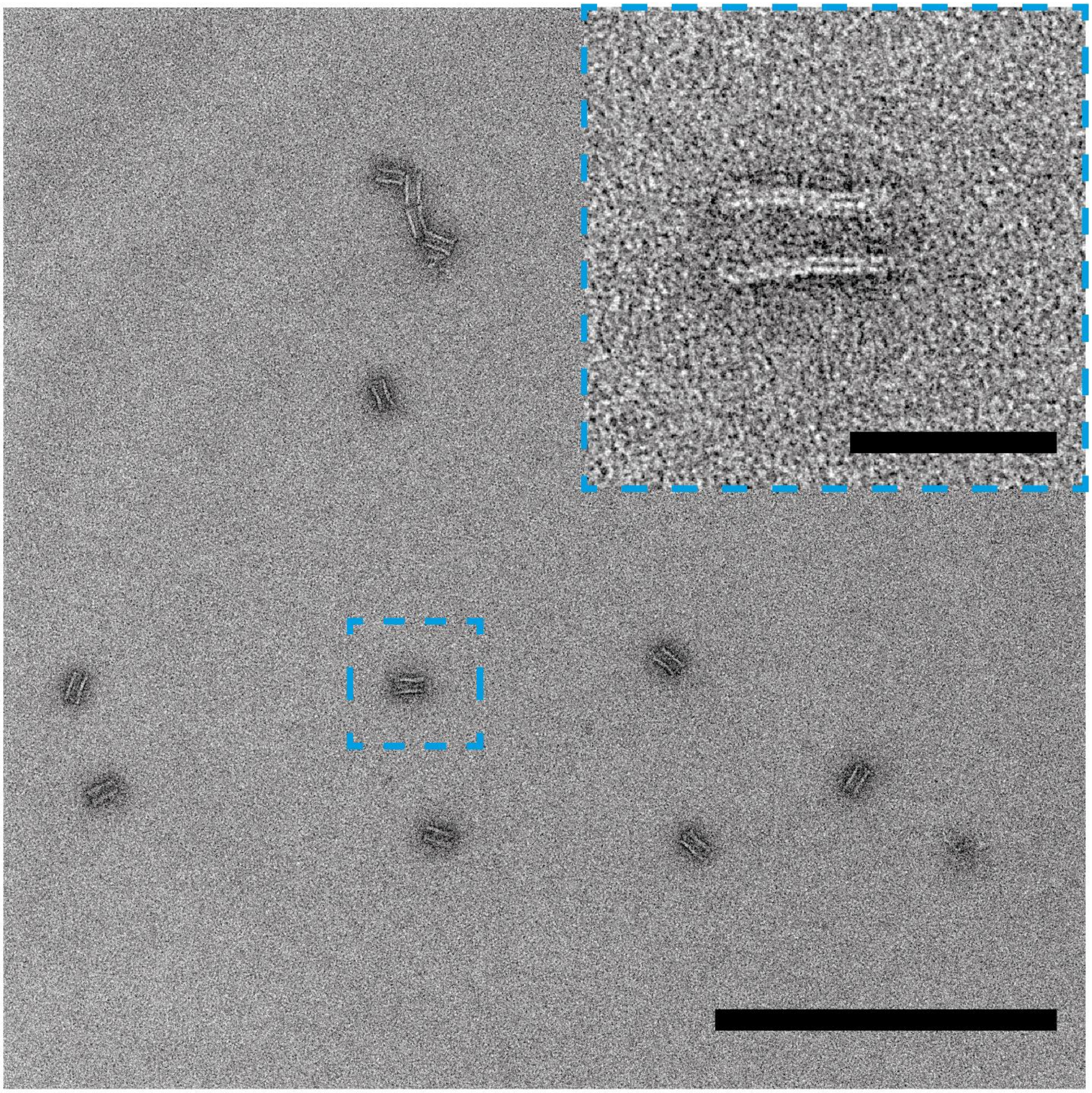

Figure S 28. Overview TEM image of nanotubes in double-distilled water at RT. Scale bar of the overview image equal 500 nm and of the inset 50 nm.

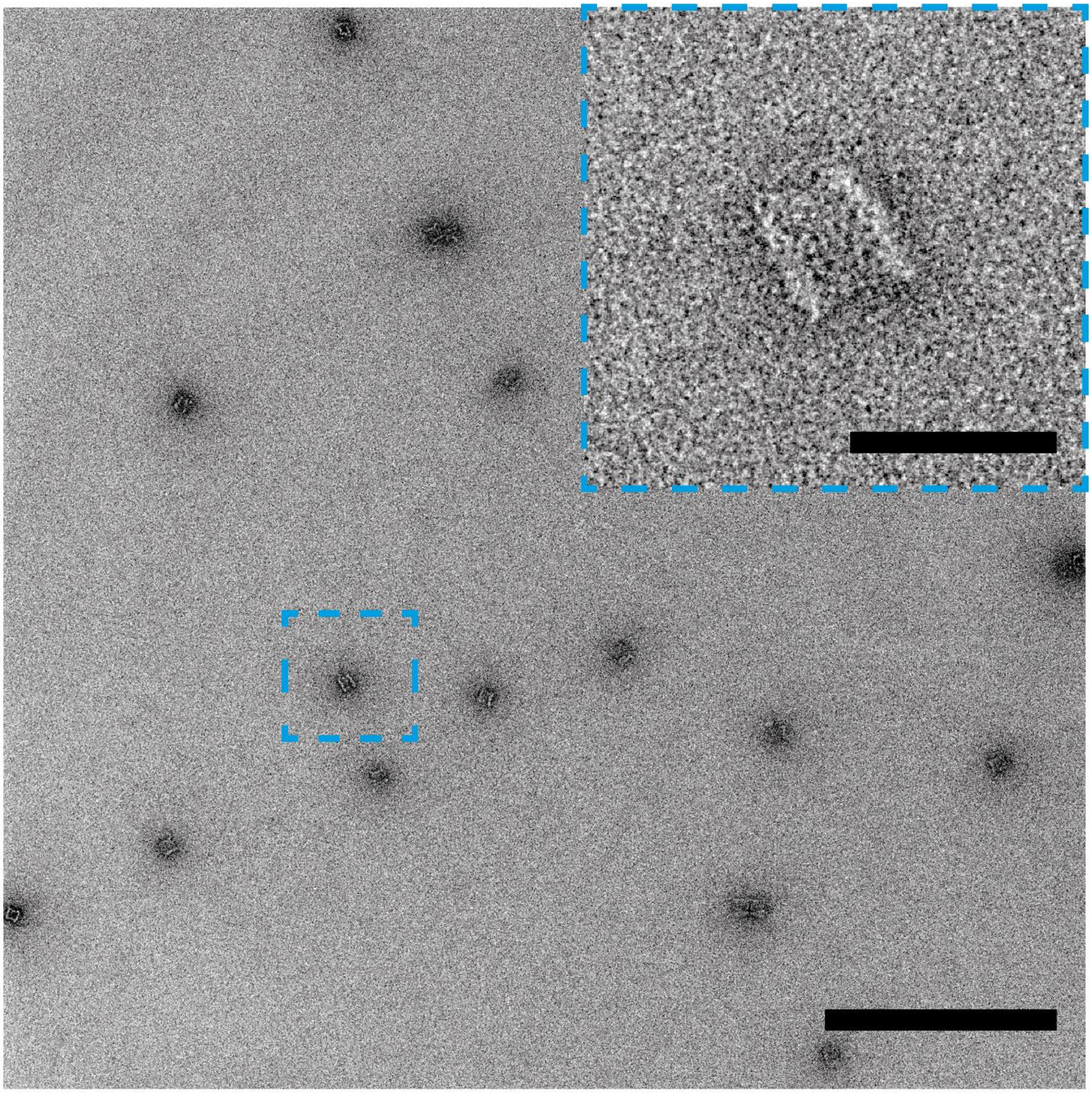

Figure S 29. Overview TEM image of nanotubes stabilized with $[PdCl_4]^{2-}$ at a 2:1 ratio (bp:Pd ions) in double-distilled water at RT. Scale bar of the overview image equal 500 nm and of the inset 50 nm.

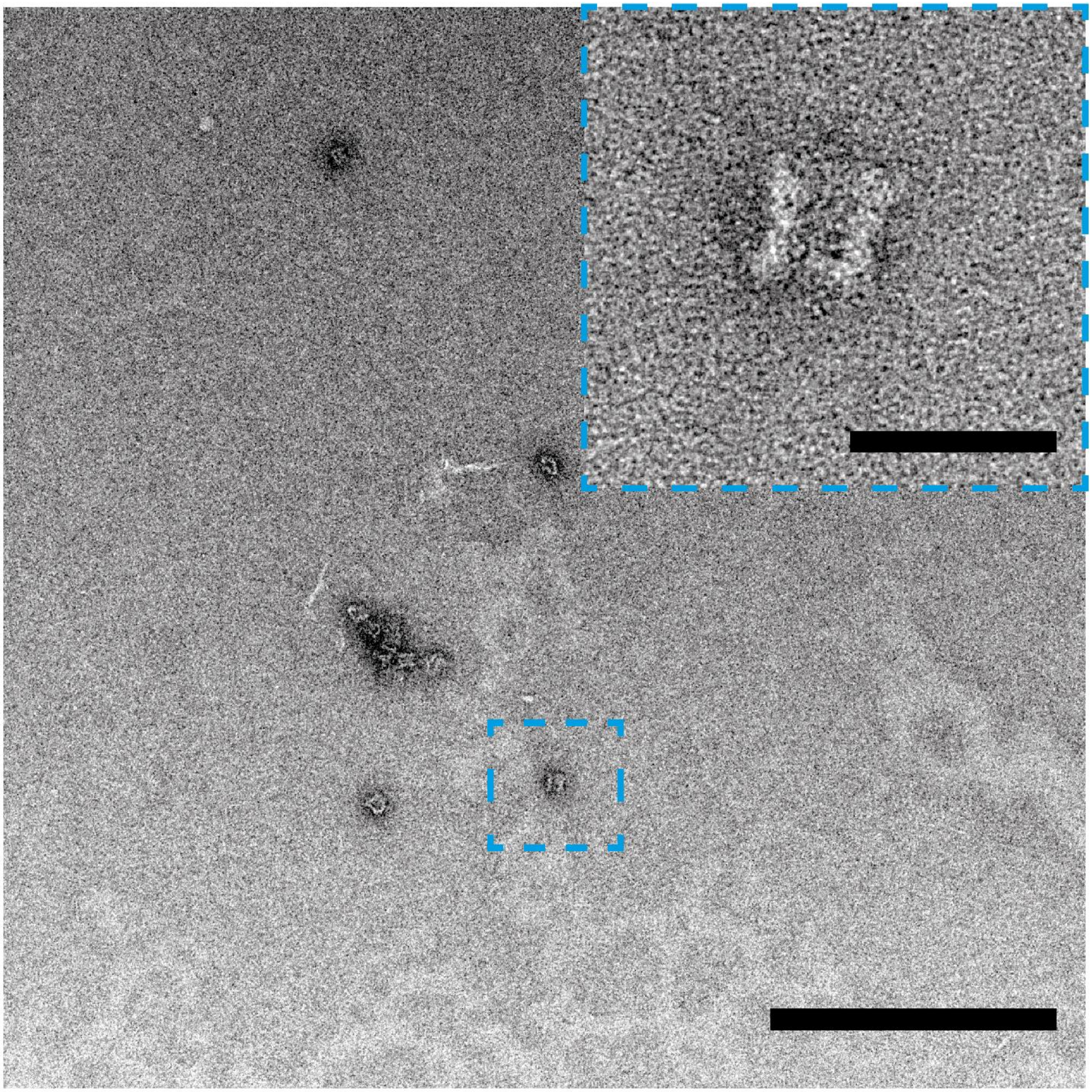

Figure S 30. Overview TEM image of nanotubes stabilized with $[PdCl_4]^{2-}$ at a 2:1 ratio (bp:Pd ions) in double-distilled water at 95 °C. Scale bar of the overview image equal 500 nm and of the inset 50 nm.

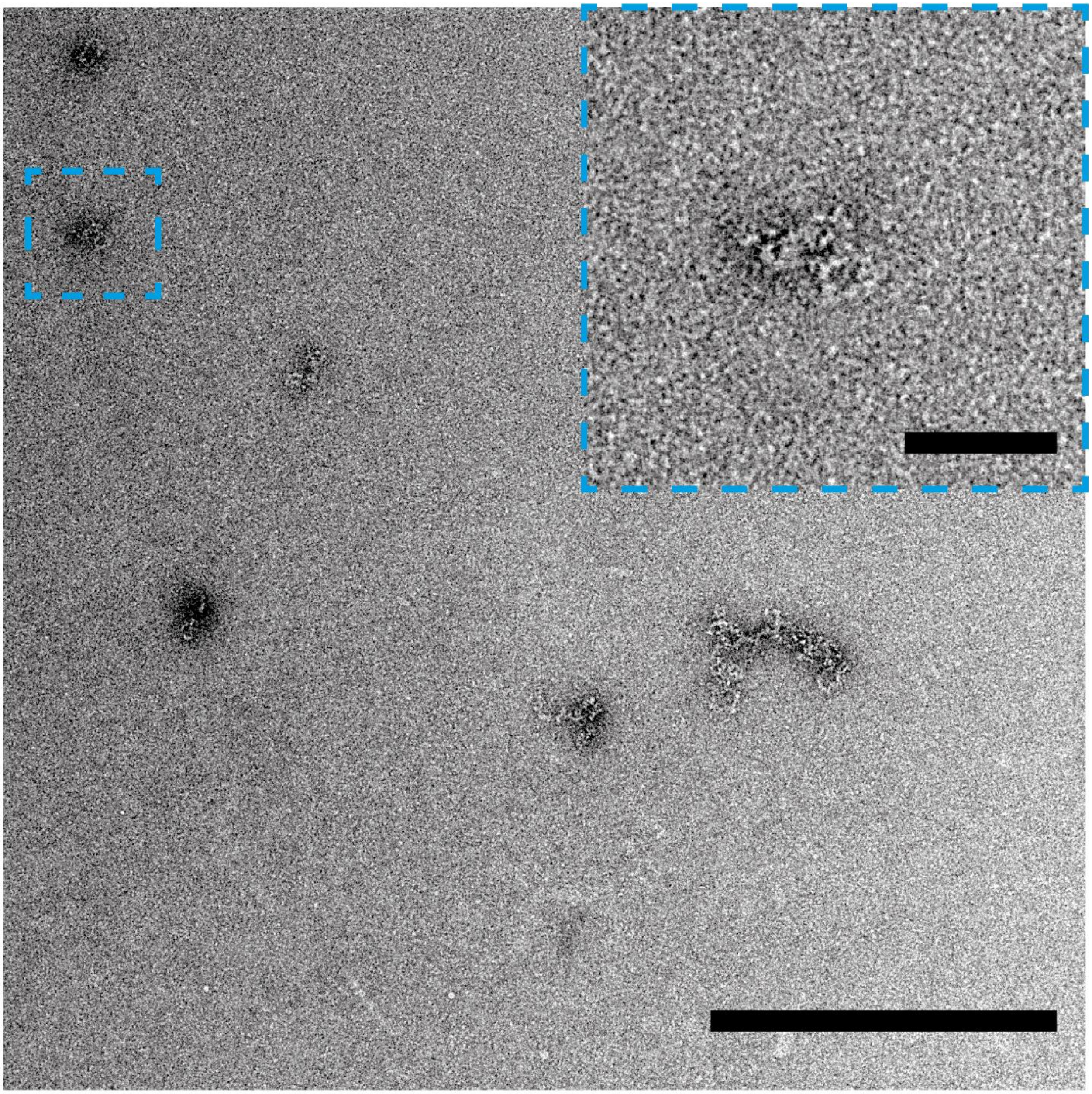

Figure S 31. Overview TEM image of nanotubes at pH 4 at RT. Scale bar of the overview image equal 500 nm and of the inset 50 nm.

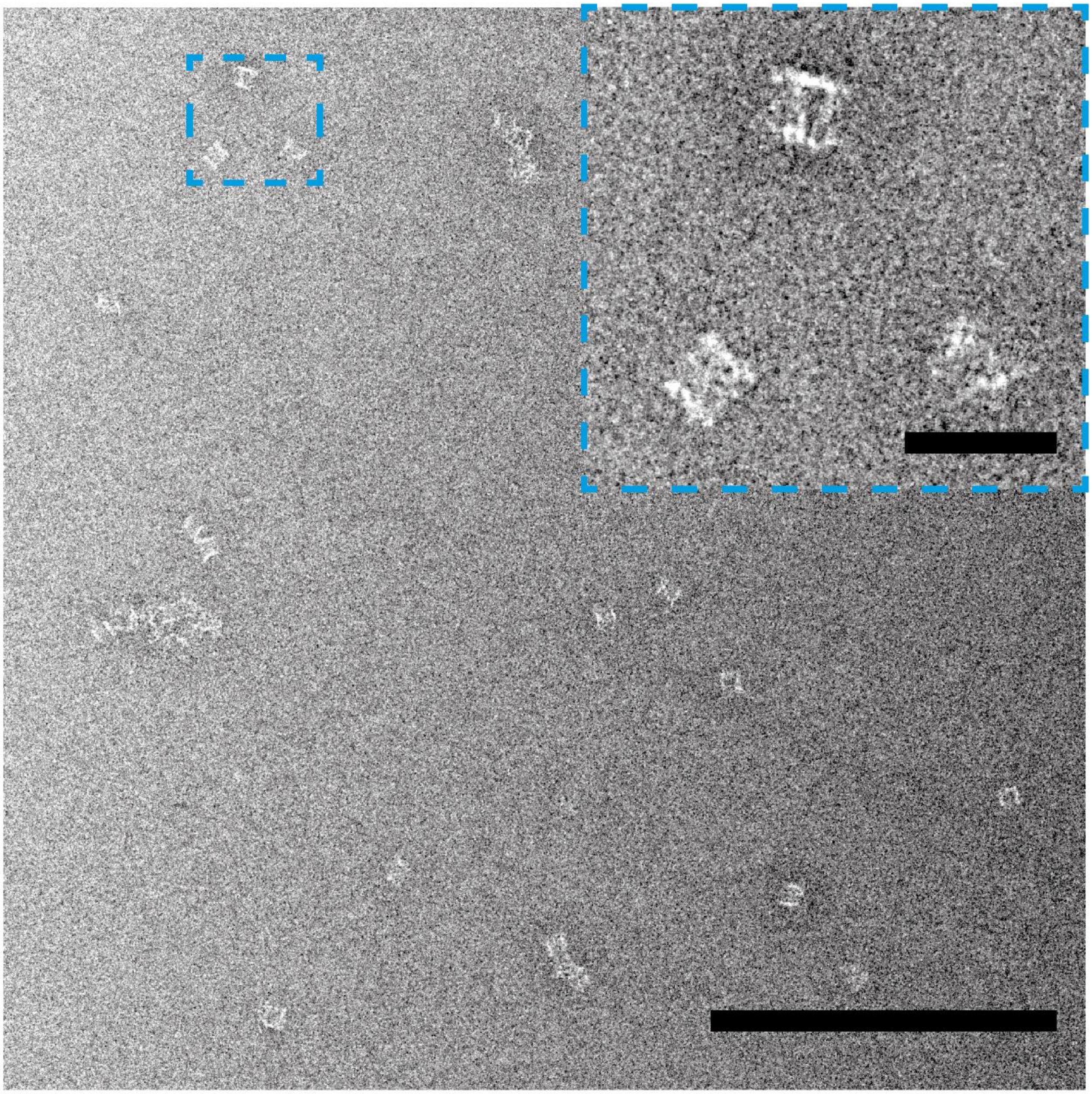

Figure S 32. Overview TEM image of nanotubes stabilized with $[PdCl_4]^{2-}$ at a 2:1 ratio (bp:Pd ions) at pH 4 at RT. Scale bar of the overview image equal 500 nm and of the inset 50 nm.

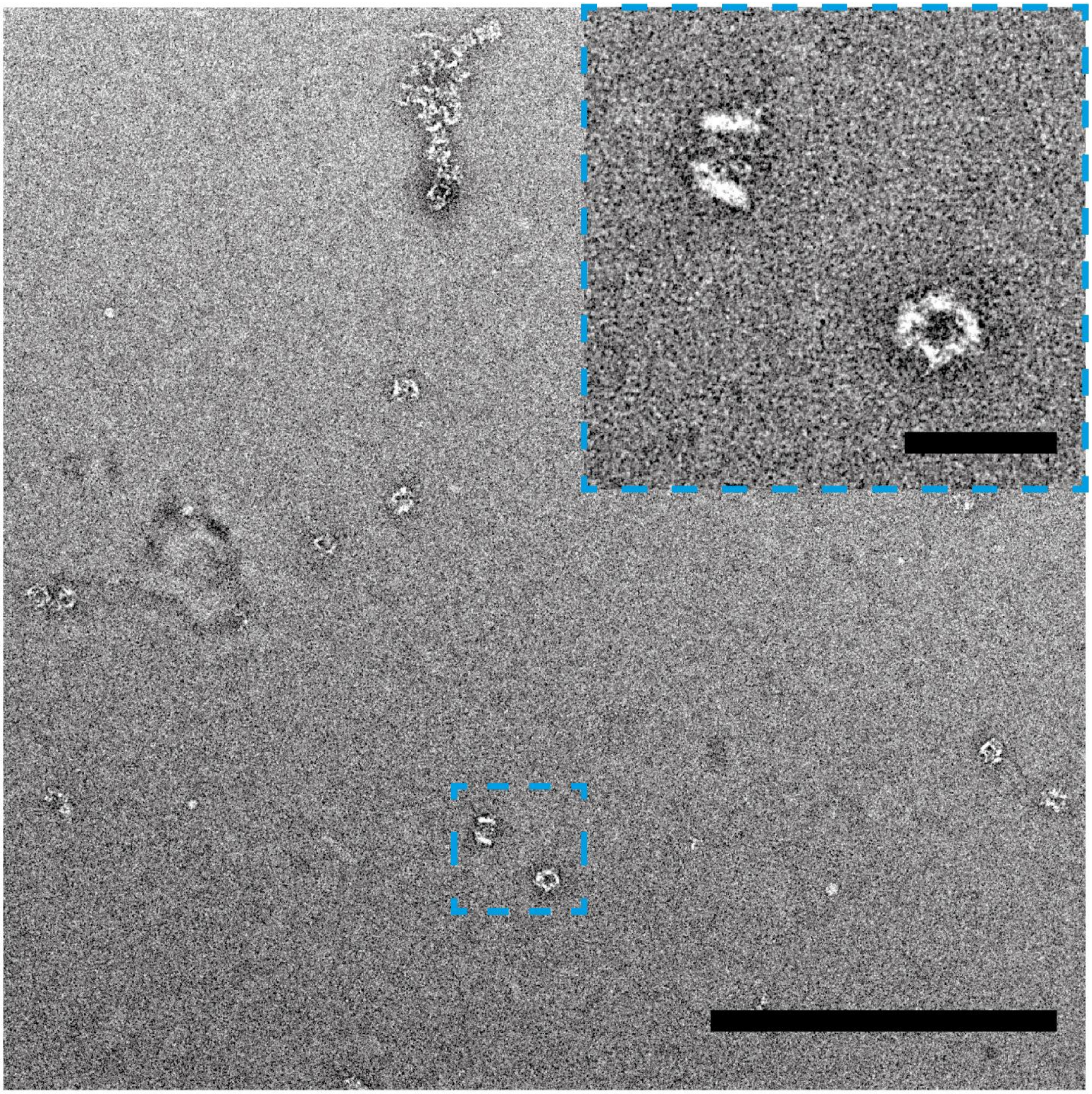

Figure S 33. Overview TEM image of nanotubes stabilized with $[PdCl_4]^{2-}$ at a 2:1 ratio (bp:Pd ions) at pH 4 at 95 °C. Scale bar of the overview image equal 500 nm and of the inset 50 nm.

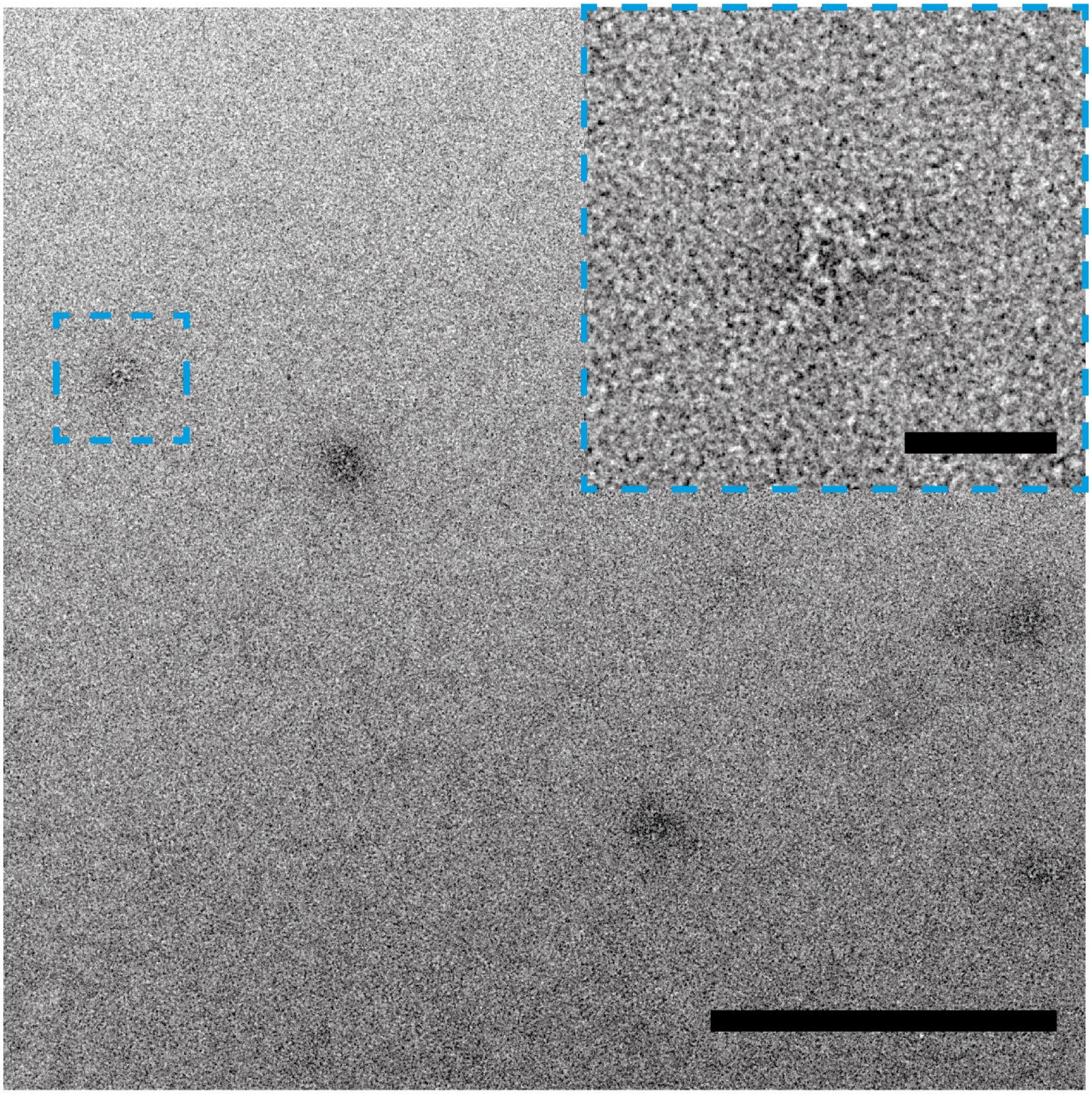

Figure S 34. Overview TEM image of nanotubes at pH 12 at RT. Scale bar of the overview image equal 500 nm and of the inset 50 nm.

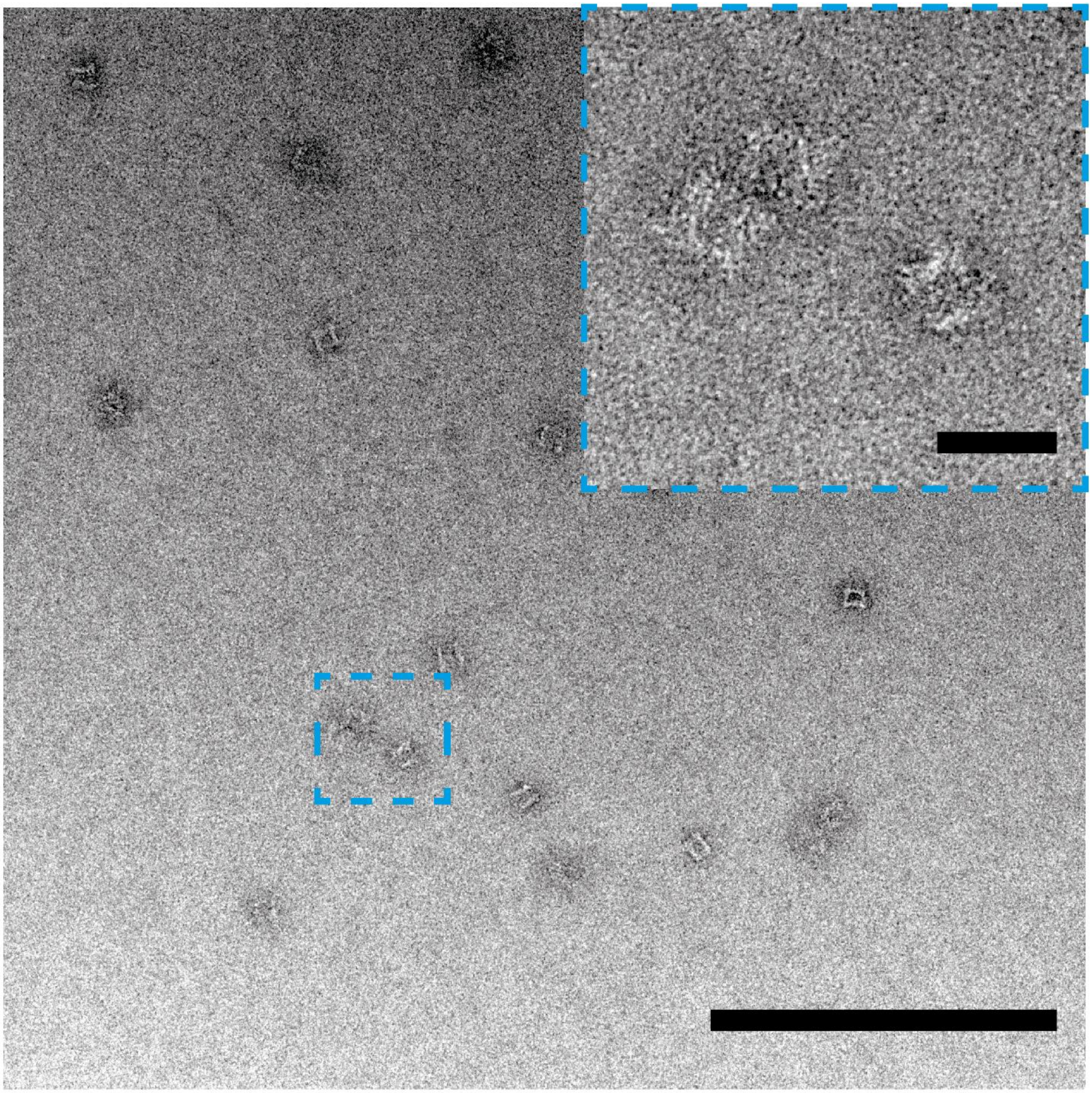

Figure S 35. Overview TEM image of nanotubes stabilized with $[PdCl_4]^{2-}$ at a 2:1 ratio (bp:Pd ions) at pH 12 at RT. Scale bar of the overview image equal 500 nm and of the inset 50 nm.

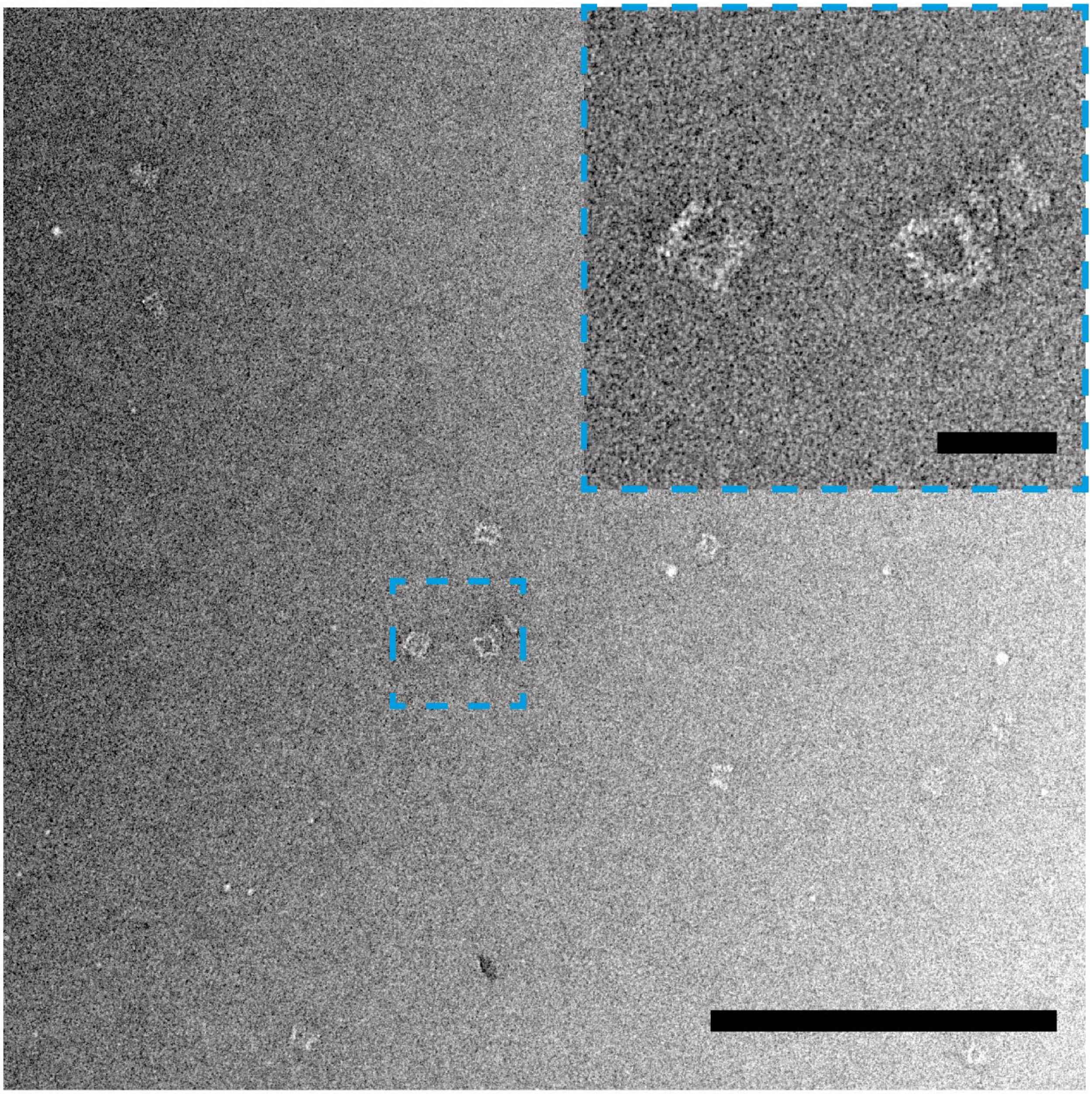

Figure S 36. Overview TEM image of nanotubes stabilized with $[PdCl_4]^{2-}$ at a 2:1 ratio (bp:Pd ions) at pH 12 at 95 °C. Scale bar of the overview image equal 500 nm and of the inset 50 nm.

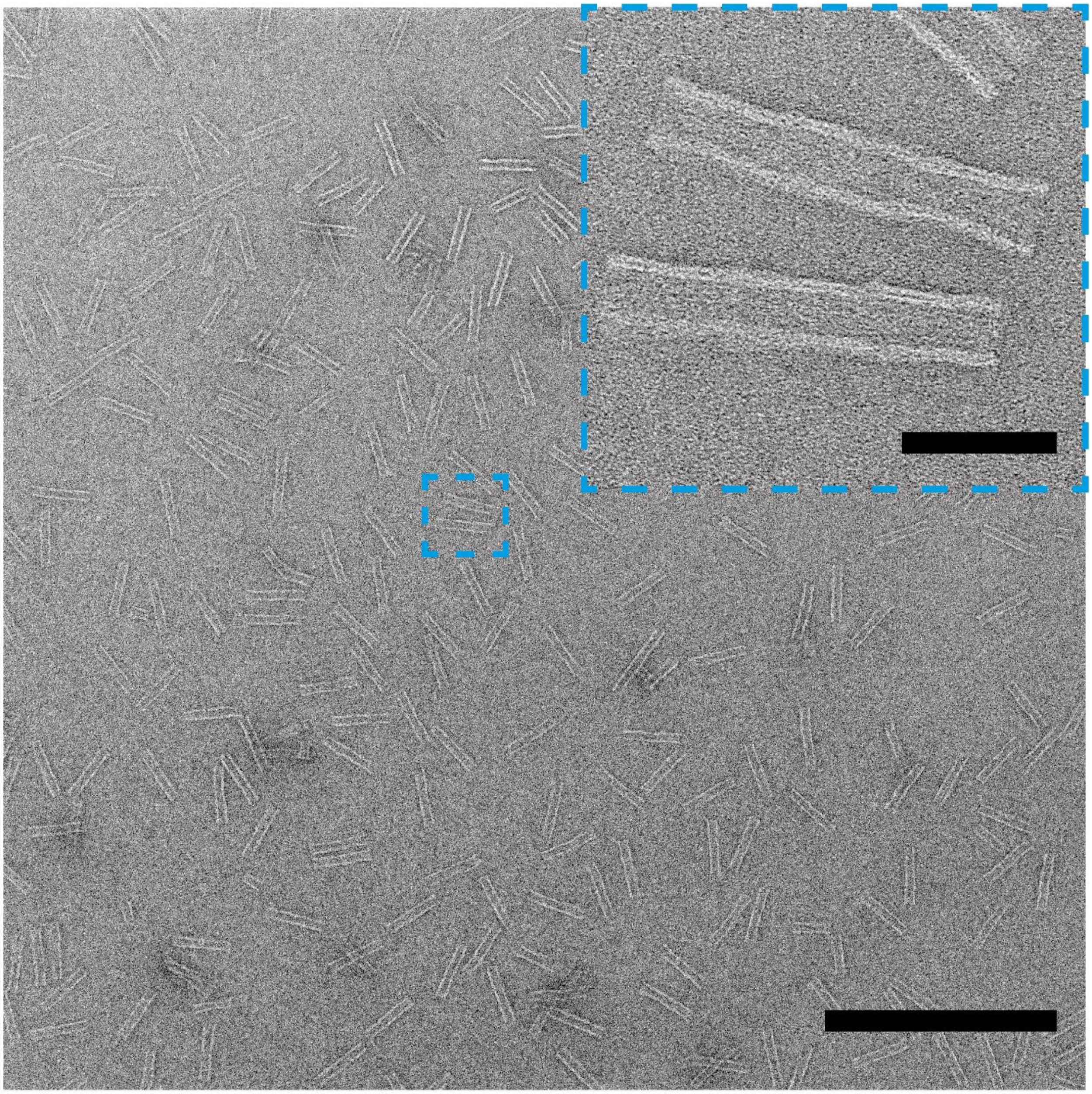

Figure S 37. Overview TEM image of nanotube trimers at RT. Scale bar of the overview image equal 500 nm and of the inset 50 nm.

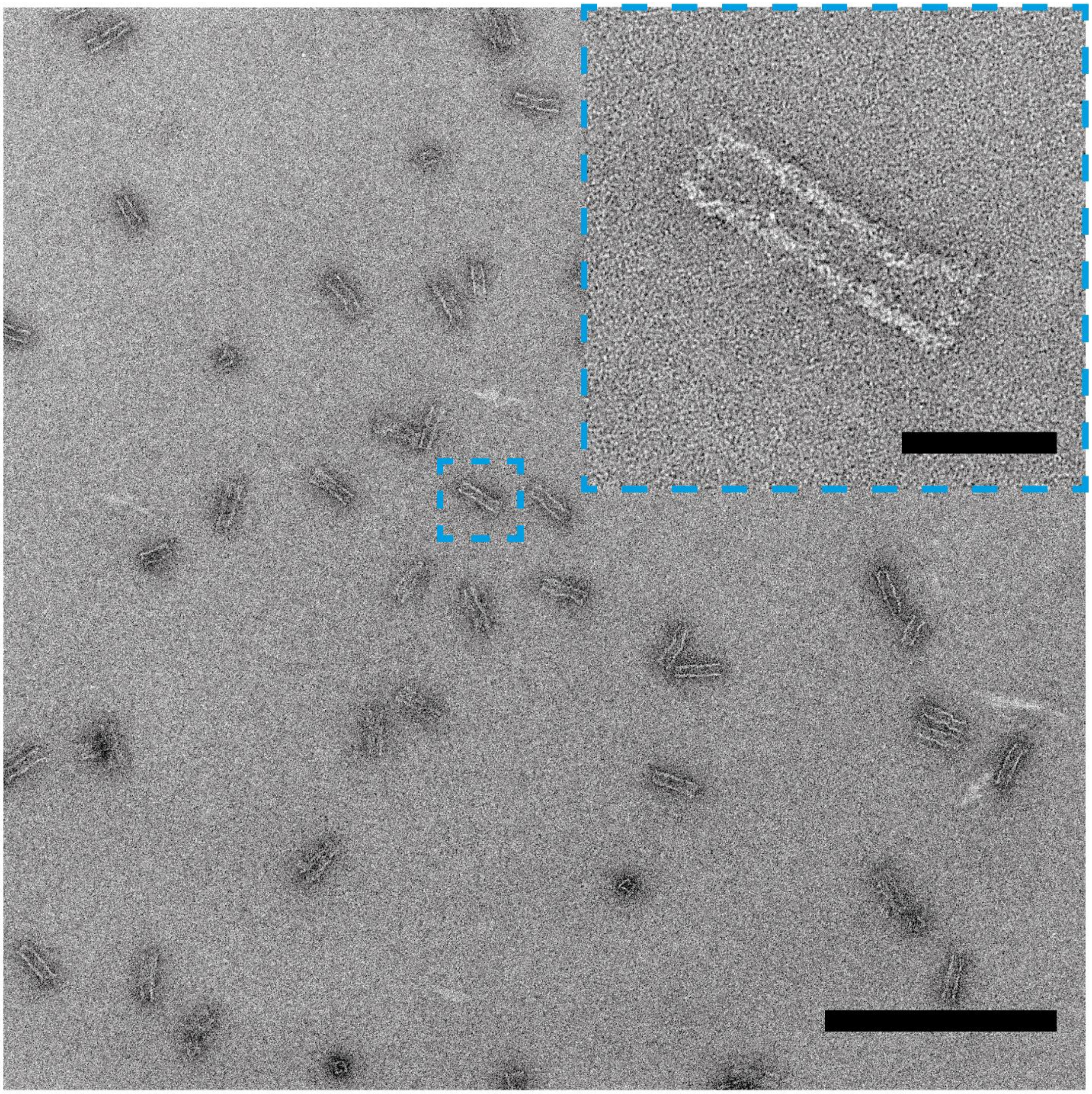

Figure S 38. Overview TEM image of nanotube trimers stabilized with $[PdCl_4]^{2-}$ at a 2:1 ratio (bp:Pd ions) at RT. Scale bar of the overview image equal 500 nm and of the inset 50 nm.

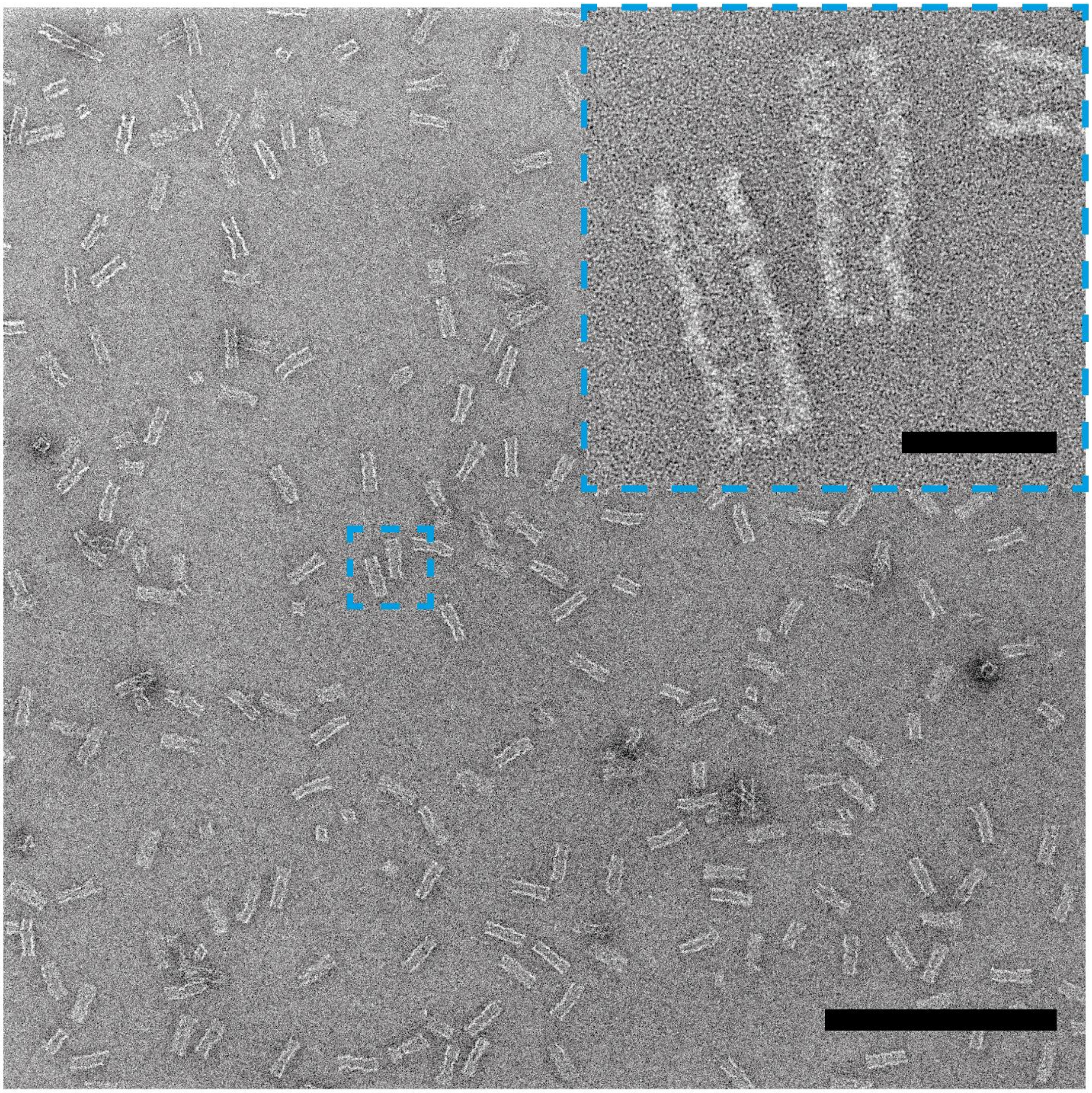

Figure S 39. Overview TEM image of nanotube trimers stabilized with $[PdCl_4]^{2-}$ at a 2:1 ratio (bp:Pd ions) at 95 °C. Scale bar of the overview image equal 500 nm and of the inset 50 nm.

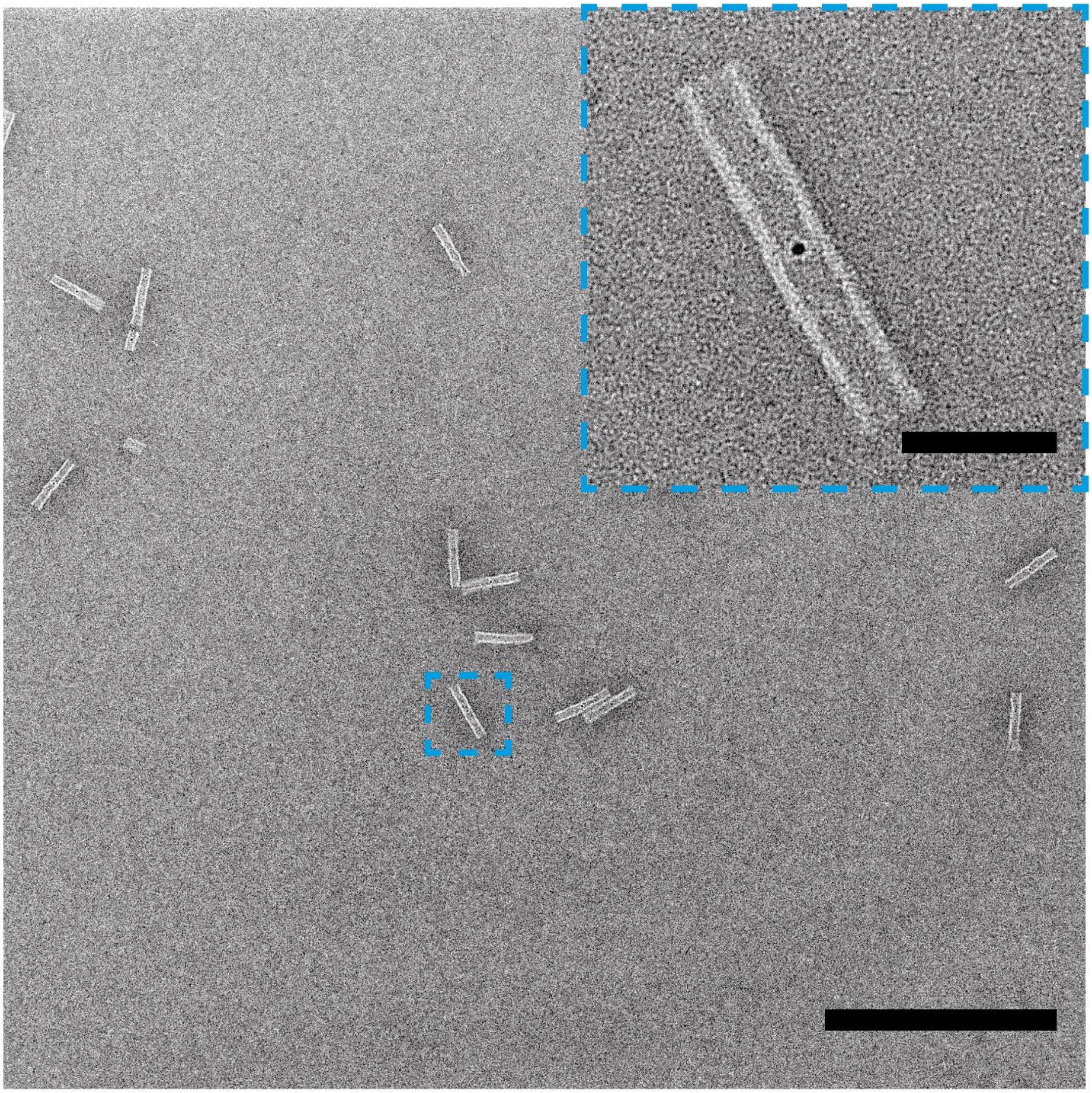

Figure S 40. Overview TEM image of AuNP loaded nanotube trimers at RT. Scale bar of the overview image equal 500 nm and of the inset 50 nm.

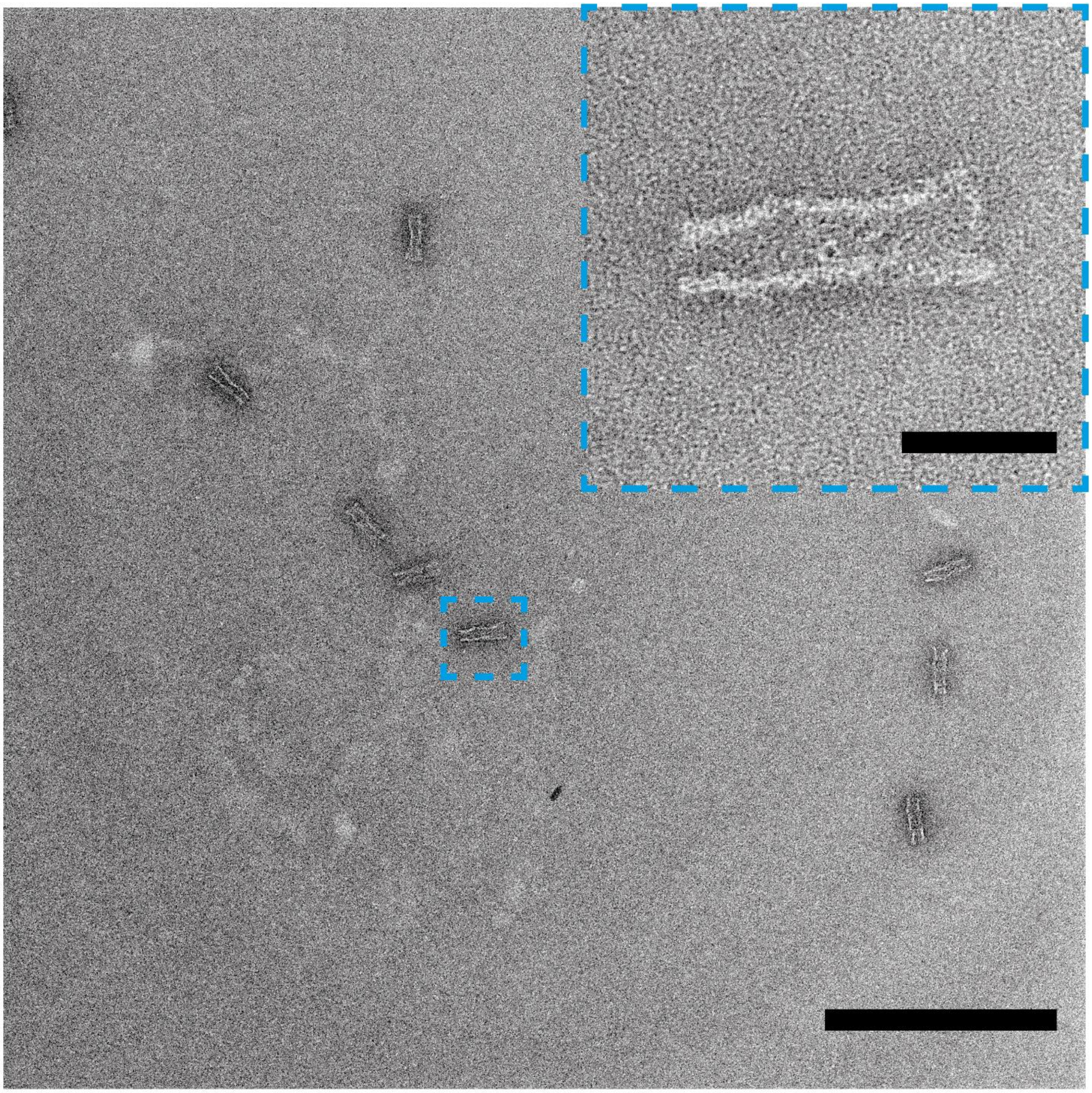

Figure S 41. Overview TEM image of AuNP loaded nanotube trimers stabilized with $[PdCl_4]^{2-}$ at a 2:1 ratio (bp:Pd ions) at RT. Scale bar of the overview image equal 500 nm and of the inset 50 nm.

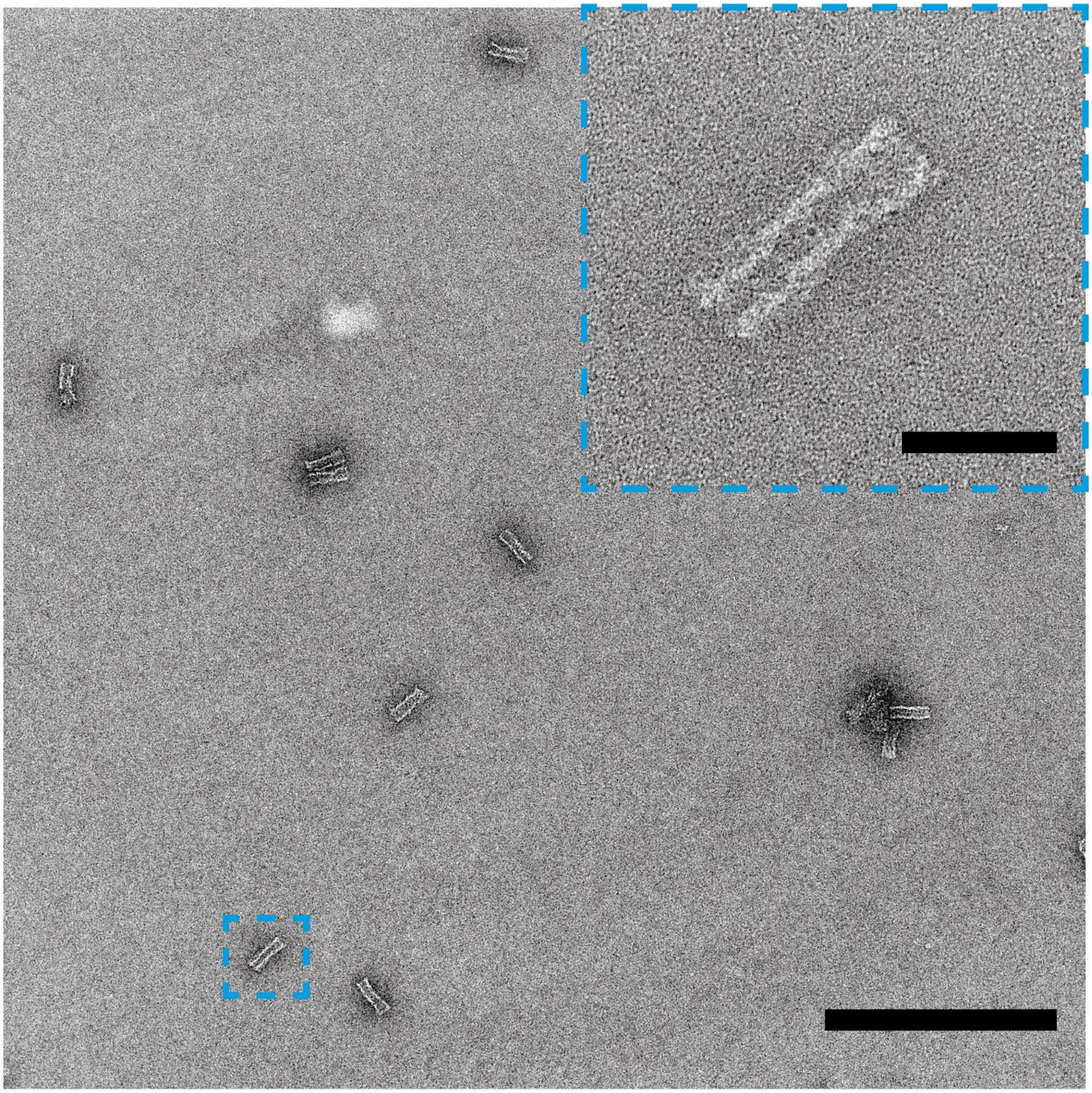

Figure S 42. Overview TEM image of AuNP loaded nanotube trimers stabilized with $[PdCl_4]^{2-}$ at a 2:1 ratio (bp:Pd ions) at 95 °C. Scale bar of the overview image equal 500 nm and of the inset 50 nm.

Table S 4. Attachment yield of AuNPs to DNA origami tube trimers.

	Yield (%)	N
w/o Pd, RT	98 ± 5	131
w/ Pd, RT	97 ± 4	152
w/ Pd, 95 °C	96 ± 9	108

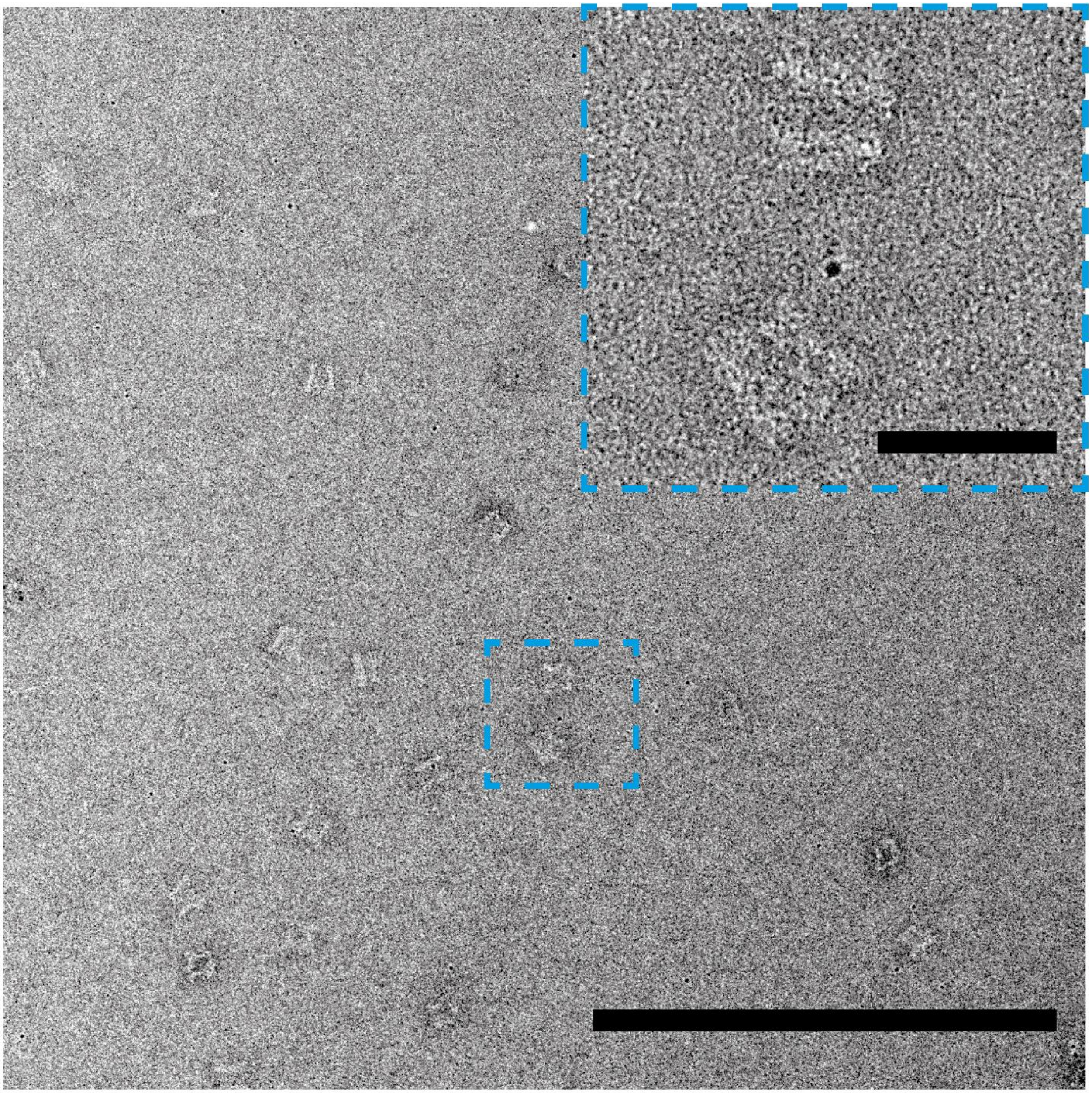

Figure S 43. Overview TEM image of $[PdCl_4]^{2-}$ stabilized nanotubes incubated with AuNPs. Scale bar of the overview image equal 500 nm and of the inset 50 nm.

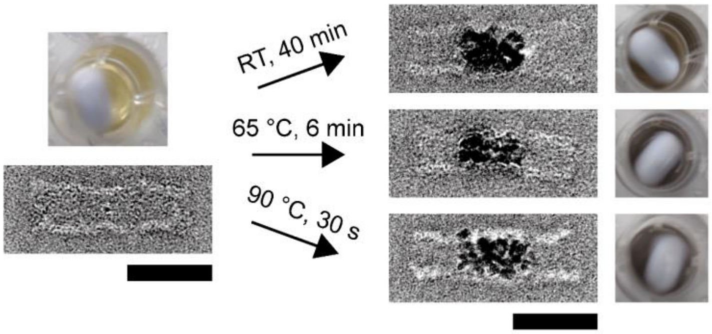

Figure S 44. Pd metallization of Pd stabilized Trimers. TEM images show the grown Pd nanostructures inside the tubes cavities and the photos of the reaction vessels display the color change of the growth solution obtained at different times for different temperatures. Scale bars equal 50 nm.

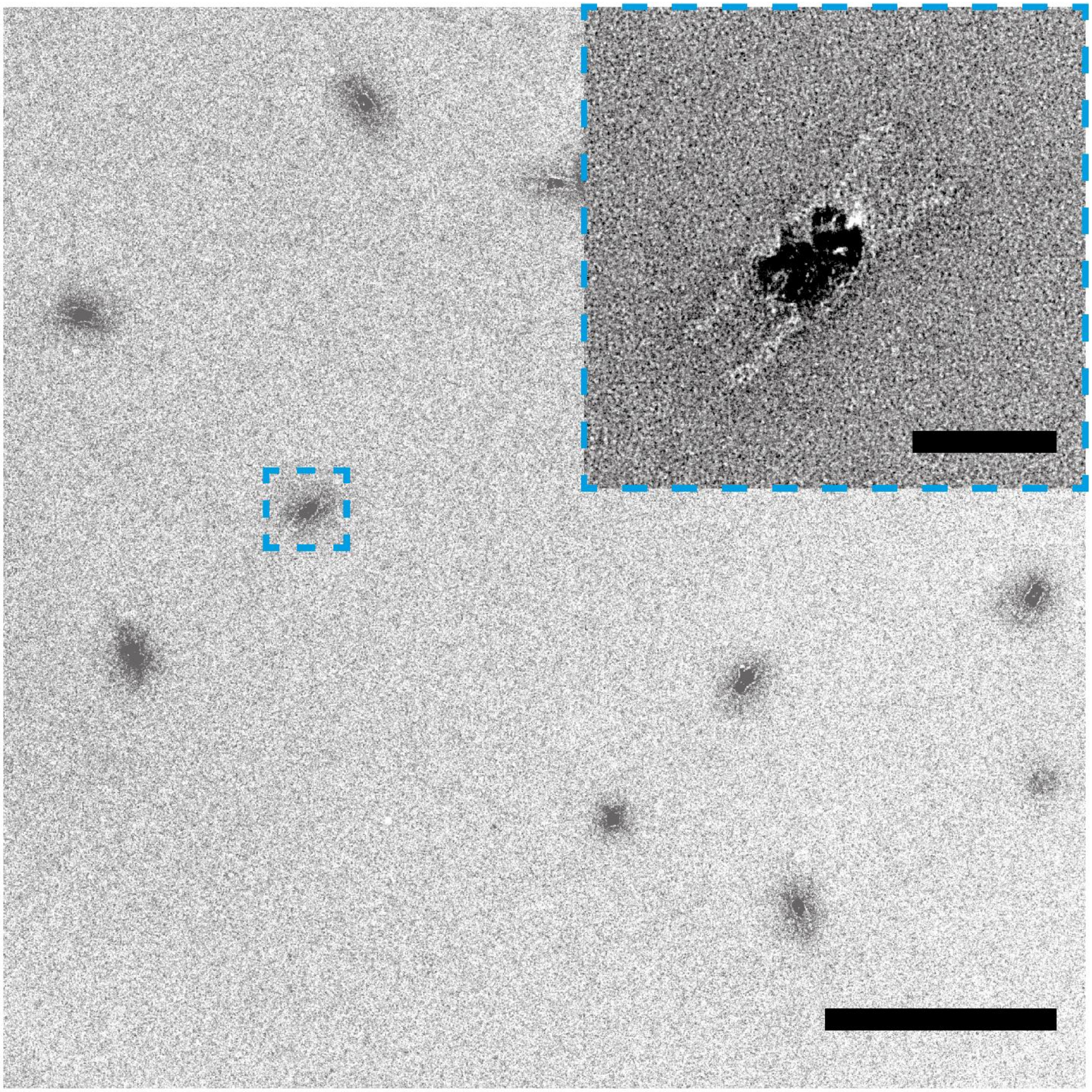

Figure S 45. Overview TEM image of nanotube trimers after Pd growth at RT. Scale bar of the overview image equal 500 nm and of the inset 50 nm.

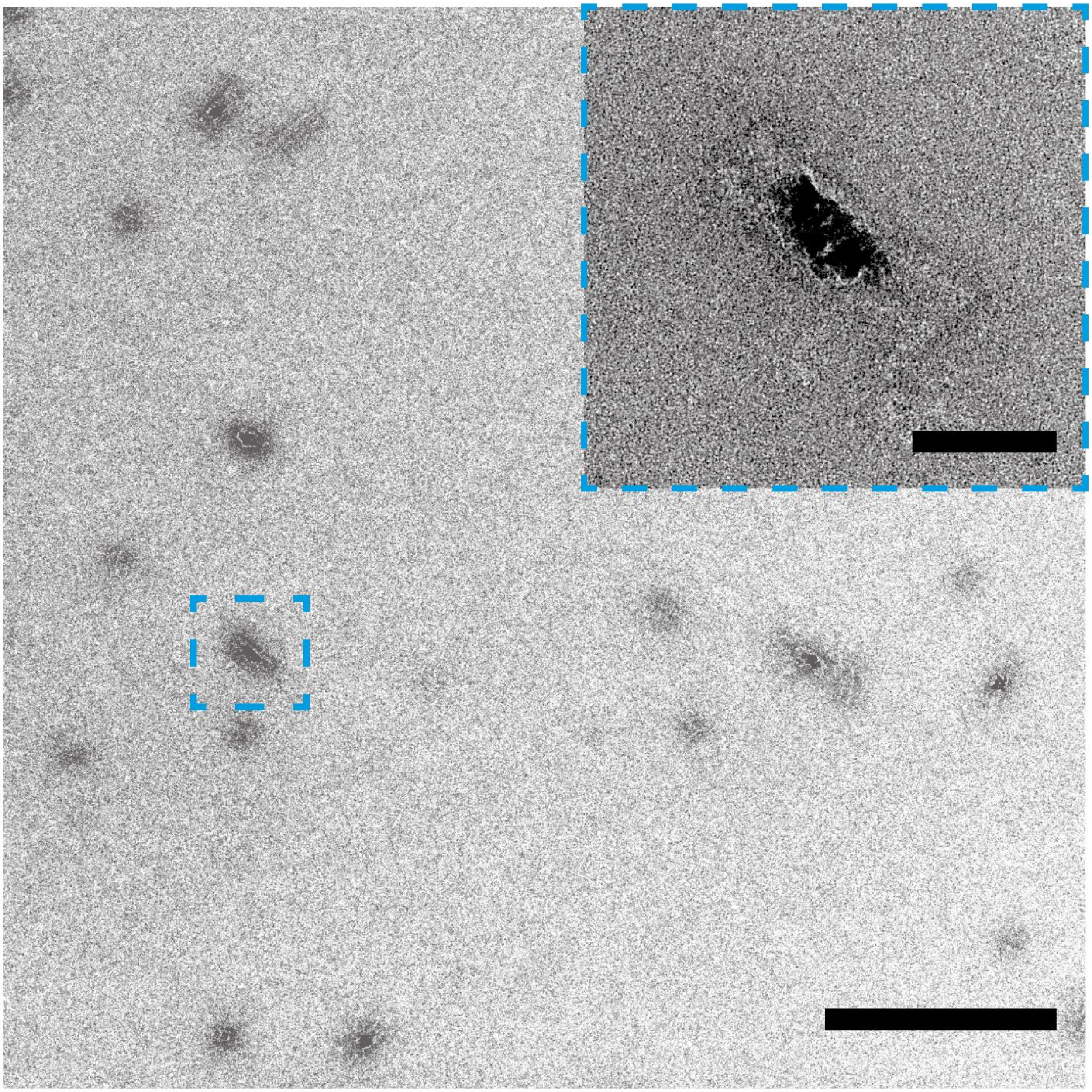

Figure S 46. Overview TEM image of nanotube trimers after Pd growth at 65 °C. Scale bar of the overview image equal 500 nm and of the inset 50 nm.

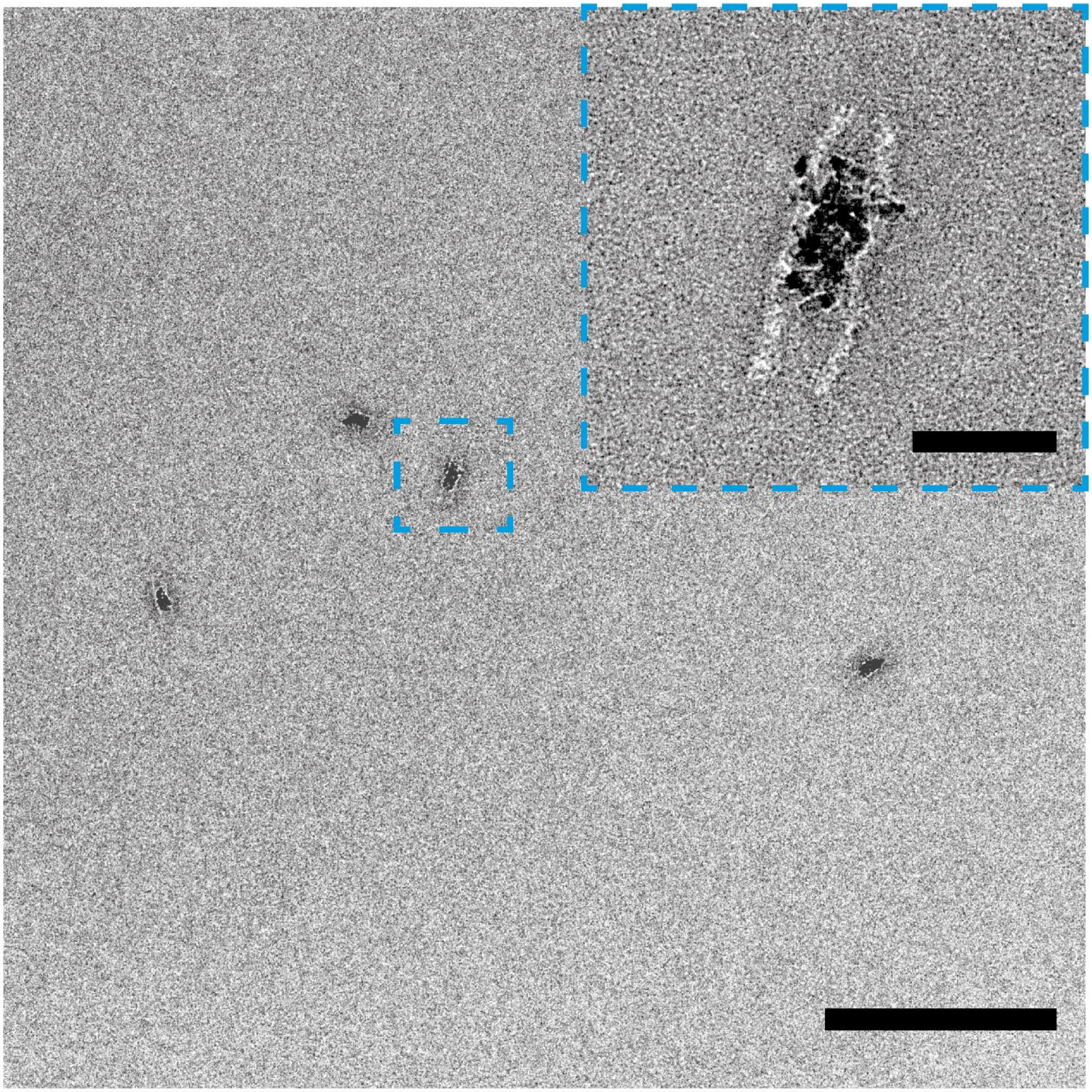

Figure S 47. Overview TEM image of nanotube trimers after Pd growth at 90 °C. Scale bar of the overview image equal 500 nm and of the inset 50 nm.
